# Comparative Functional Genome Analysis Reveals the Habitat Adaptation and Biocontrol Characteristics of Plant Growth-Promoting Bacteria in NCBI Databases

**DOI:** 10.1128/spectrum.05007-22

**Published:** 2023-04-26

**Authors:** Zhen Wang, Kaiheng Lu, Xuan Liu, Yuping Zhu, Changhong Liu

**Affiliations:** a State Key Laboratory of Pharmaceutical Biotechnology, School of Life Sciences, Nanjing University, Nanjing, China; Agroscope

**Keywords:** PGPB, biocontrol agents, habitat adaptation, CAZymes, secondary metabolites

## Abstract

Plant growth-promoting bacteria (PGPB) are a group of beneficial microorganisms that include 60 bacterial genera, such as *Bacillus*, Pseudomonas, and *Burkholderia*, which widely colonize plant leaves and soil, promote plant growth, and/or inhibit pathogen infection. However, the genetic factors underpinning adaptation of PGPB to plant leaves and soil remain poorly understood. In this study, we performed a comparative functional genome analysis approach to investigate the functional genes of 195 leaf-associated (LA) and 283 soil-associated (SA) PGPB strains and their roles in adapting to their environment, using 95 strains from other-associated (OA) environmental habitats with growth-promoting or antimicrobial functions as negative controls. Comparison analysis of the enrichment of nonredundant (NR) protein sequence databases showed that cytochrome P450, DNA repair, and motor chemotaxis genes were significantly enriched in LA PGPB strains related to environmental adaptation, while cell wall-degrading enzymes, TetR transcriptional regulatory factors, and sporulation-related genes were highly enriched in SA PGPB strains. Additionally, analysis of carbohydrate-active enzymes demonstrated that glycosyltransferases (GTs) and glycoside hydrolases (GHs) were abundant families in all PGPB strains, which is in favor of plant growth, and enriched in SA PGPB strains. Except for most *Bacillus* strains, SA PGPB genomes contained significantly more secondary metabolism clusters than LA PGPB. Most LA PGPB contained hormone biosynthesis genes, which may contribute to plant growth promotion, while SA PGPB harbored numerous carbohydrate and antibiotic metabolism genes. In summary, this study further deepens our understanding of the habitat adaptation and biocontrol characteristics of LA and SA PGPB strains.

**IMPORTANCE** Plant growth-promoting bacteria (PGPB) are essential for the effectiveness of biocontrol agents in plant phyllosphere and rhizosphere. However, little is known about the ecological adaptation of PGPB to different habitats. In this study, comparative functional genome analysis of leaf-associated (LA), soil-associated (SA), and other-associated (OA) PGPB strains was performed. We found that genes related to the metabolism of hormones were enriched in LA PGPB. Carbohydrate and antibiotic metabolism genes were enriched in SA PGPB, which likely facilitated their adaptation to the plant growth environment. Our findings provide genetic insights on LA and SA PGPB strains’ ecological adaptation and biocontrol characteristics.

## INTRODUCTION

Plant growth-promoting bacteria (PGPB) are a beneficial group of microorganisms that colonize plant leaves and soil, promoting plant growth and/or inhibiting pathogen infection. As a sustainable alternative to or partial replacement for chemically synthesized fungicides that pose potential environmental risks in agriculture, PGPB can serve as biocontrol agents (1–3). According to previous studies, a total of 478 PGPB strains have been reported (4). Leaf-associated (LA) PGPB are often capable of synthesizing plant hormones and widely used in the development of plant growth regulators (5). In contrast, soil-associated (SA) PGPB are generally rich in carbohydrates and secondary metabolite biosynthesis genes and widely used in the development of biopesticides (6, 7). However, the unique genetic characteristics and environmental adaptation mechanisms of these LA and SA PGPB are still unclear.

PGPB have multiple mechanisms to enhance plant growth and induce disease resistance, including the use of plant hormones, antioxidant enzymes, carbohydrate enzymes (CAZymes), and antibiotics (8, 9). The CAZymes are enzymes that break down the cell walls of plant pathogens, leading to pathogen death. PGPB can also produce bioactive secondary metabolites, such as antibiotics (10), including nonribosomal peptides (NRPs) and polyketides (PKs) synthesized by nonribosomal peptide synthetase (NRPS) (11). Nevertheless, the genes and metabolic pathways of PGPB from plant leaves and soil, including those related to plant hormones, carbohydrates, and secondary metabolite clusters, are still not fully understood.

Comparative functional genome analysis is an effective approach for revealing the evolution and habitat adaptation mechanisms of microorganisms (12–14) and the diverse metabolic capabilities of strains within the same species isolated from different habitats (15). The aim of this study was to use comparative functional genome analysis to uncover the genetic characteristics and metabolic differences of PGPB residing in leaves and root zone soils, providing insights into the biological and ecological functions of PGPB and their applications in agriculture.

## RESULTS

### Collection of strains, genomic features, and phylogenetic analysis.

**FIG 1:**
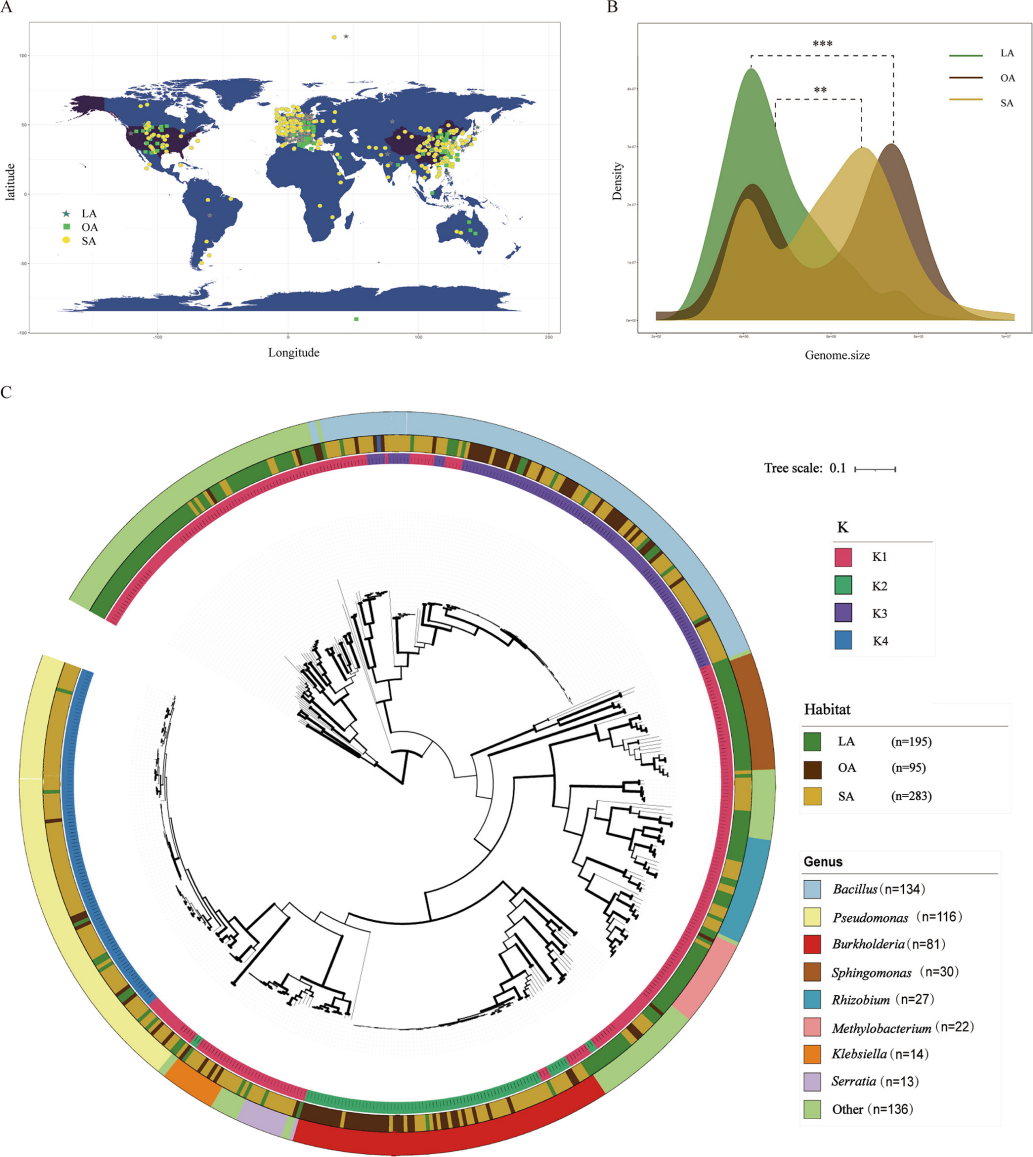
Comparative genomics and phylogenomics of 573 PGPB or PGPB-like strains. (A) Worldwide distribution map of 573 strains. (B) Density plot of the genome sizes of LA, SA and OA strains. Double and triple asterisks indicate a significant (*P < *0.01) and an extremely significant (*P < *0.001) difference between two habitats as inferred from Student’s *t* test, respectively. (C) Maximum likelihood phylogenetic tree of 573 high-quality and nonredundant bacterial genomes, based on the protein sequences of 14 single-copy genes. The outer ring shows bacteria of genera, the central ring shows the habitats, and the inner ring shows the taxonomic group. It is worth noting that subsequent studies have adopted the same color scheme as used here. All data are available in the supplemental material. LA, leaf-associated; SA, soil-associated; OA, other-associated.

We conducted a search for bacterial strains using “plant growth-promoting” and/or “antibacterial” as keywords in the Web of Science platform, which yielded a total of 2,607 strains. Out of these, 1,042 strains had their genomes sequenced and were stored in the NCBI genome and/or protein database. We then employed PYANI, GBDP, and CheckM tools to remove duplicates and obtained a data set of 573 strains (60 genera) with high-quality genome and protein sequences (see Table S1 and Fig. S1 in the supplemental material and method 1 included the redundancy analysis and so on). Among these strains, 478 were plant growth-promoting bacteria (PGPB) that colonized either leaves (leaf associated [LA]; 195 strains) or rhizospheric soil (soil associated [SA]; 283 strains), while the remaining 95 strains were isolated from nonplant environments (other associated [OA]) such as food, air, and the human body, serving as control strains for this study. Notably, these strains were distributed worldwide (Fig. 1A). On analyzing the genome data, we observed that the average genome size of LA PGPB strains was significantly smaller (4.67 Mb) than those of SA PGPB strains (6.08 Mb) and OA strains (5.97 Mb) (*t* test, *P < *0.05) (Fig. 1B). Additionally, the average GC content of LA PGPB strains was significantly higher (63.27%) than those of SA PGPB strains (57.12%) and OA strains (56.95%) (*t* test, *P < *0.05) (Fig. S2).

To investigate the evolutionary relationships among 573 bacterial strains from various habitats, a maximum likelihood phylogenetic tree was generated based on the amino acid sequences of 14 single-copy core genes (Fig. 1C) (method 2). The outer circle of the tree represents eight genera, including *Bacillus* (134 strains), *Pseudomonas* (116 strains), *Burkholderia* (81 strains), *Sphingomonas* (30 strains), *Rhizobium* (27 strains), *Methylobacterium* (22 strains), *Klebsiella* (14 strains), and *Serratia* (13 strains), while the remaining 136 strains from 52 genera are relatively dispersed. Strains belonging to the same genus cluster together on the tree, reflecting their similar evolutionary relationships.

The middle layer of phylogenetic tree represents the clustering information of bacterial strains. Due to the substantial variations in genome size and GC content among strains originating from distinct habitats, we employed the *k*-medoids clustering algorithm to further group the strains based on their similarity of lineage distances, which were converted from the phylogenetic tree of 573 bacteria (method 2). By maximizing the silhouette coefficient of 0.44 (Fig. S3), we identified the optimal *k* value, which resulted in four distinct clusters: K1, K2, K3, and K4. Group K1 consisted of 285 strains from 60 different genera, with 174 LA PGPB strains accounting for 61.05% of the total in K1, 95 SA PGPB strains accounting for 33.33%, and 16 OA strains accounting for 5.61%. Group K2 included 78 strains from 4 genera, predominantly composed of 34 SA PGPB strains (43.59% of K2 total) and 40 OA strains (51.28% of K2 total). Group K3 comprised 108 strains from 2 genera, *Bacillus* and *Paenibacillus*, with the majority being 66 SA PGPB strains (61.11% of K3 total). Group K4 was composed of 102 strains from the *Pseudomonas* genus, with 88 SA PGPB strains (86.27% of K4 total) (Fig. 1C, Table 1, and Fig. S4).

**TABLE 1:** Habitats and taxa of all strains

Classification	No. of strains			
	LA	SA	OA	Sum
K1	174	95	16	285
K2	4	34	40	78
K3	9	66	33	108
K4	8	88	6	102
Sum	195	283	95	573

The innermost circle of the tree represents habitat information of strains collected in the literature and NCBI databases (Fig. 1C). Given the extensive data and various sources, it is prudent to ascertain the statistical significance of the classification of PGPB compartments in the literature and databases. We applied a hierarchical clustering method with complete linkage to cluster 573 bacterial strains based on the number of CAZyme and secondary metabolite cluster genes per megabase of genome. We found that a total of 168 LA PGPB strains (29.32% of all strains), 251 SA PGPB strains (43.8% of all strains), and 81 OA strains (14.14% of all strains) clustered together (method 2).

### Comparison of core genome and functions of PGPB in different habitats.

**FIG 2:**
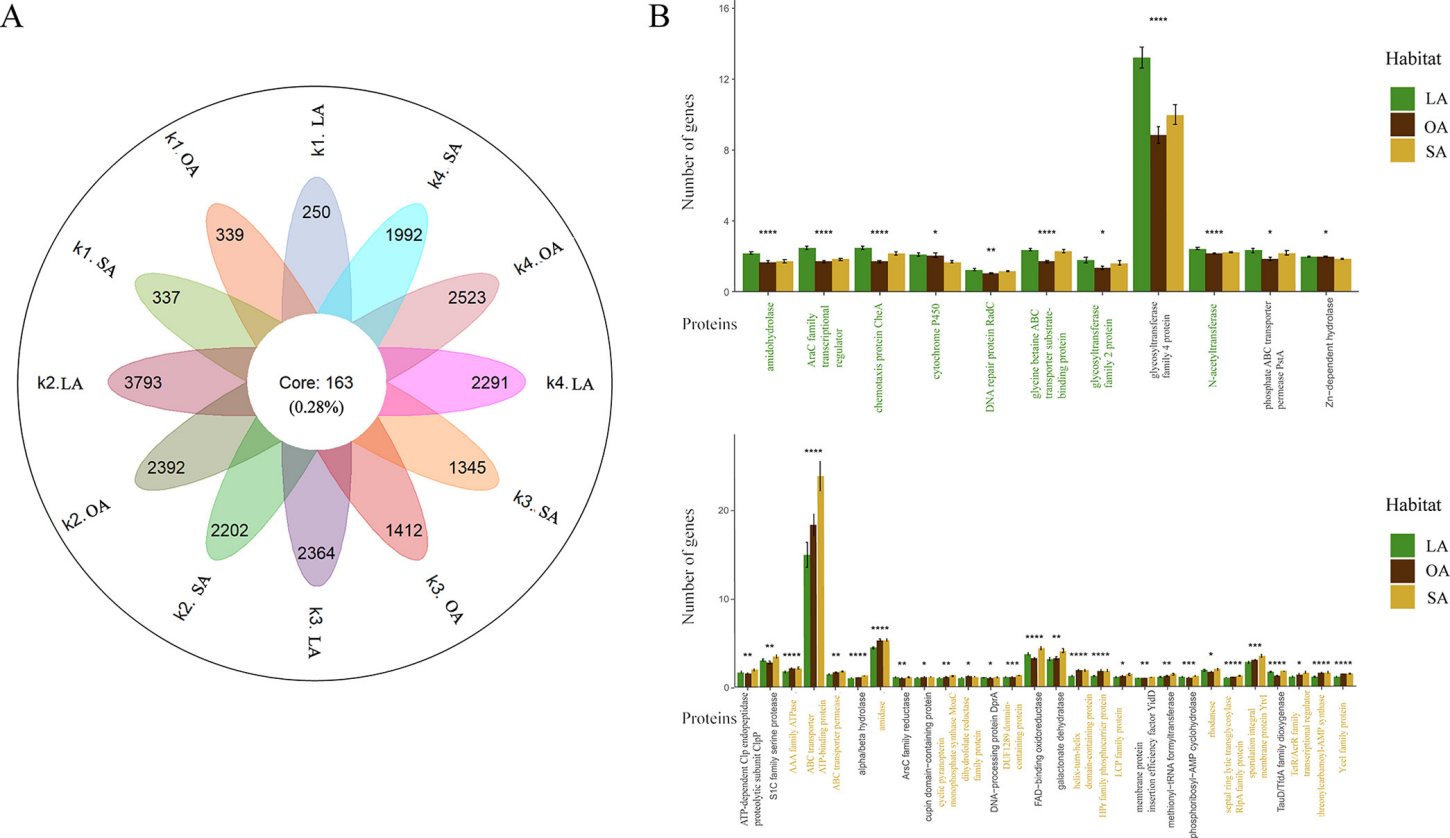
Obtaining the core genome and comparing its functional characteristics across three habitats in all strains. (A) A flower plot depicting 12 petals representing three habitats within four taxonomic strains shows the gene content of the core genome (center) and strain-specific genes (petals) among 573 genomes. (B) Functional proteins of the core genome of LA and SA PGPB strains with the highest number of genes. The horizontal axis represents various functional proteins, the green portion indicates that the LA PGPB contains a greater number of significant genes compared to the SA PGPB and OA strains, and the yellow portion indicates that the SA PGPB contains a greater number of significant genes compared to the LA PGPB and OA strains, while the black portion indicates only that the LA/SA PGPB have the highest content among the three and there is significant difference among them. The asterisk indicates a statistically significant difference in the number of genes encoding different proteins among the three habitats, as determined by ANOVA, and the greater the number of asterisks, the more significant the difference (*, 0.01< *P* < 0.05; **, 0.001 < *P *< 0.01; ***, 0.0001 < *P *< 0.001; ****, *P* < 0.0001). The bar charts represent the number of functional proteins in statistical tests, and the error bars represent the standard deviations of the number of genes in the core genomes of LA/SA PGPB and OA strains.

To gain a deeper understanding of the genetic underpinnings and biocontrol properties of PGPB across different environments, we conducted a genome analysis of 12 units from four taxa across three habitats, resulting in a pan-genome. Our findings revealed the identification of 59,187 pan-genome gene families, of which only 163 (0.28% of the total) were considered core gene families that were present in all strains and conserved. The remaining 59,024 (99.72% of the total) represented the accessory genome (37,784 [63.83%]) and strain-specific genes (21,240 [35.89%]) (Fig. 2A). Meanwhile, phylogenetic principal-component analysis (phylo-PCA) of the 163 core genes in three habitats showed that the gene abundance in all strains was correlated with different habitat characteristics (Fig. S4) (Adonis test, *P* = 0.001), suggesting that the abundance of these genes may play an important role in adapting to the environment.

Therefore, we randomly selected strains with the closest average phylogenetic distance from each of the three habitats and conducted a comparative analysis of their 163 core gene families (method 3). Analysis of the NR database revealed that LA PGPB strains had higher levels of eight core genes than SA PGPB and OA strains, which are related to environmental adaptation, such as DNA repair, cytochrome P450s, AraC family transcriptional regulators, and motility chemotaxis (Fig. 2B and Fig. S5). In SA PGPB strains, 16 core genes were significantly more abundant than in LA PGPB and OA strains, including genes encoding proteins with helix-turn-helix structures, acylamidases, TetR family transcriptional regulators, and YceI family proteins and genes for spore adaptation to the environment (Fig. 2B and Fig. S5). Moreover, 15 core genes were more abundant in LA or SA PGPB strains than in OA strains, including genes for ABC transport proteins, α/β hydrolases, ArsC, and flavin adenine dinucleotide (FAD)-binding oxidoreductases. In contrast, OA strains had an additional 34 abundant core genes, which may be associated with their broad habitat range, including transglutaminase, aspartic acid hemaldehyde dehydrogenase, and alanine racemate, among others (Fig. S5).

### CAZyme profiling.

**FIG 3:**
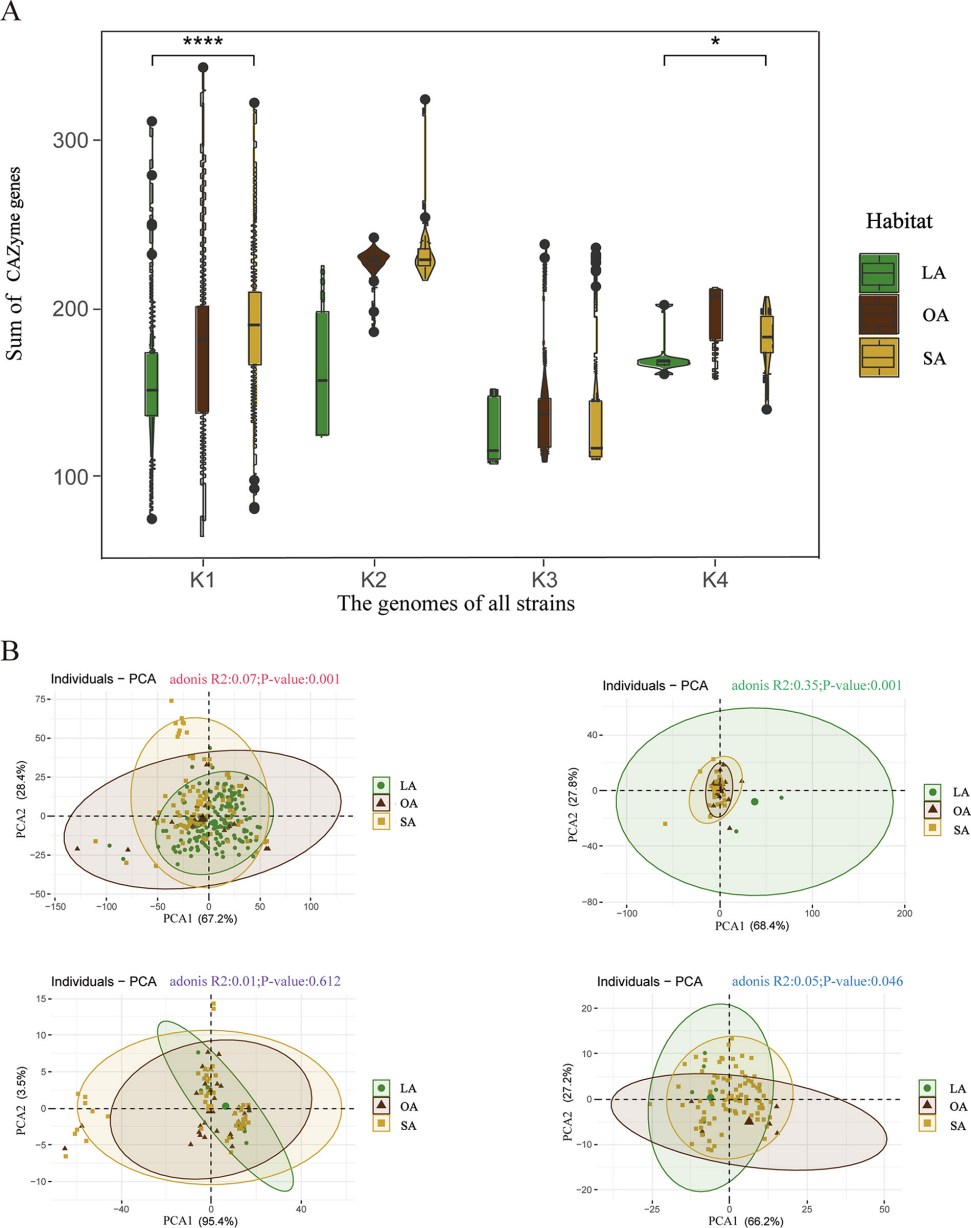
Carbohydrate-active enzymes (CAZymes) in all strains. (A) The total number of carbohydrase enzymes in the genomes of the four taxonomic strains was compared among the three habitats using a violin plot based on the CAZymes data set (*t* test; *, 0.01 < *P *< 0.05; ****, *P* < 0.0001). (B) A total of four phylogenetic principal-component analyses (Adonis test, *P < *0.05) were conducted to compare the total number of carbohydrase enzymes in the genomes of the four taxonomic strains across the three habitats.

To gain a more profound understanding of the potential function of CAZymes in environmental adaptation in each genome, we employed an internal script to determine the number and types of these enzymes in the strain genome to explore the genomic potential of all strains in utilizing carbohydrate enzymes (method 4). Our analysis revealed the presence of numerous genes encoding crucial carbohydrate enzymes in PGPB strains thriving in the SA habitat (Fig. 2B and Fig. S6). Our findings revealed that 59 CAZyme families were present in all strains, including 32 glycoside hydrolases (GHs), 25 glycosyltransferases (GTs), and 2 carbohydrate-binding module (CBM) families. GHs and GTs were the most abundant CAZyme families in all strains. Nevertheless, there were significant variations in the number of genes encoding CAZymes among the strains, with a range of 82 to 417 (Fig. 3A). This indicates that PGPB strains possess significant carbohydrate degradation capabilities, and GHs and GTs may be the pivotal CAZyme genes necessary for these strains’ adaptation and survival in diverse environments.

Further analysis has uncovered significant differences in the total number of CAZyme genes among strains of bacteria growing in different habitats. Specifically, strains growing in the SA habitat showed a significantly higher number of CAZyme genes than those growing in the LA habitat, for both K1 and K4 taxa. In contrast, in the LA habitat, the K4 taxon exhibited the highest average number of CAZyme genes, while in the SA habitat, the K2 taxon had the highest average number of CAZyme genes (*t* test, *P < *0.05) (Fig. 3A). Phylo-PCA analysis further revealed that the CAZyme genes in the K1, K2, and K4 taxa could be separated into two axes, PC1 and PC2. Interestingly, there were significant differences in CAZyme genes among strains from different habitats along these two axes (Adonis test, *P < *0.05) (Fig. 3B). However, in the K3 taxon, which mainly consisted of *Bacillus* strains, there was no significant difference in CAZyme genes among strains growing in LA, SA, PGPB, and OA strains (*t* test, *P < *0.05) (Fig. 3B and Table S2). This suggests that the CAZyme genes of most PGPB are related to the habitat of the strains, whereas the K3 taxon appears to exhibit stable CAZyme levels across different environments.

**FIG 4:**
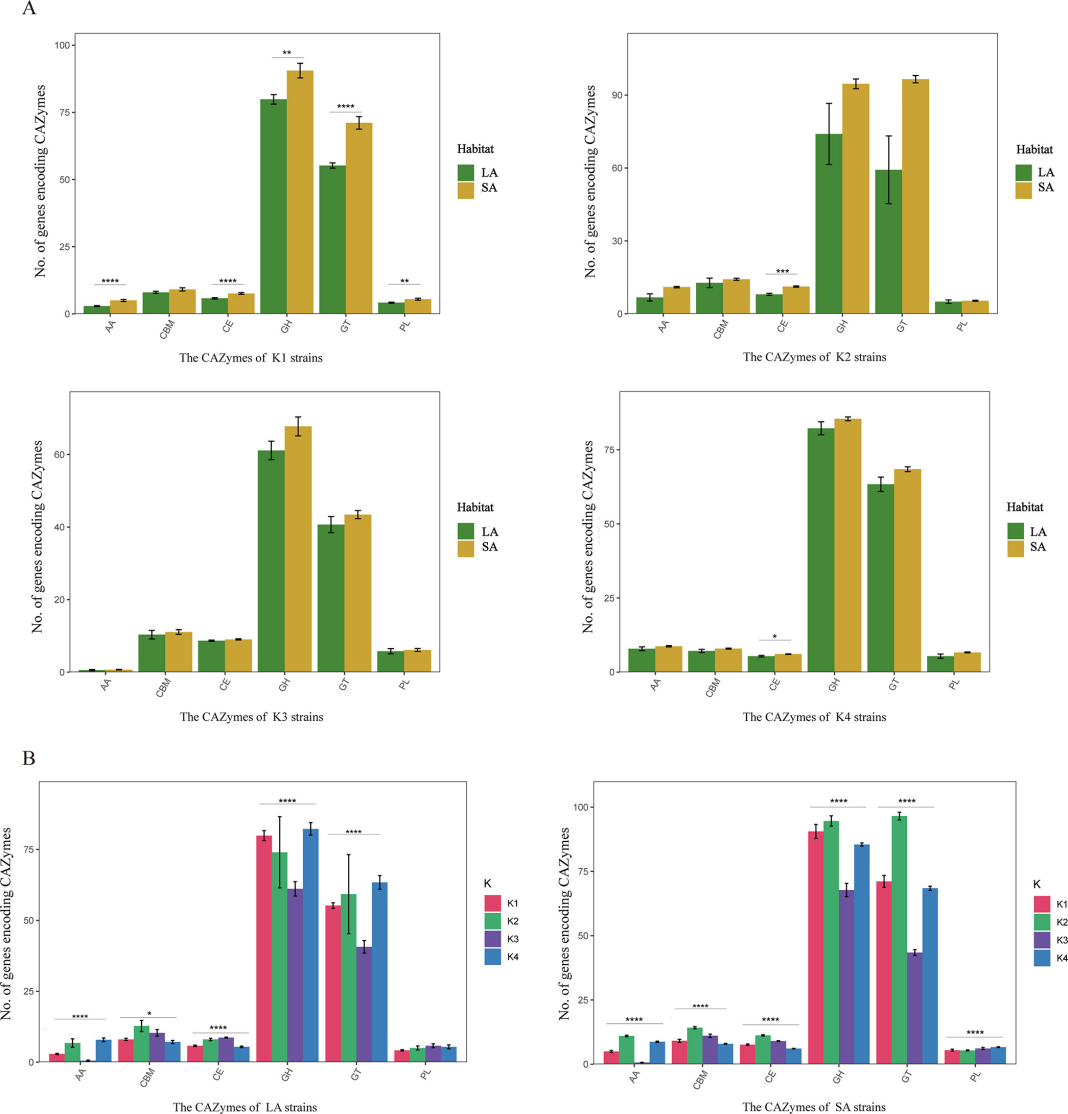
Statistical analysis of auxiliary activities (AAs), carbohydrate-binding modules (CBMs), carbohydrate esterases (CEs), glycoside hydrolases (GHs), glycosyltransferases (GTs), and polysaccharide lyases (PLs). (A) Quantitative comparison of carbohydrate enzymes in PGPB strains from two LA and SA habitats within the four taxa (*t* test; *, 0.01 < *P *< 0.05; **, 0.001 < *P *< 0.01; ***, 0.0001 < *P *< 0.001; ****, *P* < 0.0001). The bar charts represent the number of carbohydrate enzymes in the statistical tests, and the error bars represent the standard deviations of the number of carbohydrate enzymes in the LA/SA habitat strains. (B) Comparison of the number of carbohydrate enzymes in the four taxa of PGPB from LA and SA habitats (Adonis test; *, 0.01 < *P *< 0.05; ****, *P *< 0.0001). The bar charts depict the statistical test results for the number of carbohydrate enzymes, with error bars representing the standard deviations of these enzymes in PGPB strains of the K1/K2/K3/K4 taxa.

To identify specific variations in the types of CAZyme genes among strains in diverse environments, we utilized Rstudio for additional statistical analysis (method 6). Our results showed that strains from LA and SA habitats in the K1 taxon exhibited significant differences in five types of CAZyme genes (i.e., genes for auxiliary activities [AAs], carbohydrate esterases [CEs], GHs, GTs, and polysaccharide lyases [PLs]). In contrast, for the K2 and K4 taxa, only CEs demonstrated significant differences between LA and SA habitats. Notably, we did not observe any significant differences in specific CAZyme genes between different habitats in the K3 taxon (*t* test, *P < *0.05) (Fig. 4A). Further analysis revealed that in the LA habitat, all five types of CAZyme genes (genes for AAs, CEs, GHs, GTs, and CBMs) showed significant differences among the four clusters. In the SA habitat, six types of CAZyme genes in all four clusters exhibited significant differences, with higher CAZyme gene richness than in the LA habitat (*t* test, *P < *0.05) (Fig. 4B). These findings lead us to conclude that variations in the types and richness of CAZyme genes in different habitats may assist microorganisms in adapting to their ecological environments.

### Analysis of the secondary metabolic cluster.

**FIG 5:**
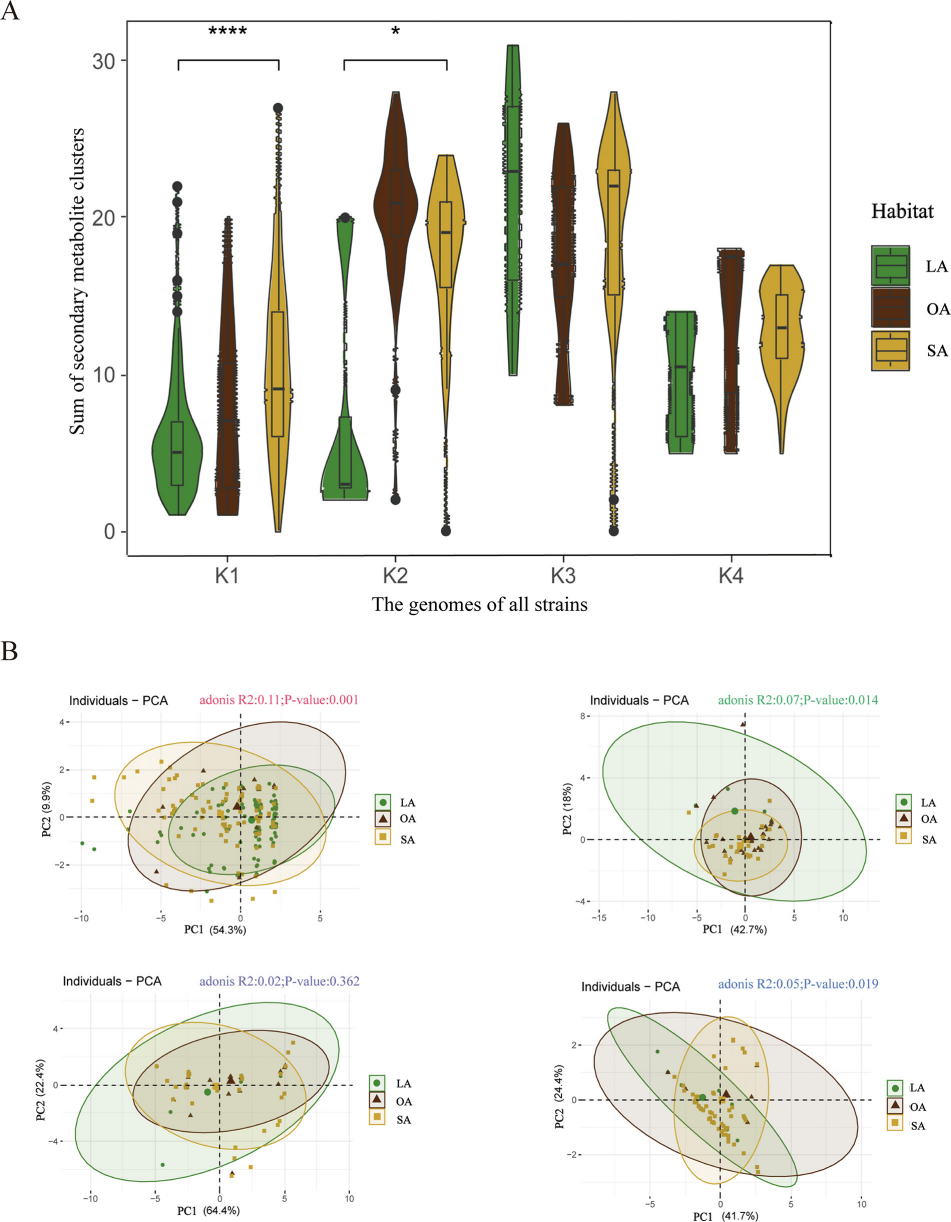
Secondary metabolic clusters in all strains. (A) A violin plot, based on data from antiSMASH, was utilized to compare the total number of biosynthetic gene clusters (BGCs) for secondary metabolites present in the genomes of the four taxonomic strains across the three habitats (*t* test; *, 0.01 < *P *< 0.05; ****, *P* < 0.0001). (B) A total of four phylogenetic principal-component analyses (Adonis test; *P < *0.05) were conducted to compare the total number of secondary metabolite biosynthetic gene clusters in the genomes of the four taxonomic strains across the three habitats.

To evaluate the potential production of antimicrobial compounds, we utilized the antiSMASH software to predict the quantities and types of secondary metabolite clusters present in all strains (method 5). Our findings indicated that PGPB strains contained both NRPS and bacteriocin genes, with various numbers of secondary metabolite clusters observed across different habitats. Specifically, we noted a significant difference in the total number of secondary metabolite clusters among strains of taxa K1 and K2 in LA and SA habitats (Fig. 5A). Furthermore, phylo-PCA highlighted significant differences in the secondary metabolite clusters of K1, K2, and K4 strains across different habitats on the PC1 and PC2 axes (Fig. 5B), suggesting the potential of PGPB secondary metabolite clusters to play a crucial role in environmental adaptation. Although there was no significant difference in secondary metabolite clusters between strains in different habitats within the K3 taxon, they had the highest level of secondary metabolite clusters (Fig. 5A). Notably, the K3 taxon comprised primarily of *Bacillus* strains, supporting the notion that *Bacillus* could serve as a reliable and effective biocontrol agent. Overall, these findings provide deeper insight into the distribution and function of secondary metabolite clusters of PGPB strains across different habitats, offering a theoretical foundation for using PGPB as a biocontrol agent.

**FIG 6:**
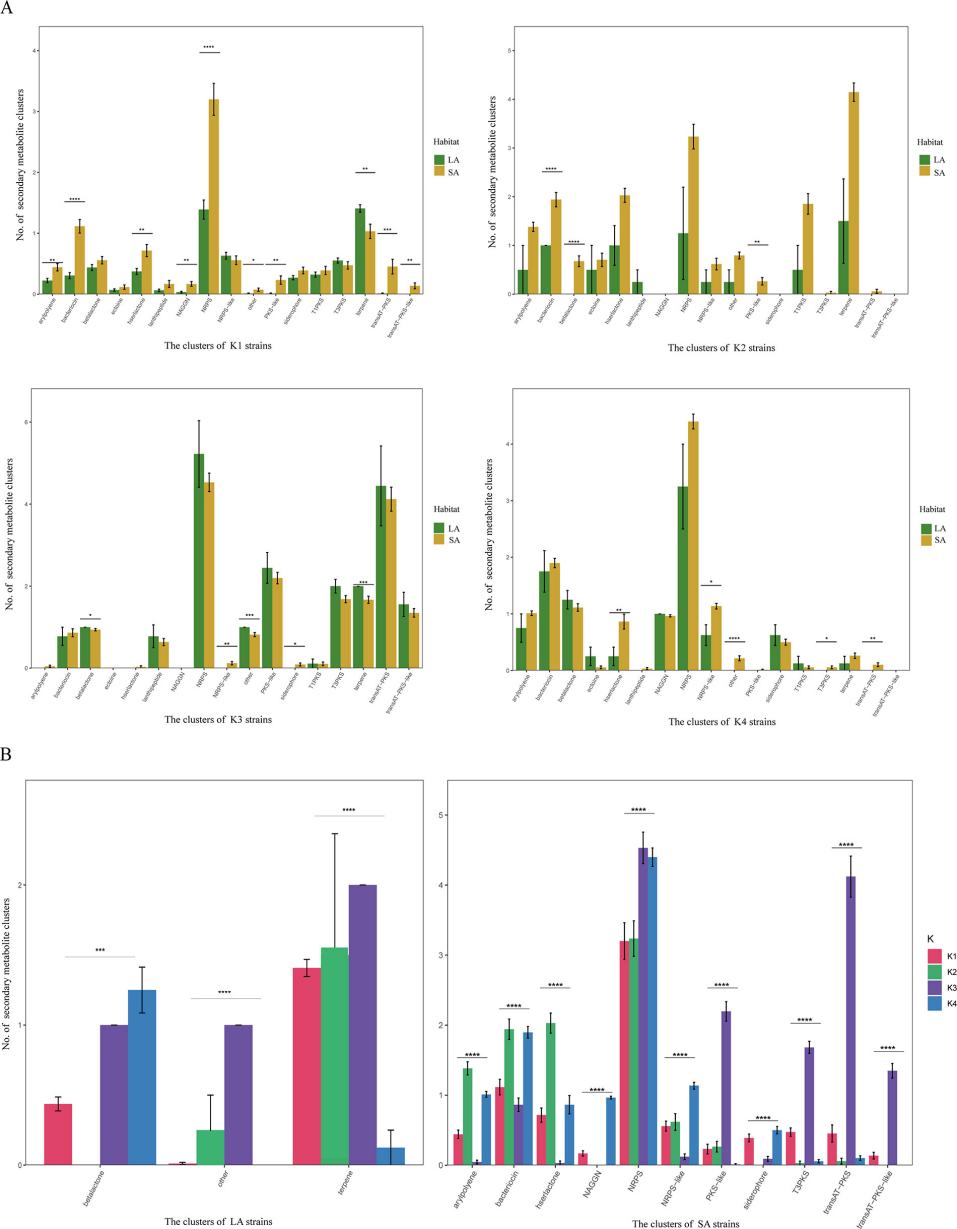
Statistical analysis of top 17 secondary metabolic clusters (*F *> 100). (A) Quantitative comparison of secondary metabolic clusters in PGPB strains from two LA and SA habitats within the four taxa (*t* test; *, 0.01 < *P *< 0.05; **, 0.001 < *P *< 0.01; **,* 0.0001 < *P *< 0.001; ****, *P < *0.0001). Bar charts represent the number of secondary metabolic clusters in statistical tests, and the error bars represent the standard deviations of the number of secondary metabolic clusters in the LA/SA habitat strains. (B) Comparison of the number of secondary metabolic clusters in the four taxa of PGPB from LA and SA habitats (Adonis test; ***, 0.0001 < *P *< 0.001; ****, *P < *0.0001). Bar charts represent the number of secondary metabolic clusters in statistical tests, and the error bars represent the standard deviations of the number of secondary metabolic clusters for PGPB in the K1/K2/K3/K4 taxa.

We conducted a comparative analysis of the 17 most abundant secondary metabolite clusters between LA and SA PGPB strains across all four taxa (Fig. 6A). Our results revealed significant differences in nine secondary metabolite clusters between the LA and SA habitats of taxon K1, including those for several gene clusters associated with the biosynthesis of secondary metabolites such as arylpolyene, bacteriocin, hserlactone, *N*-acetyl glutaminyl glutamine amide (NAGGN), NRPS, polyketide synthase (PKS)-like, terpene production, transAT-PKS, and transAT-PKS-like.

In contrast, taxa K2 and K3 displayed more distinct secondary metabolite clusters. For example, taxon K2 exhibited three different secondary metabolite clusters, including bacteriocin, β-lactone, and PKS-like, which varied between the LA and SA habitats. Similarly, taxon K3 contained four significantly different secondary metabolite clusters, namely, β-lactone, NRPS-like, siderophore, and terpene production. Finally, taxon K4 displayed four significantly different secondary metabolite clusters, specifically hserlactone, NRPS-like, type I, II, and III polyketide synthase (T3PKS), and transAT-PKS. Our findings indicated that the LA habitat demonstrated a significant enrichment in two secondary metabolite clusters, β-lactone and terpene production, while the SA habitat was notably enriched in 11 secondary metabolite clusters, including arylpolyene, bacteriocin, hserlactone, NAGGN, NRPS, NRPS-like, PKS-like, siderophore, T3PKS, transAT-PKS, and transAT-PKS-like (Student’s *t* test, *P < *0.05) (Fig. 6A and B).

Further analysis revealed that taxa K1, K2, and K4 had a significantly higher number of enriched secondary metabolite clusters in the SA habitat than the LA habitat. On the other hand, the members of taxon K3 exhibited more enriched secondary metabolite clusters in the LA habitat. Furthermore, taxon K3 showed the highest number of secondary metabolite clusters in both LA and SA habitats, highlighting its potential as an effective biocontrol agent in both habitats. Notably, taxon K3 mainly comprised *Bacillus* PGPB strains.

## DISCUSSION

The microorganisms in a habitat are selected by environmental factors, which results in distinct microbial genome variations among different habitats (16, 17). PGPB strains have been observed in various habitats, as documented in the NCBI database (18). PGPB isolated from LA and SA habitats are crucial components of agroecosystems and contribute significantly to healthy crops (19). In this study, a comparative functional genome analysis was conducted on 573 strains to gain insights into the genomic features of PGPB.

Bacteria inhabiting complex ecosystems typically have larger genomes, often containing functionally redundant genes (20, 21). A larger number of function-related genes may aid bacteria in adapting to complex environments and improving their biocontrol characteristics, leading to enhanced adaptability, survival, and growth (22). Our study found that SA PGPB strains had significantly larger average genome sizes than LA PGPB strains, which may be attributed to the intricacies of the soil environment. Soil structure, physicochemical factors, and temperature fluctuations can impact bacterial growth more extensively than above-ground conditions (23, 24).

A prior study recognized the significant role of core genes in controlling essential bacterial functions (25), and many plant-related functions were found to be conserved across diverse bacterial taxa (4). Genomic functional analysis of PGPB strains from the LA habitat revealed significant abundance of core genes that were related to DNA repair and contributed to exceptional resistance and response to environmental stress (26). These strains were also enriched in cytochrome P450 genes, which could suppress leaf senescence, prolong leaf life span, and boost plant adaptability to the environment (27). Additionally, these genes were involved in the biosynthesis and metabolism of several plant hormones, such as salicylic acid, jasmonic acid, ethylene, and abscisic acid (28). Furthermore, AraC family transcriptional regulators were identified as key to modulation and management of cellular metabolism, thereby allowing the bacteria to survive under various environmental conditions (29). Bacterial chemotaxis could aid in responding to chemical concentration gradients of substances and enabling the bacteria to prefer beneficial stimuli and avoid harmful ones (30) (Fig. 2A and Fig. S4).

Our analysis revealed that PGPB inhabiting the SA habitat possess a distinct gene repertoire compared to those in the LA and OA habitats. Specifically, they exhibited enrichment in genes related to carbohydrate and amino acid metabolism, suggesting their ability to utilize these nutrients. Interestingly, we also observed an abundance of genes encoding helix-turn-helix domains, which can help alleviate inhibition of virulence gene expression and promote biofilm formation (31). Additionally, the SA PGPB strains were found to have a high number of acylamidase genes, which may allow indirect hydrolysis of immunoregulatory peptidoglycans (32), and transcription regulator genes of the TetR and YceI families, which aid in stress tolerance (33–35). Finally, the abundance of sporulation genes observed in these strains may contribute to their ability to withstand environmental stressors (36).

Compared to OA strains, the LA and SA PGPB strains were found to contain many genes encoding ABC transporter proteins, α/β hydrolase, and ArsC reductases, which might contribute to the degradation of environmental pollutants and improve environmental adaptability of the strains (37). Moreover, the PGPB strains were also found to be rich in FAD-binding oxidoreductase, which might protect the strains against hypoxic and oxidative stress (38). Additionally, these strains have the ability to produce exopolysaccharides and antibiotics that help PGPB colonize plants and protect the host from pathogens (39, 40).

CAZymes and secondary metabolite clusters are crucial factors in determining whether bacteria exhibit biocontrol functions (7, 41). The results of this study demonstrate that PGPB generally contain a large quantity of CAZymes, which enhance their ability to colonize plants (42). Among the LA PGPB strains, Pseudomonas strains exhibited a higher abundance of CAZymes than did K1, K2, and K3 PGPB strains, indicating that this genus could be developed into an ideal interfoliar biocontrol agent (43). Conversely, among the SA PGPB strains, *Burkholderia* strains possessed a higher concentration of genes encoding carbohydrate metabolism enzymes, suggesting that they have diverse mechanisms for carbohydrate utilization (44).

GHs are the key resources for bacteria to produce degraded polysaccharides and promoted their growth (2, 45). GTs can synthesize extracellular polysaccharides, which were found to be conducive to the formation of biofilms and resistance to environmental pressures (39, 46, 47). CBMs are noncatalytic domains, which can be folded into a specific three-dimensional spatial structure and have the function of binding carbohydrate (48). PLs were required for plant growth and leaf senescence, which had no differ significance in four taxa (49). The genus *Burkholderia* has demonstrated significant differences in CEs (50). Pseudomonas belongs to the lipase-producing bacteria and has been found only in the endosymbiotic flora, while Pseudomonas also shows a greater abundance inside the cell (51). Follow justly, *Pseudomonas* exhibited a significant difference in the number of CEs between LA and SA habitats.

The antiSMASH program is commonly used to predict secondary metabolite gene clusters that may confer the ability to produce antimicrobial compounds (52). Bacteria may have a wider range of functional diversity if they possess a larger number of genes in their genomes (53). The greater the number of secondary metabolic gene clusters in bacteria, the stronger their ability to perform biological defense (54). *Bacillus* strains are dominant biocontrol agents in the market (55, 56). The majority of *Bacillus* strains of PGPB possess more abundant secondary metabolic clusters than other taxonomic groups in the LA and SA habitats, with the LA habitat accounting for more. It has been demonstrated that Bacillus subtilis applied in a slow-release manner by foliar spraying or petiole inoculation significantly improves the effectiveness of antipathogenic bacteria (57). All PGPB strains were found to have NRPS, which can synthesize a series of low-molecular-weight peptide-like secondary metabolites with medicinal value through the nonribosomal pathway with NRPs (58). These secondary metabolites include antibiotics, iron carriers, toxins, and pigments and have antibacterial, antifungal, anticancer, antiviral, and immunosuppressive activities (59). Bacteriocins, a class of peptide toxins with antibacterial activity that act as probiotics (60), are also present in all PGPB strains.

In conclusion, we investigated the genetic basis and biocontrol characteristics of 478 strains across different habitats. Our findings revealed that LA PGPB strains possess genes related to environmental adaptation, such as genes for DNA repair, cytochrome P450s, AraC transcriptional regulators, and motor chemotaxis, while SA PGPB strains possess genes related to helix-turn-helix domain-containing proteins, amidases, TetR transcriptional regulators, YceI family proteins, and sporulation. Additionally, LA PGPB strains were enriched in genes related to hormone production, while SA PGPB strains were enriched in genes related to carbohydrate and antibiotic metabolism. These genes contributed to the domination of their complex environment and enhanced their biocontrol efficacy. The genera *Bacillus* and *Paenibacillus*, found in the LA and SA habitats, produced a higher number of secondary metabolite clusters, making them suitable for both leaf and soil environments. *Burkholderia* spp. produced numerous secondary metabolite clusters in the OA habitat, indicating their significant presence in nonplant environments and their potential as promising PGPB. Developing agrochemicals specific to different habitats would help improve the growth promotion and disease prevention effects of PGPB.

## MATERIALS AND METHODS

### Compilation of data sets for constructing a high-quality genome.

In this study, relevant literature on plant promotion and/or biocontrol was searched for using Web of Science (https://www.webofscience.com/wos/alldb/basic-search) as the primary source, followed by manual proofreading. The corresponding whole-genome sequence of the strains was then downloaded from the NCBI website (https://www.ncbi.nlm.nih.gov/). Additionally, a previously established method for constructing a high-quality genome set was referenced (4, 61) (see Table S1 in the supplemental material). Briefly, the average nucleotide identity (ANI) and coverage of each strain were calculated using PYANI. Two genomes were considered functionally redundant if their ANI was at least 99.9% and their coverage was over 95%, in which case one was randomly removed. CheckM (62) was used to evaluate genome completeness (63) and contamination (64), with only genomes that were at least 95% complete and had no more than 5% contamination being used. In addition, the digital DNA-DNA hybridization (dDDH) value was calculated for 573 bacterial strains using the Genome BLAST Distance Phylogeny (GBDP) method (https://ggdc.dsmz.de/ggdc.php) (65). Finally, metadata collection was conducted to obtain information on isolation, genome size, and compartment from NCBI, IMG (https://img.jgi.doe.gov/), and manually collated references.

We compiled a data set of 573 bacterial strains with plant growth-promoting and/or disease-suppressing activity from the literature (Table S1). These strains originated from 36 countries and regions on all seven continents, with 195 strains isolated from plant leaves as LA PGPB, 283 isolated from soil as SA PGPB, and 95 isolated from other habitats unrelated to plants as OA PGPB (Fig. 1A and Fig. S1). Our collection included strains from midfield soils in Hokkaido, Japan (66), bean leaf strains from tropical forests in Brazil with antimicrobial activity (67), and many strains previously characterized as promising biocontrol agents.

### Phylogenetic analysis.

The amino acid sequences corresponding to 14 single-copy core genes were extracted from the OrthoFinder v2.2.7 output files (68). A maximum likelihood phylogenetic tree was constructed based on the single-copy core genome using FastTree v2.1.9 (69) and visualized with iTOL (https://itol.embl.de/) (70). We converted the phylogenetic tree into a distance matrix using the cophenetic function in the ape package in R. Next, we performed *k*-medoid clustering analysis and maximum profile coefficient determination on the 573 strains, which were clustered into one taxon considered to have a similar genetic background (4). Subsequently, we applied the PAM algorithm from the R package fpc to cluster the 573 genomes into four groups using *k*-medoid clustering. The *k*-medoid algorithm clusters a data set of *n* objects into *k* predefined clusters. To determine the optimal *k* value, we compared silhouette coefficients for *k* values ranging from 1 to 20. We selected a *k* value of 4, as it resulted in the highest average silhouette coefficient (0.44).

We used the R package pvclust for hierarchical clustering to demonstrate that all strains originating from the same habitat exhibit convergent evolution in their adaptation to the environment and their capacity to function as biocontrol agents (71–73).

### Core genome analysis.

We randomly selected 95 genomes from a pool of 195 LA PGPB genomes, 95 genomes from a pool of 283 SA PGPB genomes, and 95 genomes from all OA strain genomes. The mean phylogenetic distances between the LA/SA PGPB genomes and the OA strain genomes were calculated, and the genome with the smallest deviation from the average phylogenetic distance of OA strain genomes was selected. The same method was used to calculate the phylogenetic distances between the 195 LA PGPB genomes and the 195 SA PGPB genomes, and the genome with minimal deviation was selected. Finally, these genomes were used for comparative analysis of the core genome function (4).

Orthologous groups of protein families of core genome were delimited using OrthoFinder v2.2.7 software with the Diamond method (68, 74). The resulting output files (Orthogroup_Sequences folder) were used to extract core genome families (genes shared among all strains) and strain-specific genes (genes found only in the same taxa, the same habitat strains) (Fig. 2A).

### Carbohydrate enzyme analysis.

Carbohydrase distribution in 573 strains was predicted using the CAZy database. Accession numbers of known carbohydrases were obtained from CAZy, and corresponding genome sequences were obtained from NCBI Batch Entrez (https://www.ncbi.nlm.nih.gov/sites/batchentrez). Data were formatted using Diamond and sequences with more than 40% identity were kept. Finally, the accession numbers and CAZy database carbohydrase names were compared to match them with each strain for predicting the presence of carbohydrases.

### Secondary metabolic cluster analysis.

To predict secondary metabolic clusters in the 573 strains, we utilized the antiSMASH localization database. Initially, we created an environment to install and activate the antiSMASH software, downloaded the database, and tested the genome of one strain to obtain the web version of antiSMASH (https://antismash.secondarymetabolites.org/#!/start) with consistent results. Subsequently, we processed the data in batches to obtain the number of secondary metabolic clusters for all 573 strains by collecting and analyzing the corresponding result files.

### Statistical analysis.

Extensive use of Rstudio (https://www.rstudio.com/) and the R package ggplot2 were employed for data visualization. Latitude and longitude coordinates were obtained in batches, and hierarchical clustering was used to confirm the distribution criteria of habitats. *k*-Medoid clustering was used to analyze the phylogenetic relationships of similar taxa. Core gene families were displayed using Venn diagrams, and the number of carbohydrases and secondary metabolic clusters were shown using violin plots. To analyze the functional abundance of each taxonomic hierarchy, dimensionality reduction analysis was performed using principal-component analysis (PCA) (62), and statistical analysis of functional differences among all taxa was conducted using analysis of multiple differences among all taxa (ANOVA) and *t* test.

## References

[1] Ankati S, Podile AR. 2019. Metabolites in the root exudates of groundnut change during interaction with plant growth promoting rhizobacteria in a strain-specific manner. J Plant Physiol 243:153057. doi:10.1016/j.jplph.2019.153057. 31675630

[2] Johnson DR, Goldschmidt F, Lilja EE, Ackermann M. 2012. Metabolic specialization and the assembly of microbial communities. ISME J 6:1985–1991. doi:10.1038/ismej.2012.46. 22592822 PMC3475376

[3] Vejan P, Abdullah R, Khadiran T, Ismail S, Nasrulhaq Boyce A. 2016. Role of plant growth promoting rhizobacteria in agricultural sustainability—a review. Molecules 21:573. doi:10.3390/molecules21050573. 27136521 PMC6273255

[4] Levy A, Salas Gonzalez I, Mittelviefhaus M, Clingenpeel S, Herrera Paredes S, Miao J, Wang K, Devescovi G, Stillman K, Monteiro F, Rangel Alvarez B, Lundberg DS, Lu TY, Lebeis S, Jin Z, McDonald M, Klein AP, Feltcher ME, Rio TG, Grant SR, Doty SL, Ley RE, Zhao B, Venturi V, Pelletier DA, Vorholt JA, Tringe SG, Woyke T, Dangl JL. 2017. Genomic features of bacterial adaptation to plants. Nat Genet 50:138–150. doi:10.1038/s41588-017-0012-9. 29255260 PMC5957079

[5] Gielen S, Aerts R, Seels B. 2004. Biocontrol agents of Botrytis cinerea tested in climate chambers by making artificial infection on tomato leafs. Commun Agric Appl Biol Sci 69:631–639. 15756850

[6] Rojas-Rojas FU, Salazar-Gómez A, Vargas-Díaz ME, Vásquez-Murrieta MS, Hirsch AM, De Mot R, Ghequire MGK, Ibarra JA, Estrada-de Los Santos P. 2018. Broad-spectrum antimicrobial activity by Burkholderia cenocepacia TAtl-371, a strain isolated from the tomato rhizosphere. Microbiology (Reading) 164:1072–1086. doi:10.1099/mic.0.000675. 29906254

[7] Zaid DS, Cai S, Hu C, Li Z, Li Y. 2022. Comparative genome analysis reveals phylogenetic identity of Bacillus velezensis HNA3 and genomic insights into its plant growth promotion and biocontrol effects. Microbiol Spectr 10:e02169-21. doi:10.1128/spectrum.02169-21. 35107331 PMC8809340

[8] Malik SS, Sudalaimuthuasari N, Kundu B, AlMaskari RS, Mundra S. 2022. Contrasting genome patterns of two Pseudomonas strains isolated from the date palm rhizosphere to assess survival in a hot arid environment. World J Microbiol Biotechnol 38:207. doi:10.1007/s11274-022-03392-4. 36008694

[9] Battaglia E, Benoit I, van den Brink J, Wiebenga A, Coutinho PM, Henrissat B, de Vries RP. 2011. Carbohydrate-active enzymes from the zygomycete fungus Rhizopus oryzae: a highly specialized approach to carbohydrate degradation depicted at genome level. BMC Genomics 12:38. doi:10.1186/1471-2164-12-38. 21241472 PMC3032700

[10] Liu K, Newman M, McInroy JA, Hu CH, Kloepper JW. 2017. Selection and assessment of plant growth-promoting rhizobacteria for biological control of multiple plant diseases. Phytopathology 107:928–936. doi:10.1094/PHYTO-02-17-0051-R. 28440700

[11] Esmaeel Q, Pupin M, Jacques P, Leclère V. 2018. Nonribosomal peptides and polyketides of Burkholderia: new compounds potentially implicated in biocontrol and pharmaceuticals. Environ Sci Pollut Res Int 25:29794–29807. doi:10.1007/s11356-017-9166-3. 28547376

[12] Zhao Y, Xie X, Li J, Shi Y, Chai A, Fan T, Li B, Li L. 2022. Comparative genomics insights into a novel biocontrol agent Paenibacillus peoriae strain ZF390 against bacterial soft rot. Biology (Basel) 11:1172. doi:10.3390/biology11081172. 36009799 PMC9404902

[13] Dias GM, de Sousa Pires A, Grilo VS, Castro MR, de Figueiredo Vilela L, Neves BC. 2019. Comparative genomics of Paraburkholderia kururiensis and its potential in bioremediation, biofertilization, and biocontrol of plant pathogens. Microbiologyopen 8:e00801. doi:10.1002/mbo3.801. 30811107 PMC6692535

[14] Nelkner J, Tejerizo GT, Hassa J, Lin TW, Witte J, Verwaaijen B, Winkler A, Bunk B, Spröer C, Overmann J, Grosch R, Pühler A, Schlüter AA. 2019. Genetic potential of the biocontrol agent Pseudomonas brassicacearum (formerly P. trivialis) 3Re2-7 unraveled by genome sequencing and mining, comparative genomics and transcriptomics. Genes (Basel) 10:601. doi:10.3390/genes10080601. 31405015 PMC6722718

[15] Zhang N, Yang D, Kendall JR, Borriss R, Druzhinina IS, Kubicek CP, Shen Q, Zhang R. 2016. Comparative genomic analysis of Bacillus amyloliquefaciens and Bacillus subtilis reveals evolutional traits for adaptation to plant-associated habitats. Front Microbiol 7:2039. doi:10.3389/fmicb.2016.02039. 28066362 PMC5169363

[16] Fira D, Dimkić I, Berić T, Lozo J, Stanković S. 2018. Biological control of plant pathogens by Bacillus species. J Biotechnol 285:44–55. doi:10.1016/j.jbiotec.2018.07.044. 30172784

[17] Chase AB, Arevalo P, Brodie EL, Polz MF, Karaoz U, Martiny JBH. 2019. Maintenance of sympatric and allopatric populations in free-living terrestrial bacteria. mBio 10:e02361-19. doi:10.1128/mBio.02361-19. 31662456 PMC6819660

[18] O’Leary NA, Wright MW, Brister JR, Ciufo S, Haddad D, McVeigh R, Rajput B, Robbertse B, Smith-White B, Ako-Adjei D, Astashyn A, Badretdin A, Bao Y, Blinkova O, Brover V, Chetvernin V, Choi J, Cox E, Ermolaeva O, Farrell CM, Goldfarb T, Gupta T, Haft D, Hatcher E, Hlavina W, Joardar VS, Kodali VK, Li W, Maglott D, Masterson P, McGarvey KM, Murphy MR, O'Neill K, Pujar S, Rangwala SH, Rausch D, Riddick LD, Schoch C, Shkeda A, Storz SS, Sun H, Thibaud-Nissen F, Tolstoy I, Tully RE, Vatsan AR, Wallin C, Webb D, Wu W, Landrum MJ, Kimchi A, et al. 2016. Reference sequence (RefSeq) database at NCBI: current status, taxonomic expansion, and functional annotation. Nucleic Acids Res 44:D733–D745. doi:10.1093/nar/gkv1189. 26553804 PMC4702849

[19] Parizadeh M, Mimee B, Kembel SW. 2020. Neonicotinoid seed treatments have significant non-target effects on phyllosphere and soil bacterial communities. Front Microbiol 11:619827. doi:10.3389/fmicb.2020.619827. 33584586 PMC7873852

[20] Tripp HJ, Bench SR, Turk KA, Foster RA, Desany BA, Niazi F, Affourtit JP, Zehr JP. 2010. Metabolic streamlining in an open-ocean nitrogen-fixing cyanobacterium. Nature 464:90–94. doi:10.1038/nature08786. 20173737

[21] Qin W, Zheng Y, Zhao F, Wang Y, Urakawa H, Martens-Habbena W, Liu H, Huang X, Zhang X, Nakagawa T, Mende DR, Bollmann A, Wang B, Zhang Y, Amin SA, Nielsen JL, Mori K, Takahashi R, Armbrust EV, Winkler MH, DeLong EF, Li M, Lee PH, Zhou J, Zhang C, Zhang T, Stahl DA, Ingalls AE. 2020. Alternative strategies of nutrient acquisition and energy conservation map to the biogeography of marine ammonia-oxidizing archaea. ISME J 14:2595–2609. doi:10.1038/s41396-020-0710-7. 32636492 PMC7490402

[22] Stefanovic E, Fitzgerald G, McAuliffe O. 2017. Advances in the genomics and metabolomics of dairy lactobacilli: a review. Food Microbiol 61:33–49. doi:10.1016/j.fm.2016.08.009. 27697167

[23] Davis EL, Hager HA, Gedalof Z. 2018. Soil properties as constraints to seedling regeneration beyond alpine treelines in the Canadian Rocky Mountains. Arct Antarct and Alp Res 50:e1415625. doi:10.1080/15230430.2017.1415625.

[24] Briones MJI. 2018. The serendipitous value of soil fauna in ecosystem functioning: the unexplained explained. Front Environ Sci 6:149. doi:10.3389/fenvs.2018.00149.

[25] Yin Z, Liu X, Qian C, Sun L, Pang S, Liu J, Li W, Huang W, Cui S, Zhang C, Song W, Wang D, Xie Z. 2022. Pan-genome analysis of Delftia tsuruhatensis reveals important traits concerning the genetic diversity, pathogenicity, and biotechnological properties of the species. Microbiol Spectr 10:e02072-21. doi:10.1128/spectrum.02072-21. PMC9045143 35230132

[26] Cowan A, Skrede I, Moody SC. 2022. Cytochrome P450 complement may contribute to niche adaptation in serpula wood-decay fungi. J Fungi 8:283. doi:10.3390/jof8030283. PMC8949155 35330285

[27] Jiang L, Yoshida T, Stiegert S, Jing Y, Alseekh S, Lenhard M, Pérez-Alfocea F, Fernie AR. 2021. Multi-omics approach reveals the contribution of KLU to leaf longevity and drought tolerance. Plant Physiol 185:352–368. doi:10.1093/plphys/kiaa034. 33721894 PMC8133585

[28] Narusaka Y, Narusaka M, Seki M, Umezawa T, Ishida J, Nakajima M, Enju A, Shinozaki K. 2004. Crosstalk in the responses to abiotic and biotic stresses in Arabidopsis: analysis of gene expression in cytochrome P450 gene superfamily by cDNA microarray. Plant Mol Biol 55:327–342. doi:10.1007/s11103-004-0685-1. 15604685

[29] Kotecka K, Kawalek A, Kobylecki K, Bartosik AA. 2021. The AraC-type transcriptional regulator GliR (PA3027) activates genes of glycerolipid metabolism in Pseudomonas aeruginosa. Int J Mol Sci 22:5066. doi:10.3390/ijms22105066. 34064685 PMC8151288

[30] Adadevoh JS, Triolo S, Ramsburg CA, Ford RM. 2016. Chemotaxis increases the residence time of bacteria in granular media containing distributed contaminant sources. Environ Sci Technol 50:181–187. doi:10.1021/acs.est.5b03956. 26605857

[31] De Silva RS, Kovacikova G, Lin W, Taylor RK, Skorupski K, Kull FJ. 2007. Crystal structure of the Vibrio cholerae quorum-sensing regulatory protein HapR. J Bacteriol 189:5683–5691. doi:10.1128/JB.01807-06. 17526705 PMC1951804

[32] Park BJ, Yoon YB, Park SC, Lee DH, Shin C, Kwak HJ, Kim JW, Cho SJ. 2022. Peptidoglycan recognition proteins from the earthworm, Eisenia andrei: differential inducibility and tissue-specific expression. Dev Comp Immunol 135:104483. doi:10.1016/j.dci.2022.104483. 35760219

[33] Ramos JL, Martínez-Bueno M, Molina-Henares AJ, Terán W, Watanabe K, Zhang X, Gallegos MT, Brennan R, Tobes R. 2005. The TetR family of transcriptional repressors. Microbiol Mol Biol Rev 69:326–356. doi:10.1128/MMBR.69.2.326-356.2005. 15944459 PMC1197418

[34] Weber A, Kögl SA, Jung K. 2006. Time-dependent proteome alterations under osmotic stress during aerobic and anaerobic growth in Escherichia coli. J Bacteriol 188:7165–7175. doi:10.1128/JB.00508-06. 17015655 PMC1636219

[35] Cox DE, Dyer S, Weir R, Cheseto X, Sturrock M, Coyne D, Torto B, Maule AG, Dalzell JJ. 2019. ABC transporter genes ABC-C6 and ABC-G33 alter plant-microbe-parasite interactions in the rhizosphere. Sci Rep 9:19899. doi:10.1038/s41598-019-56493-w. 31882903 PMC6934816

[36] Lamba S, Muthappa DM, Fanning S, Scannell AGM. 2022. Sporulation and biofilms as survival mechanisms of Bacillus species in low-moisture food production environments. Foodborne Pathog Dis 19:448–462. doi:10.1089/fpd.2022.0006. 35819266

[37] Talwar C, Nagar S, Kumar R, Scaria J, Lal R, Negi RK. 2020. Defining the environmental adaptations of genus Devosia: insights into its expansive short peptide transport system and positively selected genes. Sci Rep 10:1151. doi:10.1038/s41598-020-58163-8. 31980727 PMC6981132

[38] Harold LK, Antoney J, Ahmed FH, Hards K, Carr PD, Rapson T, Greening C, Jackson CJ, Cook GM. 2019. FAD-sequestering proteins protect mycobacteria against hypoxic and oxidative stress. J Biol Chem 294:2903–2912. doi:10.1074/jbc.RA118.006237. 30567740 PMC6393599

[39] Zhu F, Zhang H, Wu H. 2015. Glycosyltransferase-mediated sweet modification in oral streptococci. J Dent Res 94:659–665. doi:10.1177/0022034515574865. 25755271 PMC4502785

[40] Ramakrishna W, Yadav R, Li KF. 2019. Plant growth promoting bacteria in agriculture: two sides of a coin. Appl Soil Ecol 138:10–18. doi:10.1016/j.apsoil.2019.02.019.

[41] Du Y, Ma J, Yin Z, Liu K, Yao G, Xu W, Fan L, Du B, Ding Y, Wang C. 2019. Comparative genomic analysis of Bacillus paralicheniformis MDJK30 with its closely related species reveals an evolutionary relationship between B. paralicheniformis and B. licheniformis. BMC Genomics 20:283. doi:10.1186/s12864-019-5646-9. 30975079 PMC6458615

[42] Bhattacharyya C, Bakshi U, Mallick I, Mukherji S, Bera B, Ghosh A. 2017. Genome-guided insights into the plant growth promotion capabilities of the physiologically versatile Bacillus aryabhattai strain AB211. Front Microbiol 8:411. doi:10.3389/fmicb.2017.00411. 28377746 PMC5359284

[43] Sharma M, Mallubhotla S. 2022. Diversity, antimicrobial activity, and antibiotic susceptibility pattern of endophytic bacteria sourced from Cordia dichotoma L. Front Microbiol 13:879386. doi:10.3389/fmicb.2022.879386. 35633730 PMC9136406

[44] Chen WM, Prell J, James EK, Sheu DS, Sheu SY. 2012. Effect of phosphoglycerate mutase and fructose 1,6-bisphosphatase deficiency on symbiotic Burkholderia phymatum. Microbiology (Reading) 158:1127–1136. doi:10.1099/mic.0.055095-0. 22282515

[45] Juturu V, Wu JC. 2014. Microbial cellulases: engineering, production and applications. Renew Sustain Energy Rev 33:188–203. doi:10.1016/j.rser.2014.01.077.

[46] Mohnike L, Rekhter D, Huang W, Feussner K, Tian H, Herrfurth C, Zhang Y, Feussner I. 2021. The glycosyltransferase UGT76B1 modulates N-hydroxy-pipecolic acid homeostasis and plant immunity. Plant Cell 33:735–749. doi:10.1093/plcell/koaa045. 33955489 PMC8136917

[47] Nauom S, da Silva Neto BR, Ribeiro MS, Pedersoli WR, Ulhoa CJ, Silva RN, Monteiro VN. 2019. Biochemical and molecular study of Trichoderma harzianum enriched secretome protein profiles using lectin affinity chromatography. Appl Biochem Biotechnol 187:1–13. doi:10.1007/s12010-018-2795-2. 29869746

[48] Xiao B, Sanders MJ, Carmena D, Bright NJ, Haire LF, Underwood E, Patel BR, Heath RB, Walker PA, Hallen S, Giordanetto F, Martin SR, Carling D, Gamblin SJ. 2013. Structural basis of AMPK regulation by small molecule activators. Nat Commun 4:3017. doi:10.1038/ncomms4017. 24352254 PMC3905731

[49] Leng YJ, Yang YL, Ren DY, Huang LC, Dai LP, Wang YQ, Chen L, Tu ZJ, Gao YH, Li XY, Zhu L, Hu J, Zhang GH, Gao ZY, Guo LB, Kong ZS, Lin YJ, Qian Q, Zeng DL. 2017. A rice pectate lyase-like gene is required for plant growth and leaf senescence. Plant Physiol 174:1151–1166. doi:10.1104/pp.16.01625. 28455404 PMC5462006

[50] Uroz S, Courty PE, Pierrat JC, Peter M, Buee M, Turpault MP, Garbaye J, Frey-Klett P. 2013. Functional profiling and distribution of the forest soil bacterial communities along the soil mycorrhizosphere continuum. Microb Ecol 66:404–415. doi:10.1007/s00248-013-0199-y. 23455431

[51] Yang Q, Cahn JKB, Piel J, Song YF, Zhang W, Lin HW. 2022. Marine sponge endosymbionts: structural and functional specificity of the microbiome within Euryspongia arenaria cells. Microbiol Spectr 10:e02296-21. doi:10.1128/spectrum.02296-21. 35499324 PMC9241883

[52] Blin K, Shaw S, Steinke K, Villebro R, Ziemert N, Lee SY, Medema MH, Weber T. 2019. antiSMASH 5.0: updates to the secondary metabolite genome mining pipeline. Nucleic Acids Res 47:W81–W87. doi:10.1093/nar/gkz310. 31032519 PMC6602434

[53] Lawrence JG, Hendrickson H. 2005. Genome evolution in bacteria: order beneath chaos. Curr Opin Microbiol 8:572–578. doi:10.1016/j.mib.2005.08.005. 16122972

[54] Dimkic I, Janakiev T, Petrovic M, Degrassi G, Fira D. 2022. Plant-associated Bacillus and Pseudomonas antimicrobial activities in plant disease suppression via biological control mechanisms—a review. Physiol Mol Plant Pathol 117:101754. doi:10.1016/j.pmpp.2021.101754.

[55] Cai XC, Liu CH, Wang BT, Xue YR. 2017. Genomic and metabolic traits endow Bacillus velezensis CC09 with a potential biocontrol agent in control of wheat powdery mildew disease. Microbiol Res 196:89–94. doi:10.1016/j.micres.2016.12.007. 28164794

[56] Zhang N, Yang D, Wang D, Miao Y, Shao J, Zhou X, Xu Z, Li Q, Feng H, Li S, Shen Q, Zhang R. 2015. Whole transcriptomic analysis of the plant-beneficial rhizobacterium Bacillus amyloliquefaciens SQR9 during enhanced biofilm formation regulated by maize root exudates. BMC Genomics 16:685. doi:10.1186/s12864-015-1825-5. 26346121 PMC4562157

[57] Li C, Cheng P, Zheng L, Li Y, Chen Y, Wen S, Yu G. 2021. Comparative genomics analysis of two banana Fusarium wilt biocontrol endophytes Bacillus subtilis R31 and TR21 provides insights into their differences on phytobeneficial trait. Genomics 113:900–909. doi:10.1016/j.ygeno.2021.02.006. 33592313

[58] Sharma K, Ghiffary MR, Kim HU, Lee SY. 2020. Engineering heterologous hosts for the enhanced production of non-ribosomal peptides. Biotechnol Bioproc E 25:795–809. doi:10.1007/s12257-020-0080-z.

[59] Walsh CT. 2008. The chemical versatility of natural-product assembly lines. Acc Chem Res 41:4–10. doi:10.1021/ar7000414. 17506516

[60] Chikindas ML, Weeks R, Drider D, Chistyakov VA, Dicks LM. 2018. Functions and emerging applications of bacteriocins. Curr Opin Biotechnol 49:23–28. doi:10.1016/j.copbio.2017.07.011. 28787641 PMC5799035

[61] Sayers EW, Beck J, Bolton EE, Bourexis D, Brister JR, Canese K, Comeau DC, Funk K, Kim S, Klimke W, Marchler-Bauer A, Landrum M, Lathrop S, Lu Z, Madden TL, O’Leary N, Phan L, Rangwala SH, Schneider VA, Skripchenko Y, Wang J, Ye J, Trawick BW, Pruitt KD, Sherry ST. 2021. Database resources of the National Center for Biotechnology Information. Nucleic Acids Res 49:D10–D17. doi:10.1093/nar/gkaa892. 33095870 PMC7778943

[62] Avershina E, Frisli T, Rudi K. 2013. De novo semi-alignment of 16S rRNA gene sequences for deep phylogenetic characterization of next generation sequencing data. Microbes Environ 28:211–216. doi:10.1264/jsme2.me12157. 23603801 PMC4070667

[63] Schmidt MH-W, Vogel A, Denton AK, Istace B, Wormit A, van de Geest H, Bolger ME, Alseekh S, Maß J, Pfaff C, Schurr U, Chetelat R, Maumus F, Aury J-M, Koren S, Fernie AR, Zamir D, Bolger AM, Usadel B. 2017. De novo assembly of a new Solanum pennellii accession using nanopore sequencing. Plant Cell 29:2336–2348. doi:10.1105/tpc.17.00521. 29025960 PMC5774570

[64] Hadfield J, Eldridge MD. 2014. Multi-genome alignment for quality control and contamination screening of next-generation sequencing data. Front Genet 5:31. doi:10.3389/fgene.2014.00031. 24600470 PMC3930033

[65] Garrido SD, Sansegundo LP, Redondo NM, Suman J, Cajthaml T, Blanco RE, Martin M, Uhlik O, Rivilla R. 2020. Analysis of the biodegradative and adaptive potential of the novel polychlorinated biphenyl degrader Rhodococcus sp. WAY2 revealed by its complete genome sequence. Microb Genom 6:e000363. doi:10.1099/mgen.0.000363. 32238227 PMC7276702

[66] Daramola DS, Danso SKA, Hardarson G. 1994. Nodulation, N_2_ fixation and dry-matter yield of soybean [*Glycine max* (L.) merrill] inoculated with effective *Bradyrhizobium japonicum* strains. Soil Biol Biochem 26:883–889. doi:10.1016/0038-0717(94)90304-2.

[67] Lopes R, Cerdeira L, Tavares GS, Ruiz JC, Blom J, Horácio ECA, Mantovani HC, Queiroz MV. 2017. Genome analysis reveals insights of the endophytic Bacillus toyonensis BAC3151 as a potentially novel agent for biocontrol of plant pathogens. World J Microbiol Biotechnol 33:185. doi:10.1007/s11274-017-2347-x. 28948478

[68] Emms DM, Kelly S. 2019. OrthoFinder: phylogenetic orthology inference for comparative genomics. Genome Biol 20:238. doi:10.1186/s13059-019-1832-y. 31727128 PMC6857279

[69] Price MN, Dehal PS, Arkin AP. 2010. FastTree 2—approximately maximum-likelihood trees for large alignments. PLoS One 5:e9490. doi:10.1371/journal.pone.0009490. 20224823 PMC2835736

[70] Letunic I, Bork P. 2021. Interactive Tree Of Life (iTOL) v5: an online tool for phylogenetic tree display and annotation. Nucleic Acids Res 49:W293–W296. doi:10.1093/nar/gkab301. 33885785 PMC8265157

[71] Kim EB, Jin GD, Lee JY, Choi YJ. 2016. Genomic features and niche-adaptation of Enterococcus faecium strains from Korean soybean-fermented foods. PLoS One 11:e0153279. doi:10.1371/journal.pone.0153279. 27070419 PMC4829236

[72] Zheng Y, Gong X. 2019. Niche differentiation rather than biogeography shapes the diversity and composition of microbiome of Cycas panzhihuaensis. Microbiome 7:152. doi:10.1186/s40168-019-0770-y. 31791400 PMC6888988

[73] Hernández-González IL, Moreno-Hagelsieb G, Olmedo-Álvarez G. 2018. Environmentally-driven gene content convergence and the Bacillus phylogeny. BMC Evol Biol 18:148. doi:10.1186/s12862-018-1261-7. 30285626 PMC6171248

[74] Gautam A, Felderhoff H, Bağci C, Huson DH. 2022. Using AnnoTree to get more assignments, faster, in DIAMOND+MEGAN microbiome analysis. mSystems 7:e01408-21. doi:10.1128/msystems.01408-21. 35191776 PMC8862659

