# Supplementary material for: Comparative Functional Genome Analysis Reveals the Habitat Adaptation and Biocontrol Characteristics of Plant Growth-Promoting Bacteria in NCBI Databases
Source: Microbiol Spectr. 2023 Apr 26;11(3):e05007-22. doi: 10.1128/spectrum.05007-22 (PMC10269705; doi:10.1128/spectrum.05007-22)
Supplement: Supplemental file 1 — Supplemental material. Download spectrum.05007-22-s0001.pdf, PDF file, 2.0 MB [file spectrum.05007-22-s0001.pdf]

Supplemental material

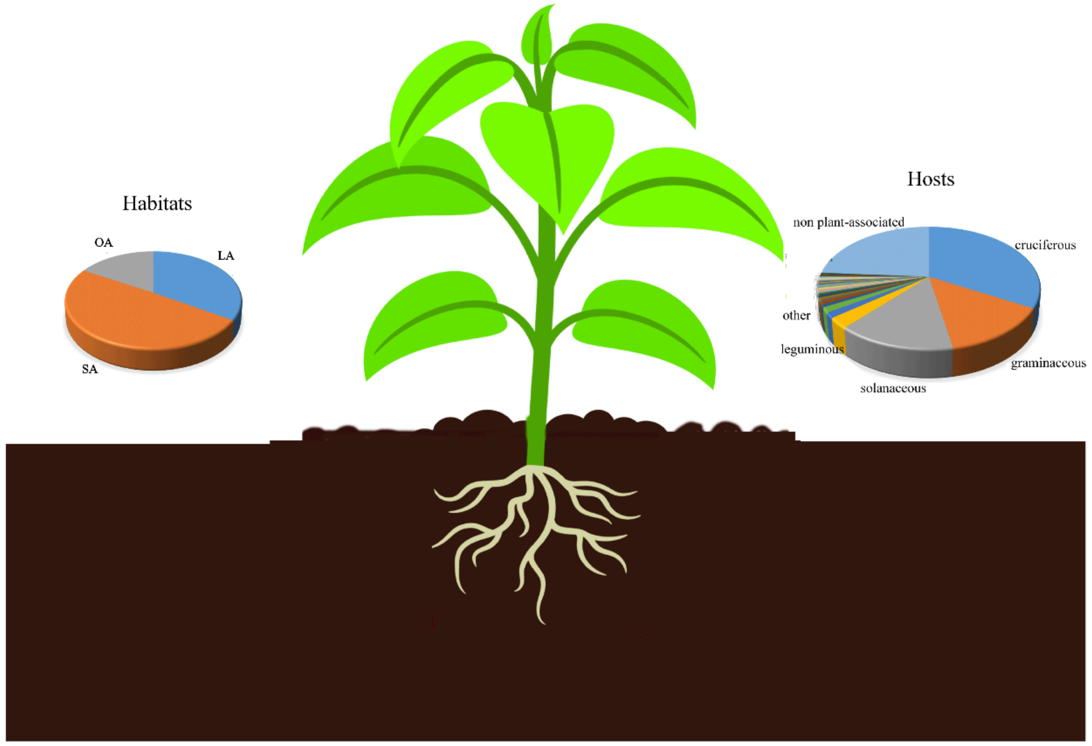

Fig. S1

In terms of the distribution of strains across habitats and host plants, the data can be visualized in a pie chart. Among the different habitats, leaf-associated (LA) strains accounted for 34% of the total, while other-associated (OA) strains accounted for 17%, and soil-associated (SA) strains made up the remaining 49%. In total, 36 plant species were found to be hosts for the strains, with cruciferous, graminaceous, solanaceous, and leguminous plants being the most common.

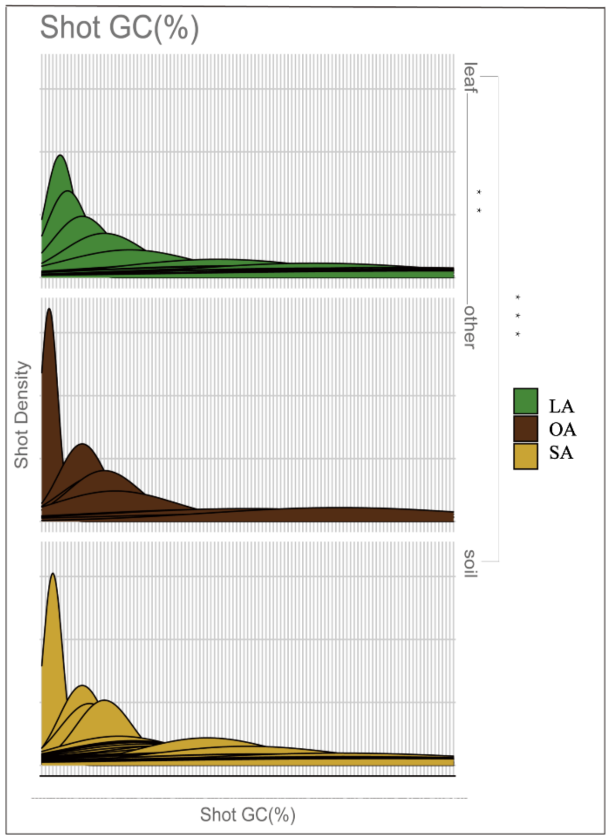

Fig. S2

Density plot of GC content of the genome of leaf-associated (LA), soil-associated (SA) PGPB and other-associated (OA) strains (*t*-test, p<0.05).

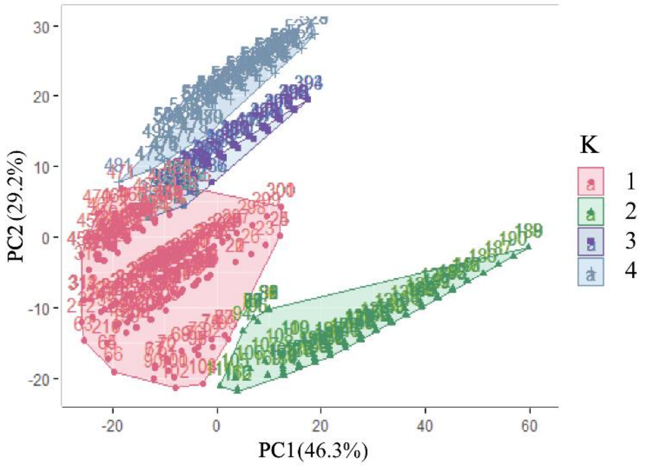

Fig. S3

K-medoids clustering display.

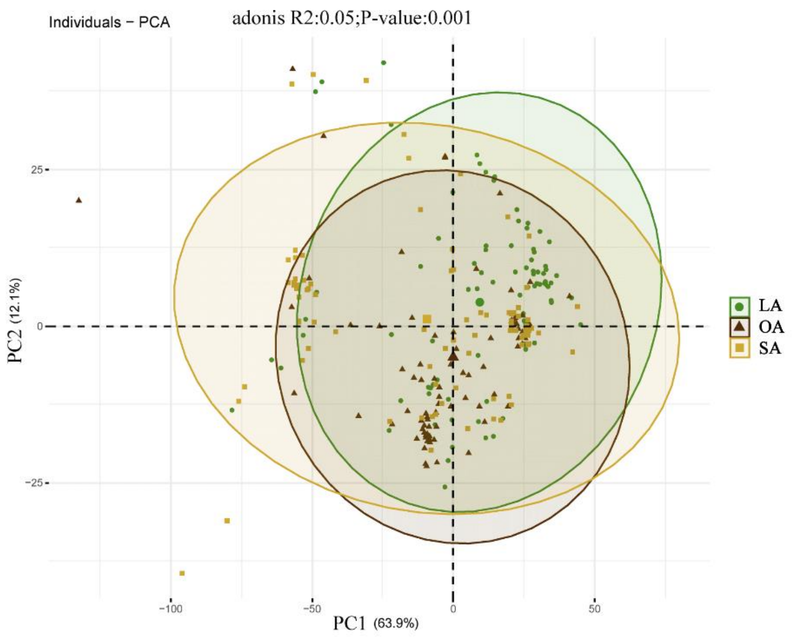

Fig. S4

Phylogenetically informed principal-component analysis (phylo-PCA) was conducted on the genes with significant differences between LA, SA PGPB and OA strains (Adonis test, *p*=0.001).

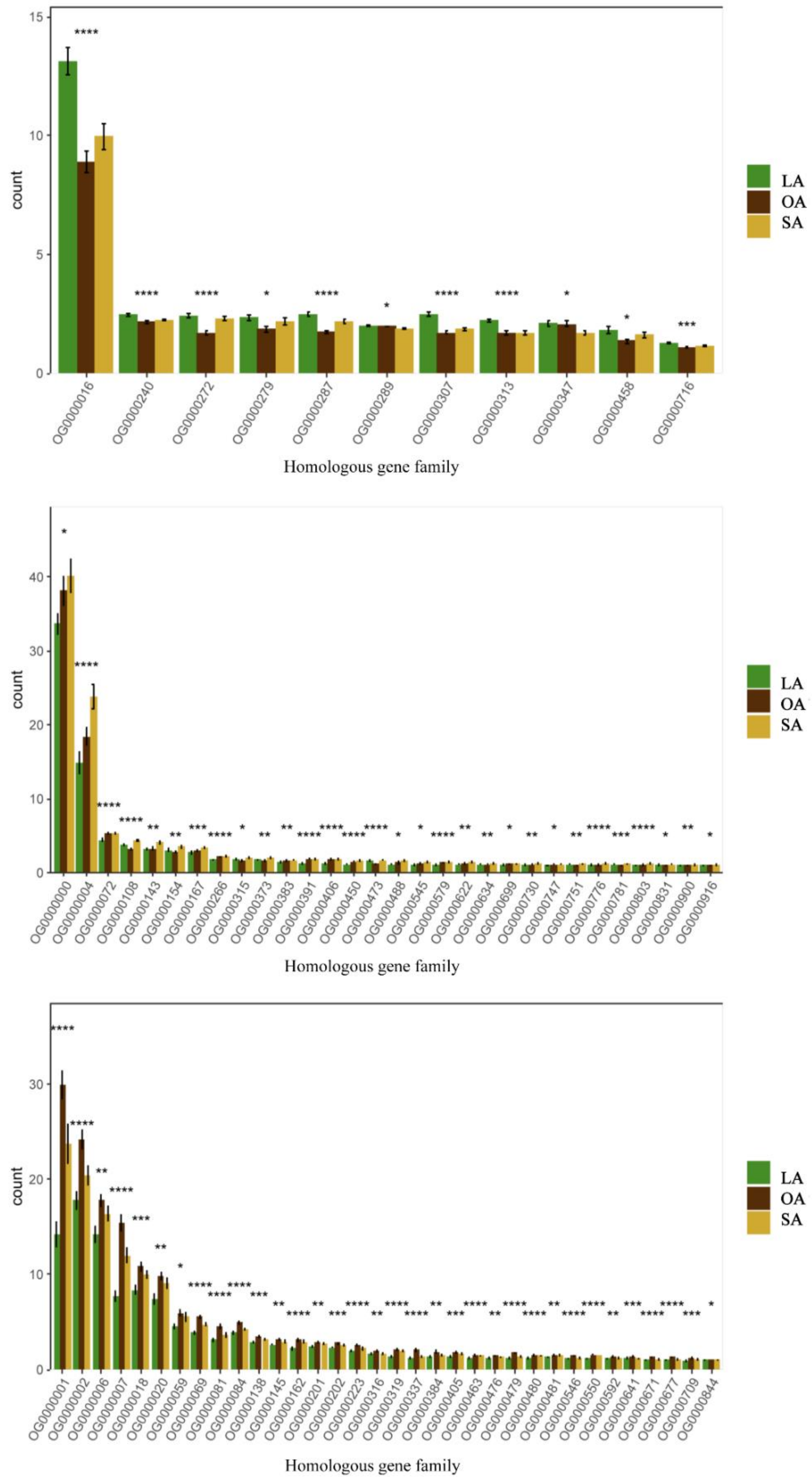

Fig. S5

Pairwise functional comparisons among leaf-associated (LA) and soil-associated (SA) PGPB strain genomes using NR database. (*t*- test, * 0.01<*p*<0.05, ** 0.001<*p*<0.01, *** 0.0001<*p*<0.001, **** *p*<0.0001). Bar charts represent the number of gene categories in statistical tests, and the error bars represent the standard deviations of the number of genes in the core differential genomes of LA/SA habitat strains.

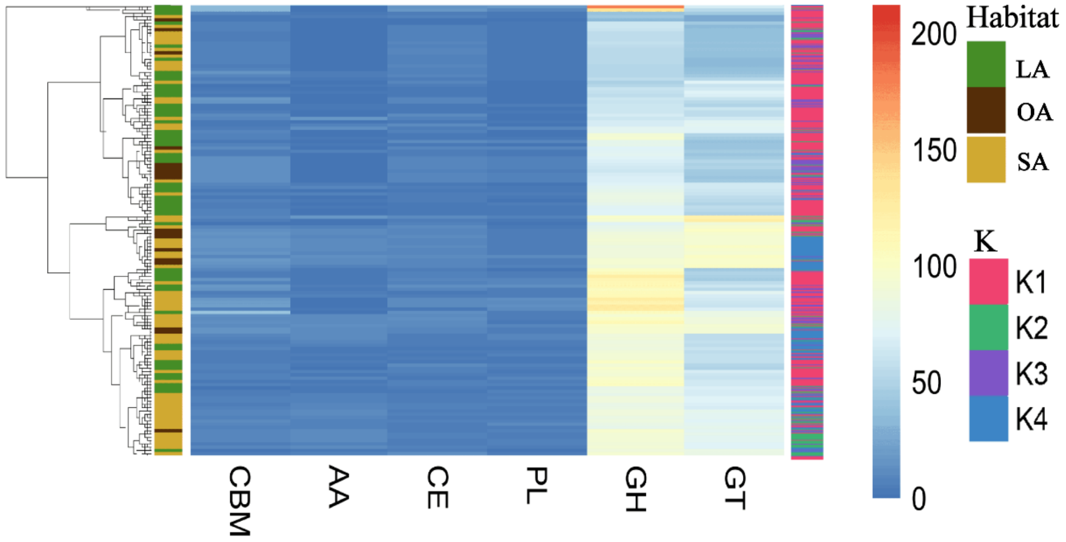

Fig. S6
Clustered heat map of PGPB carbohydrate enzymes.

Table. S1
Downloaded genome statistics and project information for 195 strains of LA PGPB, 95 strains of OA group, and 283 strains of SA PGPB from literature and the NCBI database.

LA PGPB

Number	Strain	Functions		Distribution			Genome sequence			Reference
		Promote plant growth	Inhibit pathogen infection	Isolation	Latitude	Longitude	Accession number	Size (bp)	GC (%)	
1	*Bacillus subtilis* LBUM979		√	Marijuana stem	55.6854	-106.457	GCF_009866865.1	4215646	43.5	(1)
2	*Bacillus subtilis* 7PJ-16	√	√	Mulberry leaf	29.5	106.25	GCF_016065415.1	4209045	43.5	(2)
3	*Pseudomonas chlororaphis* subsp. *piscium* ZJU60		√	Wheat head	36.1283	108.1333	GCF_003008635.1	6818002	63.1	(3)
4	*Bacillus velezensis* FZ06		√	Camellia leaves	36.1283	108.1333	GCF_011043745.1	3894518	46.4	(4)
5	*Burkholderia gladioli* CGB10		√	Sugar cane	23.1579	113.2732	GCF_014263355.1	8423203	68	(5)
6	*Bacillus subtilis* R31		√	Caulis dendrobii	23.1579	113.2732	GCF_015602625.1	4186822	43.5	(6)
7	*Bacillus velezensis* CC09		√	Camphor	31.3275	118.8921	GCF_001593395.2	4167153	46.4	https://www.ncbi.nlm.nih.gov/genome/annotation_prok/
8	*Pseudomonas fluorescens* A506	√		Pear	37.2639	-119.004	GCF_000262325.2	6019547	60	(7)
9	*Bacillus amyloliquefaciens* ZJU1		√	Mulberry	30.2084	120.212	GCF_007362635.1	4064151	46.4	(8)
10	*Bacillus velezensis* JS25R		√	Wheat	33.2011	120.501	GCF_000769555.1	4014440	46.4	(9)
11	*Serratia plymuthica* UBCF_13	√		Wheat	-4.8816	121.1218	GCF_018336935.1	5455815	56	https://www.ncbi.nlm.nih.gov/genome/annotation_prok/
12	*Bacillus velezensis* GYL4		√	Pepper	40.1914	127.5576	GCF_003071465.1	3975084	46.4	(10)

13	*Methylobacterium oryzae* CBMB20	√	√	Rice	35.9203	128.0083	GCF_000757795.1	6286629	69.8	(11)
14	*Bacillus subtilis* YB-04	√		Wheat	113.906	35.3718	GCF_017753785.1	4156177	43.5	(12)
15	*Pseudomonas putida* BR-PH17		√	Cycad	28.7757	70.24079	GCF_016406145.1	5854781	62.3	(13)
16	*Frigoribacterium* sp. Leaf8		√	Arabidopsis thaliana	47.4817	8.217547	GCF_001421165.1	3328572	71.3	(14)
17	*Rhizobium* sp. Leaf202		√	Arabidopsis thaliana	47.4817	8.217547	GCF_001421235.1	4785251	58	(14)
18	*Sphingomonas* sp. Leaf10		√	Arabidopsis thaliana	47.4817	8.217547	GCF_001421245.1	4135713	66.1	(14)
19	*Microbacterium* sp. Leaf203		√	Arabidopsis thaliana	47.4817	8.217547	GCF_001421275.1	3448852	69.6	(14)
20	*Sphingomonas* sp. Leaf11		√	Arabidopsis thaliana	47.4817	8.217547	GCF_001421285.1	3765831	66.1	(14)
21	*Plantibacter* sp. Leaf1		√	Arabidopsis thaliana	47.4817	8.217547	GCF_001421315.1	3894571	69.4	(14)
22	*Novosphingobium* sp. Leaf2		√	Arabidopsis thaliana	47.4817	8.217547	GCF_001421325.1	3715735	64.25	(14)
23	*Sphingomonas* sp. Leaf16		√	Arabidopsis thaliana	47.4817	8.217547	GCF_001421405.1	3945932	66.1	(14)
24	*Sphingomonas* sp. Leaf208		√	Arabidopsis thaliana	47.4817	8.217547	GCF_001421415.1	4703275	66.1	(14)
25	*Chryseobacterium* sp. Leaf201		√	Arabidopsis thaliana	47.4817	8.217547	GCF_001421435.1	4338957	36.5	(14)
26	*Agreia* sp. Leaf210		√	Arabidopsis thaliana	47.4817	8.217547	GCF_001421485.1	3542150	66.4107	(14)
27	*Sphingomonas* sp. Leaf17		√	Arabidopsis thaliana	47.4817	8.217547	GCF_001421505.1	3204003	66.1	(14)
28	*Pedobacter* sp. Leaf216		√	Arabidopsis thaliana	47.4817	8.217547	GCF_001421515.1	5680060	39	(14)
29	*Sphingomonas* sp. Leaf20		√	Arabidopsis thaliana	47.4817	8.217547	GCF_001421535.1	4313322	66.1	(14)
30	*Agromyces* sp. Leaf222		√	Arabidopsis thaliana	47.4817	8.217547	GCF_001421565.1	4428049	70.9	(14)
31	*Sphingomonas* sp. Leaf23		√	Arabidopsis thaliana	47.4817	8.217547	GCF_001421585.1	3804425	66.1	(14)
32	*Sphingomonas* sp. Leaf24		√	Arabidopsis thaliana	47.4817	8.217547	GCF_001421605.1	4001919	66.1	(14)

33	*Sphingomonas* sp. Leaf226		√	Arabidopsis thaliana	47.4817	8.217547	GCF_001421625.1	4271556	66.1	(14)
34	*Sphingomonas* sp. Leaf25		√	Arabidopsis thaliana	47.4817	8.217547	GCF_001421635.1	4000316	66.1	(14)
35	*Sphingomonas* sp. Leaf26		√	Arabidopsis thaliana	47.4817	8.217547	GCF_001421665.1	4822147	63.4	(14)
36	*Sphingomonas* sp. Leaf28		√	Arabidopsis thaliana	47.4817	8.217547	GCF_001421685.1	4454691	66.1	(14)
37	*Xylophilus* sp. Leaf220		√	Arabidopsis thaliana	47.4817	8.217547	GCF_001421705.1	4483623	68.6	(14)
38	*Sphingomonas* sp. Leaf22		√	Arabidopsis thaliana	47.4817	8.217547	GCF_001421715.1	4216368	66.1	(14)
39	*Sphingomonas* sp. Leaf230		√	Arabidopsis thaliana	47.4817	8.217547	GCF_001421745.1	4143790	66.1	(14)
40	*Sphingomonas* sp. Leaf32		√	Arabidopsis thaliana	47.4817	8.217547	GCF_001421765.1	3946283	66.1	(14)
41	*Sphingomonas* sp. Leaf34		√	Arabidopsis thaliana	47.4817	8.217547	GCF_001421805.1	3943835	66.1	(14)
42	*Sphingomonas* sp. Leaf38		√	Arabidopsis thaliana	47.4817	8.217547	GCF_001421825.1	3994960	66.1	(14)
43	*Frigoribacterium* sp. Leaf44		√	Arabidopsis thaliana	47.4817	8.217547	GCF_001421865.1	3373506	71.3	(14)
44	*Pseudomonas* sp. Leaf48		√	Arabidopsis thaliana	47.4817	8.217547	GCF_001421885.1	5689447	60.8	(14)
45	*Duganella* sp. Leaf61		√	Arabidopsis thaliana	47.4817	8.217547	GCF_001421905.1	6231257	62.9	(14)
46	*Sphingomonas* sp. Leaf62		√	Arabidopsis thaliana	47.4817	8.217547	GCF_001421915.1	4142413	66.1	(14)
47	*Devosia* sp. Leaf64		√	Arabidopsis thaliana	47.4817	8.217547	GCF_001421945.1	4252528	62.6	(14)
48	*Sphingomonas* sp. Leaf67		√	Arabidopsis thaliana	47.4817	8.217547	GCF_001421965.1	4192908	66.1	(14)
49	*Sphingomonas* sp. Leaf231		√	Arabidopsis thaliana	47.4817	8.217547	GCF_001421995.1	3733197	66.1	(14)
50	*Stenotrophomonas* sp. Leaf70		√	Arabidopsis thaliana	47.4817	8.217547	GCF_001422025.1	4032657	66.7	(14)
51	*Sphingomonas* sp. Leaf242		√	Arabidopsis thaliana	47.4817	8.217547	GCF_001422045.1	4300070	66.1	(14)
52	*Acidovorax* sp. Leaf76		√	Arabidopsis thaliana	47.4817	8.217547	GCF_001422065.1	5870153	65.1	(14)
53	*Pseudomonas* sp. Leaf83		√	Arabidopsis thaliana	47.4817	8.217547	GCF_001422075.1	5538830	62	(14)

54	*Acidovorax* sp. Leaf84		√	Arabidopsis thaliana	47.4817	8.217547	GCF_001422105.1	5869697	65.1	(14)
55	*Frigoribacterium* sp. Leaf254		√	Arabidopsis thaliana	47.4817	8.217547	GCF_001422145.1	3423974	71.3	(14)
56	*Sphingomonas* sp. Leaf257		√	Arabidopsis thaliana	47.4817	8.217547	GCF_001422185.1	4414563	66.1	(14)
57	*Curtobacterium* sp. Leaf261		√	Arabidopsis thaliana	47.4817	8.217547	GCF_001422205.1	3926430	71	(14)
58	*Methylobacterium* sp. Leaf89		√	Arabidopsis thaliana	47.4817	8.217547	GCF_001422215.1	5005286	69	(14)
59	*Rhizobium* sp. Leaf262		√	Arabidopsis thaliana	47.4817	8.217547	GCF_001422245.1	5046934	60.8873	(14)
60	*Frigoribacterium* sp. Leaf263		√	Arabidopsis thaliana	47.4817	8.217547	GCF_001422285.1	3243570	71.3	(14)
61	*Leifsonia* sp. Leaf264		√	Arabidopsis thaliana	47.4817	8.217547	GCF_001422325.1	4540635	69.65	(14)
62	*Pseudorhodoferax* sp. Leaf265		√	Arabidopsis thaliana	47.4817	8.217547	GCF_001422365.1	7476079	69.05	(14)
63	*Methylobacterium* sp. Leaf99		√	Arabidopsis thaliana	47.4817	8.217547	GCF_001422375.1	4645412	69	(14)
64	*Pseudorhodoferax* sp. Leaf267		√	Arabidopsis thaliana	47.4817	8.217547	GCF_001422405.1	6530331	69.05	(14)
65	*Pseudorhodoferax* sp. Leaf274		√	Arabidopsis thaliana	47.4817	8.217547	GCF_001422445.1	6735869	69.05	(14)
66	*Agreia* sp. Leaf283		√	Arabidopsis thaliana	47.4817	8.217547	GCF_001422485.1	3978453	66.4107	(14)
67	*Sphingomonas* sp. Leaf29		√	Arabidopsis thaliana	47.4817	8.217547	GCF_001422495.1	3945508	66.1	(14)
68	*Sphingomonas* sp. Leaf30		√	Arabidopsis thaliana	47.4817	8.217547	GCF_001422525.1	4403081	66.1	(14)
69	*Serratia* sp. Leaf51		√	Arabidopsis thaliana	47.4817	8.217547	GCF_001422575.1	5420896	53	(14)
70	*Erwinia* sp. Leaf53		√	Arabidopsis thaliana	47.4817	8.217547	GCF_001422605.1	5115519	54.35	(14)
71	*Arthrobacter* sp. Leaf69		√	Arabidopsis thaliana	47.4817	8.217547	GCF_001422645.1	4140548	65.6	(14)

72	*Arthrobacter* sp. Leaf234		√	Arabidopsis thaliana	47.4817	8.217547	GCF_001422665.1	3524245	65.6	(14)
73	*Paenibacillus* sp. Leaf72		√	Arabidopsis thaliana	47.4817	8.217547	GCF_001422685.1	7576528	47.2	(14)
74	*Agreia* sp. Leaf244		√	Arabidopsis thaliana	47.4817	8.217547	GCF_001422695.1	4022387	66.4107	(14)
75	*Flavobacterium* sp. Leaf82		√	Arabidopsis thaliana	47.4817	8.217547	GCF_001422725.1	5674764	34.3	(14)
76	*Acidovorax* sp. Leaf78		√	Arabidopsis thaliana	47.4817	8.217547	GCF_001422735.1	5749411	65.1	(14)
77	*Methylobacterium* sp. Leaf88		√	Arabidopsis thaliana	47.4817	8.217547	GCF_001422795.1	5031274	69	(14)
78	*Aeromicrobium* sp. Leaf272		√	Arabidopsis thaliana	47.4817	8.217547	GCF_001422865.1	3477274	70.4	(14)
79	*Methylobacterium* sp. Leaf104		√	Arabidopsis thaliana	47.4817	8.217547	GCF_001422885.1	4968001	69	(14)
80	*Methylobacterium* sp. Leaf106		√	Arabidopsis thaliana	47.4817	8.217547	GCF_001422895.1	4759318	69	(14)
81	*Microbacterium* sp. Leaf288		√	Arabidopsis thaliana	47.4817	8.217547	GCF_001422925.1	4747796	69.6	(14)
82	*Methylobacterium* sp. Leaf117		√	Arabidopsis thaliana	47.4817	8.217547	GCF_001422985.1	5320575	69	(14)
83	*Rathayibacter* sp. Leaf294		√	Arabidopsis thaliana	47.4817	8.217547	GCF_001423005.1	4108248	72.3	(14)
84	*Rathayibacter* sp. Leaf296		√	Arabidopsis thaliana	47.4817	8.217547	GCF_001423045.1	4022070	72.3	(14)
85	*Methylobacterium* sp. Leaf125		√	Arabidopsis thaliana	47.4817	8.217547	GCF_001423085.1	5452632	69	(14)
86	*Frondihabitans* sp. Leaf304		√	Arabidopsis thaliana	47.4817	8.217547	GCF_001423105.1	3453774	67.45	(14)
87	*Duganella* sp. Leaf126		√	Arabidopsis thaliana	47.4817	8.217547	GCF_001423125.1	5315014	62.9	(14)
88	*Pseudomonas* sp. Leaf127		√	Arabidopsis thaliana	47.4817	8.217547	GCF_001423155.1	6376752	60.8	(14)
89	*Acinetobacter* sp. Leaf130		√	Arabidopsis thaliana	47.4817	8.217547	GCF_001423205.1	4045863	40.8	(14)

90	*Rhizobium* sp. Leaf321		√	Arabidopsis thaliana	47.4817	8.217547	GCF_001423215.1	4922079	60.8873	(14)
91	*Aureimonas* sp. Leaf324		√	Arabidopsis thaliana	47.4817	8.217547	GCF_001423245.1	4415253	66.9	(14)
92	*Methylobacterium* sp. Leaf108		√	Arabidopsis thaliana	47.4817	8.217547	GCF_001423265.1	4465776	69	(14)
93	*Methylobacterium* sp. Leaf111		√	Arabidopsis thaliana	47.4817	8.217547	GCF_001423285.1	5166228	69	(14)
94	*Methylobacterium* sp. Leaf112		√	Arabidopsis thaliana	47.4817	8.217547	GCF_001423295.1	4693153	69	(14)
95	*Methylobacterium* sp. Leaf113		√	Arabidopsis thaliana	47.4817	8.217547	GCF_001423325.1	5151901	69	(14)
96	*Methylobacterium* sp. Leaf123		√	Arabidopsis thaliana	47.4817	8.217547	GCF_001423405.1	5437666	69	(14)
97	*Rhizobium* sp. Leaf306		√	Arabidopsis thaliana	47.4817	8.217547	GCF_001423425.1	5009159	60.8873	(14)
98	*Rhizobium* sp. Leaf311		√	Arabidopsis thaliana	47.4817	8.217547	GCF_001423445.1	4847754	60.8873	(14)
99	*Pseudomonas* sp. Leaf129		√	Arabidopsis thaliana	47.4817	8.217547	GCF_001423465.1	4877329	60.8	(14)
100	*Microbacterium* sp. Leaf320		√	Arabidopsis thaliana	47.4817	8.217547	GCF_001423485.1	3866076	69.6	(14)
101	*Xanthomonas* sp. Leaf131		√	Arabidopsis thaliana	47.4817	8.217547	GCF_001423495.1	5169088	68.9	(14)
102	*Arthrobacter* sp. Leaf137		√	Arabidopsis thaliana	47.4817	8.217547	GCF_001423525.1	4375549	66	(14)
103	*Leifsonia* sp. Leaf325		√	Arabidopsis thaliana	47.4817	8.217547	GCF_001423545.1	3732125	69.65	(14)
104	*Arthrobacter* sp. Leaf145		√	Arabidopsis thaliana	47.4817	8.217547	GCF_001423565.1	4493998	63	(14)
105	*Xanthomonas* sp. Leaf148		√	Arabidopsis thaliana	47.4817	8.217547	GCF_001423585.1	5217728	68.9	(14)
106	*Agreia* sp. Leaf335		√	Arabidopsis thaliana	47.4817	8.217547	GCF_001423605.1	3550532	66.4107	(14)
107	*Microbacterium* sp. Leaf159		√	Arabidopsis thaliana	47.4817	8.217547	GCF_001423615.1	3887207	69.6	(14)

108	*Microbacterium* sp. Leaf161		√	Arabidopsis thaliana	47.4817	8.217547	GCF_001423645.1	3630887	69.6	(14)
109	*Frigoribacterium* sp. Leaf164		√	Arabidopsis thaliana	47.4817	8.217547	GCF_001423665.1	3287584	71.3	(14)
110	*Leifsonia* sp. Leaf336		√	Arabidopsis thaliana	47.4817	8.217547	GCF_001423695.1	4157793	69.65	(14)
111	*Plantibacter* sp. Leaf171		√	Arabidopsis thaliana	47.4817	8.217547	GCF_001423725.1	3893704	69.4	(14)
112	*Arthrobacter* sp. Leaf337		√	Arabidopsis thaliana	47.4817	8.217547	GCF_001423745.1	5201172	65.6	(14)
113	*Sphingomonas* sp. Leaf343		√	Arabidopsis thaliana	47.4817	8.217547	GCF_001423765.1	3600029	66.1	(14)
114	*Microbacterium* sp. Leaf179		√	Arabidopsis thaliana	47.4817	8.217547	GCF_001423785.1	3533805	69.6	(14)
115	*Microbacterium* sp. Leaf347		√	Arabidopsis thaliana	47.4817	8.217547	GCF_001423805.1	2942136	69.6	(14)
116	*Microbacterium* sp. Leaf351		√	Arabidopsis thaliana	47.4817	8.217547	GCF_001423825.1	2888800	69.6	(14)
117	*Sphingomonas* sp. Leaf357		√	Arabidopsis thaliana	47.4817	8.217547	GCF_001423845.1	3871084	66.1	(14)
118	*Rathayibacter* sp. Leaf185		√	Arabidopsis thaliana	47.4817	8.217547	GCF_001423885.1	4105523	72.3	(14)
119	*Frigoribacterium* sp. Leaf186		√	Arabidopsis thaliana	47.4817	8.217547	GCF_001423905.1	3439013	71.3	(14)
120	*Exiguobacterium* sp. Leaf187		√	Arabidopsis thaliana	47.4817	8.217547	GCF_001423925.1	3309560	47.7	(14)
121	*Pedobacter* sp. Leaf194		√	Arabidopsis thaliana	47.4817	8.217547	GCF_001423945.1	4573096	39	(14)
122	*Sphingomonas* sp. Leaf198		√	Arabidopsis thaliana	47.4817	8.217547	GCF_001424005.1	4201813	66.1	(14)
123	*Brevundimonas* sp. Leaf363		√	Arabidopsis thaliana	47.4817	8.217547	GCF_001424025.1	3127470	67.2	(14)
124	*Rhizobium* sp. Leaf371		√	Arabidopsis thaliana	47.4817	8.217547	GCF_001424045.1	5085900	60.8873	(14)
125	*Rhizobium* sp. Leaf384		√	Arabidopsis thaliana	47.4817	8.217547	GCF_001424065.1	4881596	60.8873	(14)

126	*Rhizobium* sp. Leaf386		√	Arabidopsis thaliana	47.4817	8.217547	GCF_001424085.1	6578697	60.8873	(14)
127	*Chryseobacterium* sp. Leaf404		√	Arabidopsis thaliana	47.4817	8.217547	GCF_001424145.1	3939915	36.5	(14)
128	*Massilia* sp. Leaf139		√	Arabidopsis thaliana	47.4817	8.217547	GCF_001424165.1	5387355	65.5	(14)
129	*Deinococcus* sp. Leaf326		√	Arabidopsis thaliana	47.4817	8.217547	GCF_001424185.1	4602077	67.6	(14)
130	*Cellulomonas* sp. Leaf334		√	Arabidopsis thaliana	47.4817	8.217547	GCF_001424195.1	4742537	73.5	(14)
131	*Microbacterium* sp. Leaf151		√	Arabidopsis thaliana	47.4817	8.217547	GCF_001424225.1	3609602	69.6	(14)
132	*Rhizobium* sp. Leaf155		√	Arabidopsis thaliana	47.4817	8.217547	GCF_001424245.1	4942144	58	(14)
133	*Acidovorax* sp. Leaf160		√	Arabidopsis thaliana	47.4817	8.217547	GCF_001424265.1	4717897	65.1	(14)
134	*Frigoribacterium* sp. Leaf172		√	Arabidopsis thaliana	47.4817	8.217547	GCF_001424285.1	3246786	71.3	(14)
135	*Pedobacter* sp. Leaf176		√	Arabidopsis thaliana	47.4817	8.217547	GCF_001424305.1	4753721	39	(14)
136	*Rhizobium* sp. Leaf341		√	Arabidopsis thaliana	47.4817	8.217547	GCF_001424325.1	4656797	60.8873	(14)
137	*Burkholderia* sp. Leaf177		√	Arabidopsis thaliana	47.4817	8.217547	GCF_001424345.1	5874930	66.6211	(14)
138	*Williamsia* sp. Leaf354		√	Arabidopsis thaliana	47.4817	8.217547	GCF_001424365.1	4566645	64.8	(14)
139	*Curtobacterium* sp. Leaf183		√	Arabidopsis thaliana	47.4817	8.217547	GCF_001424385.1	3580776	71	(14)
140	*Dyadobacter* sp. Leaf189		√	Arabidopsis thaliana	47.4817	8.217547	GCF_001424405.1	6065072	45.7	(14)
141	*Acidovorax* sp. Leaf191		√	Arabidopsis thaliana	47.4817	8.217547	GCF_001424425.1	5870193	65.1	(14)
142	*Methylobacterium* sp. Leaf361		√	Arabidopsis thaliana	47.4817	8.217547	GCF_001424445.1	6849062	69	(14)
143	*Geodermatophilus* sp. Leaf369		√	Arabidopsis thaliana	47.4817	8.217547	GCF_001424455.1	4368819	74.6	(14)
144	*Modestobacter* sp. Leaf380		√	Arabidopsis thaliana	47.4817	8.217547	GCF_001424485.1	4764686	73.4	(14)

145	*Rhizobium* sp. Leaf391		√	Arabidopsis thaliana	47.4817	8.217547	GCF_001424505.1	6560665	60.8873	(14)
146	*Methylobacterium* sp. Leaf399		√	Arabidopsis thaliana	47.4817	8.217547	GCF_001424525.1	4456718	69	(14)
147	*Ramlibacter* sp. Leaf400		√	Arabidopsis thaliana	47.4817	8.217547	GCF_001424545.1	4585682	68.2	(14)
148	*Arthrobacter* sp. Leaf141		√	Arabidopsis thaliana	47.4817	8.217547	GCF_001424565.1	4355205	65.6	(14)
149	*Chryseobacterium* sp. Leaf180		√	Arabidopsis thaliana	47.4817	8.217547	GCF_001424585.1	3154203	36.5	(14)
150	*Curtobacterium* sp. Leaf154		√	Arabidopsis thaliana	47.4817	8.217547	GCF_001424595.1	3640711	71	(14)
151	*Bradyrhizobium* sp. Leaf396		√	Arabidopsis thaliana	47.4817	8.217547	GCF_001424605.1	7709278	63.5	(14)
152	*Frigoribacterium* sp. Leaf415		√	Arabidopsis thaliana	47.4817	8.217547	GCF_001424645.1	3423183	71.3	(14)
153	*Methylophilus* sp. Leaf416		√	Arabidopsis thaliana	47.4817	8.217547	GCF_001424665.1	2940342	50.4	(14)
154	*Aureimonas* sp. Leaf454		√	Arabidopsis thaliana	47.4817	8.217547	GCF_001424685.1	4797175	66.9	(14)
155	*Microbacterium* sp. Leaf436		√	Arabidopsis thaliana	47.4817	8.217547	GCF_001424715.1	3367539	69.6	(14)
156	*Methylobacterium* sp. Leaf469		√	Arabidopsis thaliana	47.4817	8.217547	GCF_001424745.1	4700855	69	(14)
157	*Marmoricola* sp. Leaf446		√	Arabidopsis thaliana	47.4817	8.217547	GCF_001424755.1	3814797	72.2	(14)
158	*Rhizobium* sp. Leaf383		√	Arabidopsis thaliana	47.4817	8.217547	GCF_001425345.1	5014728	60.8873	(14)
159	*Chryseobacterium* sp. Leaf405		√	Arabidopsis thaliana	47.4817	8.217547	GCF_001425355.1	4492507	36.5	(14)
160	*Sphingomonas* sp. Leaf407		√	Arabidopsis thaliana	47.4817	8.217547	GCF_001425385.1	3784018	66.1	(14)
161	*Sphingomonas* sp. Leaf412		√	Arabidopsis thaliana	47.4817	8.217547	GCF_001425405.1	3462693	66.1	(14)

162	*Methylophilus* sp. Leaf414		√	Arabidopsis thaliana	47.4817	8.217547	GCF_001425425.1	3028624	50.4	(14)
163	*Devosia* sp. Leaf420		√	Arabidopsis thaliana	47.4817	8.217547	GCF_001425445.1	4219601	62.6	(14)
164	*Methylobacterium* sp. Leaf456		√	Arabidopsis thaliana	47.4817	8.217547	GCF_001425465.1	5577040	69	(14)
165	*Aureimonas* sp. Leaf427		√	Arabidopsis thaliana	47.4817	8.217547	GCF_001425485.1	5438231	66.9	(14)
166	*Methylophilus* sp. Leaf459		√	Arabidopsis thaliana	47.4817	8.217547	GCF_001425495.1	2948005	50.4	(14)
167	*Aureimonas* sp. Leaf460		√	Arabidopsis thaliana	47.4817	8.217547	GCF_001425525.1	5421504	66.9	(14)
168	*Pseudomonas* sp. Leaf434		√	Arabidopsis thaliana	47.4817	8.217547	GCF_001425545.1	5868027	60.8	(14)
169	*Methylobacterium* sp. Leaf466		√	Arabidopsis thaliana	47.4817	8.217547	GCF_001425565.1	4300541	69	(14)
170	*Aurantimonas* sp. Leaf443		√	Arabidopsis thaliana	47.4817	8.217547	GCF_001425575.1	3908067	67	(14)
171	*Rhizobium* sp. Leaf453		√	Arabidopsis thaliana	47.4817	8.217547	GCF_001425605.1	6368454	60.8873	(14)
172	*Sanguibacter* sp. Leaf3		√	Arabidopsis thaliana	47.4817	8.217547	GCF_001425965.1	4392999	68.3	(14)
173	*Rhodococcus* sp. Leaf7		√	Arabidopsis thaliana	47.4817	8.217547	GCF_001425985.1	4536885	65.2	(14)
174	*Bacillus* sp. Leaf13		√	Arabidopsis thaliana	47.4817	8.217547	GCF_001426005.1	5993972	37.5	(14)
175	*Bacillus* sp. Leaf75		√	Arabidopsis thaliana	47.4817	8.217547	GCF_001426025.1	6031561	37	(14)
176	*Methylobacterium* sp. Leaf94		√	Arabidopsis thaliana	47.4817	8.217547	GCF_001426045.1	5247959	69	(14)
177	*Rhodococcus* sp. Leaf258		√	Arabidopsis thaliana	47.4817	8.217547	GCF_001426065.1	4709617	65.2	(14)
178	*Rhodococcus* sp. Leaf278		√	Arabidopsis thaliana	47.4817	8.217547	GCF_001426085.1	5721797	65.2	(14)
179	*Bacillus* sp. Leaf406		√	Arabidopsis thaliana	47.4817	8.217547	GCF_001426105.1	4301683	48	(14)
180	*Bacillus* sp. Leaf49		√	Arabidopsis thaliana	47.4817	8.217547	GCF_001426125.1	3934505	40.5	(14)
181	*Rhodococcus* sp. Leaf225		√	Arabidopsis thaliana	47.4817	8.217547	GCF_001426145.1	4713325	65.2	(14)
182	*Rhodococcus* sp. Leaf233		√	Arabidopsis thaliana	47.4817	8.217547	GCF_001426165.1	5534279	65.2	(14)

183	*Rhodococcus* sp. Leaf247		√	Arabidopsis thaliana	47.4817	8.217547	GCF_001426185.1	4533613	65.2	(14)
184	*Bosea* sp. Leaf344		√	Arabidopsis thaliana	47.4817	8.217547	GCF_001426225.1	4350923	66.6	(14)
185	*Pseudomonas* sp. Leaf58		√	Arabidopsis thaliana	46.5222	8.517857	GCF_003627215.1	6335721	60.8	(14)
186	*Pseudomonas* sp. Leaf15		√	Arabidopsis thaliana	48.556	9.134944	GCF_001421425.1	6556788	60.8	(14)
187	*Pseudomonas* sp. Leaf98		√	Arabidopsis thaliana	48.556	9.134944	GCF_003258395.1	6543211	60.8	(14)
188	*Klebsiella pneumoniae* N4b		√	Neem	28	77	GCF_003408615.1	6074707	57.5	(15)
189	*Burkholderia ambifaria* BCC0197		√	Renal fern	39.5509	-101.563	GCF_902829415.1	7384442	66.9	(16)
190	*Rathayibacter* sp. Leaf299		√	Arabidopsis thaliana	47.4817	8.217547	GCF_001423055.1	4017524	71.5	(14)
191	*Pseudomonas putida* 1290	√		Pear	37.2639	-119.004	GCF_005080685.1	6610151	62.3	(17)
192	*Bacillus subtilis* SG6		√	Luffa	39.59	116.1	GCF_000782835.1	4079669	43.5	(18)
193	*Bacillus altitudinis* GLB197		√	Grape	33.79	114.5	GCF_001908475.1	4072961	41.4	(19)
194	*Burkholderia gladioli* BBB-01		√	Rice	24.1432	120.6799	GCF_016698705.1	8201484	68	(20)
195	*Methylobacterium phyllosphaerae* CBMB27		√	Trifolium repens and Cerastium holosteoides	38.8593	126.3112	GCF_001936175.1	6316624	69.8	(21)

OA group

Number	Strain	Functions		Distribution			Genome sequence			Reference
		Promote plant growth	Inhibit pathogen infection	Isolation	Latitude	Longitude	Accession number	Size (bp)	GC (%)	
1	*Paraburkholderia graminis* C4D1M	√		Soil near an artificial pond	46.5053	0.08593	GCF_000172415.1	7494154	63.3	(22)
2	*Lactobacillus acidophilus* DSM20079		√	Fermented dairy products	47.3372	0.757431	GCF_003047065.1	2009973	34.7	(23)
3	*Bacillus velezensis* QST713		√	Commercial product	47.3372	0.757431	GCF_003073255.1	4233757	46.4	(24)
4	*Klebsiella pneumoniae* Xen39		√	Wounds	39.5509	-101.563	GCF_010586985.1	5771740	57.5	(25)
5	*Burkholderia ambifaria* BCC1214		√	Environment	39.5509	-101.563	GCF_902829595.1	7605416	66.9	(16)
6	*Burkholderia ambifaria* BCC0478		√	Sputum	39.5509	-101.563	GCF_902829665.1	7236183	66.9	(16)
7	*Burkholderia ambifaria* BCC0480		√	Environment	39.5509	-101.563	GCF_902829735.1	7836177	66.9	(16)
8	*Burkholderia ambifaria* BCC0118		√	Sputum	39.5509	-101.563	GCF_902829755.1	7499163	66.9	(16)
9	*Burkholderia ambifaria* BCC0399		√	Human	39.5509	-101.563	GCF_902829775.1	7399973	66.9	(16)
10	*Burkholderia ambifaria* BCC0192		√	Corn rhizophere	39.5509	-101.563	GCF_902829785.1	7400790	66.9	(16)
11	*Burkholderia ambifaria* BCC1216		√	Environment	39.5509	-101.563	GCF_902829865.1	7458632	66.9	(16)
12	*Burkholderia ambifaria* BCC1213		√	Environment	39.5509	-101.563	GCF_902829925.1	7473930	66.9	(16)

13	*Burkholderia ambifaria* BCC1218		√	Environment	39.5509	-101.563	GCF_902830025.1	7456680	66.9	(16)
14	*Burkholderia ambifaria* BCC1240		√	Environment	39.5509	-101.563	GCF_902830085.1	7396890	66.9	(16)
15	*Burkholderia ambifaria* BCC1223		√	Environment	39.5509	-101.563	GCF_902830105.1	7605897	66.9	(16)
16	*Burkholderia ambifaria* BCC1265		√	Environment	39.5509	-101.563	GCF_902830115.1	7615093	66.9	(16)
17	*Burkholderia ambifaria* BCC1248		√	Environment	39.5509	-101.563	GCF_902830135.1	8030355	66.9	(16)
18	*Burkholderia ambifaria* BCC1249		√	Environment	39.5509	-101.563	GCF_902830255.1	7580283	66.9	(16)
19	*Bacillus velezensis* W1		√	The active fractions by HPLC purification	24.9314	102.4772	GCF_003265265.1	4237431	46.4	(26)
20	*Bacillus velezensis* L-H15		√	Cucumber seedling substrate	36.1283	108.1333	GCF_000833005.1	3905973	46.4	(27)
21	*Bacillus velezensis* SYBC H47		√	Raw honey	36.1283	108.1333	GCF_001854345.1	3884433	46.4	(28)
22	*Bacillus subtilis* SEM-9	√	√	Silkworm excrement	36.1283	108.1333	GCF_006165085.1	4121982	43.5	(29)
23	*Bacillus velezensis* WLYS23		√	Snakehead fish intestinal	23.04	113.13	GCF_013367755.1	3929662	46.4	(30)
24	*Bacillus subtilis* CW14		√	Elk droppings	39.59	116.1	GCF_002163815.1	4282516	43.5	(31)
25	*Bacillus licheniformis* BL-010		√	Aflatoxin contaminated corn feed	39.59	116.21	GCF_002236895.1	4287720	46.3	(32)
26	*Klebsiella* sp. A52		√	Marine sediment sample	25.0984	117.0374	GCF_018388785.1	5079954	57.1	(33)
27	*Bacillus subtilis* BYS2		√	The surface of Mount Tai of soil sample	36.2014	117.0875	GCF_018394175.1	4030791	43.5	(34)

28	*Bacillus amyloliquefaciens* ALB65		√	Hami melon peel	39.35	-119.004	GCF_003149715.1	4041665	46.4	(35)
29	*Bacillus subtilis* BL-01		√	Shrimp culture ponds	34.8407	119.1773	GCF_013393725.1	4242126	43.5	(36)
30	*Bacillus subtilis* BJQ0005	√		Baijiu	26.0747	119.2967	GCF_009884005.1	4112497	43.5	(37)
31	*Burkholderia ambifaria* BCC1052		√	Environment	42.4779	12.67888	GCF_902829435.1	7325939	66.9	(16)
32	*Burkholderia ambifaria* BCC1062		√	Environment	42.4779	12.67888	GCF_902829655.1	7448244	66.9	(16)
33	*Burkholderia ambifaria* BCC1048		√	Environment	42.4779	12.67888	GCF_902829675.1	7513031	66.9	(16)
34	*Burkholderia ambifaria* BCC1066		√	Environment	42.4779	12.67888	GCF_902829905.1	7320233	66.9	(16)
35	*Burkholderia ambifaria* BCC1086		√	Environment	42.4779	12.67888	GCF_902829915.1	7584556	66.9	(16)
36	*Burkholderia ambifaria* BCC1092		√	Environment	42.4779	12.67888	GCF_902829935.1	7384214	66.9	(16)
37	*Burkholderia ambifaria* BCC1093		√	Environment	42.4779	12.67888	GCF_902829945.1	7401226	66.9	(16)
38	*Burkholderia ambifaria* BCC1090		√	Environment	42.4779	12.67888	GCF_902829975.1	7332554	66.9	(16)
39	*Burkholderia ambifaria* BCC1103		√	Environment	42.4779	12.67888	GCF_902829985.1	7398316	66.9	(16)
40	*Burkholderia ambifaria* BCC1080		√	Environment	42.4779	12.67888	GCF_902829995.1	7291234	66.9	(16)
41	*Burkholderia ambifaria* BCC1100		√	Environment	42.4779	12.67888	GCF_902830015.1	7401474	66.9	(16)
42	*Bacillus velezensis* 9912D		√	Sediment sample from the Liaodong	38.17	120.08	GCF_001857985.1	4241576	46.4	

				Bay of the Bohai Sea						(38)
43	*Pseudomonas putida* MX-2	√		Waste water	31.2983	120.5853	GCF_014217705.1	6055976	62.3	(39)
44	*Bacillus cereus* WPySW2	√		Seaweed	27.8362	121.1572	GCF_013112375.1	5750332	35.3	(40)
45	*Paenibacillus donghaensis* KCTC 13049	√		Sediments of the east China sea	38.8593	126.3112	GCF_002192415.1	8542333	49.7	(41)
46	*Klebsiella pneumoniae* J1	√		Harbin taiping sewage treatment plant activated sludge	45.5477	126.9572	GCF_001482345.1	5406866	59.5	(42)
47	*Bacillus licheniformis* HRBL-15TDI7		√	Bean paste	37.5674	126.9827	GCF_001596055.1	4252398	46.3	(43)
48	*Bacillus licheniformis* SCK B11		√	Chili sauce	37.5674	126.9827	GCF_002074075.1	4300706	46.3	(44)
49	*Lactobacillus acidophilus* LA1	√		Fermented dairy products	37.5674	126.9827	GCF_002286215.1	1991195	34.7	(45)
50	*Azoarcus communis* TSPY31	√		Oil-contaminated sediment	37.5674	126.9827	GCF_003111645.1	4572081	62.5	(46)
51	*Azoarcus communis* TSNA42	√		Oil-contaminated sediment	37.5674	126.9827	GCF_003111665.1	4886933	62.5	(46)
52	*Bacillus amyloliquefaciens* MBE1283		√	The south Korean traditional rice wine	35.9203	128.0083	GCF_001483885.1	3979925	46.4	(47)
53	*Bacillus subtilis* BS16045		√	Soybean paste	35.9203	128.0083	GCF_001720505.1	4165121	43.5	(48)
54	*Bacillus velezensis* M75		√	Environment sample from cotton waste for mushroom cultivation	35.9203	128.0083	GCF_001723585.1	4007450	46.4	(49)

55	*Flavobacterium crocinum* HYN0056	√		Freshwater	35.9203	128.0083	GCF_003122385.1	5877431	34	(50)
56	*Bacillus subtilis* SRCM103612		√	The soy sauce	35.9203	128.0083	GCF_004119775.1	4343160	43.5	(51)
57	*Bacillus subtilis* DKU_NT_03	√		Traditional Korean food chung-gook-jang	35.9134	128.2123	GCF_002269195.1	4196031	43.5	(52)
58	*Paraburkholderia megapolitana* LMG 23650	√	√	Aulacomnium palustre	52.3	13.25	GCF_007556815.1	7627590	62.1	(53)
59	*Burkholderia ambifaria* BCC0267		√	Cystic fibrosis patient	-26.121	134.5361	GCF_902829645.1	7358554	66.9	(16)
60	*Burkholderia ambifaria* BCC0250		√	Sputum	-26.121	134.5361	GCF_902829765.1	7356393	66.9	(16)
61	*Bacillus licheniformis* TAB7	√		Compost	35.0324	134.9441	GCF_003595625.1	4440709	46.3	(54)
62	*Bacillus subtilis* HMNig-2		√	Honey the intestinal	25.9162	30.0394	GCF_009497815.1	4178124	43.5	(55)
63	*Arthrobacter* sp. PAMC25564	√		Cryoconite of wurmkogel lengSui rock	-90	50	GCF_004798705.1	4170970	65.6	(56)
64	*Pseudomonas chlororaphis* subsp. *piscium* DSM 21509	√		Freshwater fish	46.5222	8.517857	GCF_003850345.1	7065155	63.1	(57)
65	*Bacillus velezensis* DTU001		√	DTU campus isolation chamber	55.8731	9.956079	GCF_015291865.1	3927210	46.4	(58)
66	*Bacillus amyloliquefaciens* LM2303		√	Chickpea flour	29.6556	91.11806	GCF_001889285.1	3989393	46.4	https://www.ncbi.nlm.nih.gov/genome/annotation_prok/
67	*Bacillus licheniformis* DSM 13		√	Clinical environment	51.265	-2.913	GCF_000008425.1	4222645	46.3	(59)

68	*Pseudomonas aeruginosa* UCBPP-PA14		√	Water and sediment	140.096	36.043	GCF_000014625.1	6537648	66.6	(60)
69	*Bacillus subtilis* TO-A JPC	√		Gastrointestinal tract	35.12	-94.35	GCF_001037985.1	4090708	43.5	(61)
70	*Burkholderia cepacia* NBRC 14074		√	Unkonwn	140.096	36.043	GCF_001528845.1	8486058	67	(62)
71	*Bacillus velezensis* B25		√	Unkonwn	46	130	GCF_001536925.1	3862757	46.4	(63)
72	*Klebsiella oxytoca* AR_0147		√	Mangrove sediment samples	24.08	38.05	GCF_002072655.1	6461325	55.2	(64)
73	*Bacillus subtilis* MBI 600	√	√	Ltd. formulation of the product	22.93	40.83	GCF_005160425.1	4076736	43.5	(65)
74	*Stenotrophomonas maltophilia* KMM349		√	Unidentified sponge	35.63	139.65	GCF_009618035.1	4578300	66.6	(66)
75	*Pseudomonas aeruginosa* LYT4	√	√	Tung oil powder	113	28.14	GCF_012971705.1	6814016	66.6	(67)
76	*Burkholderia ambifaria* BCC0200		√	Maize	39.5509	-101.563	GCF_902829565.1	7627423	66.9	(16)
77	*Burkholderia ambifaria* BCC0191		√	Maize	39.5509	-101.563	GCF_902829465.1	7576661	66.9	(16)
78	*Burkholderia ambifaria* BCC0316		√	Maize	39.5509	-101.563	GCF_902829685.1	7637178	66.9	(16)
79	*Burkholderia contaminans* ZCC	√		Soil at a copper-gold mine	36.1283	108.1333	GCF_007724625.1	9002241	66	(68)
80	*Bacillus velezensis* FJAT-46737		√	Mountain soil	36.1283	108.1333	GCF_008727675.1	3995978	46.4	(69)
81	*Bacillus subtilis* FJAT-4		√	Pig-raising litters from microbial fermentation bed	36.1283	108.1333	GCF_019931715.1	4299840	43.5	(70)

82	*Bacillus velezensis* HN-Q-8		√	Unkonwn	38.8776	115.4588	GCF_009738165.1	4004056	46.4	(71)
83	*Bacillus subtilis* JCL16		√	Huaian aquaculture base to collect soil samples	31.3275	118.8921	GCF_013285325.1	4101682	46.4	(72)
84	*Azoarcus olearius* DQS-4	√		Petroleum contaminated soil	22.6306	120.3068	GCF_001682385.1	4451751	67.8	(73)
85	*Bacillus subtilis* GOT9	√		Gotjawal soil	33.5012	126.5135	GCF_009857115.1	4119195	43.5	(74)
86	*Pseudomonas fluorescens* Pf275		√	Riverside of soilsample	35.9203	128.0083	GCF_003410335.1	6610362	60	(75)
87	*Bacillus velezensis* NKG-1	√	√	Rare dormant volcanic soil	41.4194	128.1961	GCF_002741705.1	4197217	46.4	(76)
88	*Azospirillum* sp. TSH58		√	Termitarium	35.44	140.0033	GCF_003119115.1	7244074	63.6	(77)
89	*Burkholderia pyrrocinia* mHSR5		√	Unknown	51.6935	5.9822633	GCF_003330765.1	8004399	66.8	(78)
90	*Bacillus velezensis* S4		√	Biochar-Treated Soil	39.1857	-75.54574	GCF_011784665.1	4065174	46.4	(79)
91	*Pseudomonas aeruginosa* CR1		√	Unknown	15.5495	80.095774	GCF_003025345.2	6164858	66.2	(80)
92	*Pseudomonas fluorescens* UK4	√		Unknown	57.027	9.9447	GCF_000730425.1	6064456	60	(81)
93	*Pseudomonas fluorescens* Pf0-1		√	Los donggang river to collect soil samples	30	80	GCF_000012445.1	6438405	60	(82)
94	*Achromobacter xylosoxidans* A8		√	Soil contaminated with polychlorinated biphenyls.	miss	miss	GCF_000165835.1	7359146	67.5	(83)
95	*Burkholderia cepacia* ATCC 25416		√	Unknown	miss	miss	GCF_006094315.1	8574389	67	(84)

SA PGPB

Number	Strain	Functions		Distribution			Genome sequence			Reference
		Promote plant growth	Inhibit pathogen infection	Isolation	Latitude	Longitude	Accession number	Size (bp)	GC (%)	
1	*Pseudomonas* sp. Ps634		√	Potato field	54.3036	-0.631565	GCF_902497735.1	6078312	60	(85)
2	*Pseudomonas* sp. Ps652		√	Potato field	54.3036	-0.631565	GCF_902497775.1	6021803	60	(85)
3	*Pseudomonas* sp. Ps685		√	Potato field	54.3036	-0.631565	GCF_902497815.1	5669670	60	(85)
4	*Pseudomonas* sp. Ps689		√	Potato field	54.3036	-0.631565	GCF_902497865.1	6163106	60	(85)
5	*Pseudomonas chlororaphis* subsp. *piscium* DTR133	√		Tomato rhizophere	47.3372	0.757431	GCF_003850425.1	7064618	63.1	(57)
6	*Bacillus subtilis* ATCC 11774	√	√	Potato rhizophere	47.3372	0.757431	GCF_004101945.1	4110138	43.5	(86)
7	*Pseudomonas* sp. Ps624		√	Potato field	52.622	1.216	GCF_902497605.1	6661278	60	(85)
8	*Pseudomonas* sp. Ps619		√	Potato field	52.622	1.216	GCF_902497615.1	7046735	60	(85)
9	*Pseudomonas* sp. Ps682		√	Potato field	52.622	1.216	GCF_902497635.1	6162889	60	(85)
10	*Pseudomonas* sp. Ps623		√	Potato field	52.622	1.216	GCF_902497645.1	5248910	60	(85)
11	*Pseudomonas* sp. Ps664		√	Potato field	52.622	1.216	GCF_902497655.1	6598104	60	(85)
12	*Pseudomonas* sp. Ps681		√	Potato field	52.622	1.216	GCF_902497675.1	6777780	60	(85)
13	*Pseudomonas* sp. Ps683		√	Potato field	52.622	1.216	GCF_902497685.1	6097648	60	(85)
14	*Pseudomonas* sp. Ps631		√	Potato field	52.622	1.216	GCF_902497695.1	5503771	60	(85)
15	*Pseudomonas* sp. Ps663		√	Potato field	52.622	1.216	GCF_902497705.1	6324803	60	(85)
16	*Pseudomonas* sp. Ps659		√	Potato field	52.622	1.216	GCF_902497715.1	6788906	60	(85)
17	*Pseudomonas* sp. Ps647		√	Potato field	52.622	1.216	GCF_902497725.1	6861507	60	(85)
18	*Pseudomonas* sp. Ps655		√	Potato field	52.622	1.216	GCF_902497745.1	6816589	60	(85)
19	*Pseudomonas* sp. Ps684		√	Potato field	52.622	1.216	GCF_902497795.1	6850527	60	(85)

20	*Pseudomonas* sp. Ps687		√	Potato field	52.622	1.216	GCF_902497805.1	6671824	60	(85)
21	*Pseudomonas* sp. Ps706		√	Potato field	52.622	1.216	GCF_902497835.1	6520755	60	(85)
22	*Pseudomonas* sp. Ps712		√	Potato field	52.622	1.216	GCF_902497845.1	6642968	60	(85)
23	*Pseudomonas* sp. Ps833		√	Potato field	52.622	1.216	GCF_902497855.1	7408731	60	(85)
24	*Pseudomonas* sp. Ps720		√	Potato field	52.622	1.216	GCF_902497905.1	7079388	60	(85)
25	*Pseudomonas* sp. Ps718		√	Potato field	52.622	1.216	GCF_902497925.1	6292867	60	(85)
26	*Pseudomonas* sp. Ps708		√	Potato field	52.622	1.216	GCF_902497935.1	6640566	60	(85)
27	*Pseudomonas* sp. Ps834		√	Potato field	52.622	1.216	GCF_902497945.1	6296691	60	(85)
28	*Pseudomonas* sp. Ps887		√	Potato field	52.622	1.216	GCF_902497955.1	6067054	60	(85)
29	*Pseudomonas* sp. Ps874		√	Potato field	52.622	1.216	GCF_902497965.1	7098510	60	(85)
30	*Pseudomonas* sp. Ps854		√	Potato field	52.622	1.216	GCF_902497975.1	6243141	60	(85)
31	*Pseudomonas* sp. Ps898		√	Potato field	52.622	1.216	GCF_902498005.1	6054456	60	(85)
32	*Pseudomonas* sp. Ps838		√	Potato field	52.622	1.216	GCF_902498015.1	6869354	60	(85)
33	*Pseudomonas* sp. Ps880		√	Potato field	52.622	1.216	GCF_902498025.1	6871429	60	(85)
34	*Pseudomonas* sp. Ps870		√	Potato field	52.622	1.216	GCF_902498035.1	7101195	60	(85)
35	*Pseudomonas* sp. Ps843		√	Potato field	52.622	1.216	GCF_902498045.1	6512546	60	(85)
36	*Pseudomonas* sp. Ps858		√	Potato field	52.622	1.216	GCF_902498065.1	6211017	60	(85)
37	*Pseudomonas* sp. Ps903		√	Potato field	52.622	1.216	GCF_902498075.1	6833382	60	(85)
38	*Pseudomonas* sp. Ps896		√	Potato field	52.622	1.216	GCF_902498085.1	6410764	60	(85)
39	*Pseudomonas* sp. Ps893		√	Potato field	52.622	1.216	GCF_902498095.1	6376713	60	(85)
40	*Pseudomonas* sp. Ps876		√	Potato field	52.622	1.216	GCF_902498115.1	5823752	60	(85)
41	*Pseudomonas* sp. Ps865		√	Potato field	52.622	1.216	GCF_902498145.1	6153338	60	(85)

42	*Pseudomonas* sp. Ps922		√	Potato field	52.622	1.216	GCF_902498155.1	6450032	60	(85)
43	*Pseudomonas* sp. Ps906		√	Potato field	52.622	1.216	GCF_902498165.1	5914562	60	(85)
44	*Pseudomonas* sp. Ps907		√	Potato field	52.622	1.216	GCF_902498175.1	6013679	60	(85)
45	*Pseudomonas* sp. Ps910		√	Potato field	52.622	1.216	GCF_902498185.1	4854699	60	(85)
46	*Pseudomonas* sp. Ps914		√	Potato field	52.622	1.216	GCF_902498205.1	7570449	60	(85)
47	*Pseudomonas* sp. Ps941		√	Potato field	52.622	1.216	GCF_902498215.1	6683563	60	(85)
48	*Pseudomonas* sp. Ps934		√	Potato field	52.622	1.216	GCF_902498225.1	6543376	60	(85)
49	*Pseudomonas* sp. Ps925		√	Potato field	52.622	1.216	GCF_902498235.1	6962445	60	(85)
50	*Pseudomonas* sp. Ps928		√	Potato field	52.622	1.216	GCF_902498245.1	7454519	60	(85)
51	*Pseudomonas* sp. Ps938		√	Potato field	52.622	1.216	GCF_902498255.1	6291618	60	(85)
52	*Pseudomonas* sp. Ps943		√	Potato field	52.622	1.216	GCF_902498265.1	6485274	60	(85)
53	*Pseudomonas* sp. Ps947		√	Potato field	52.622	1.216	GCF_902498275.1	6749079	60	(85)
54	*Pseudomonas* sp. Ps627		√	Potato field	52.622	1.216	GCF_911283435.1	4968056	60	(85)
55	*Bdellovibrio bacteriovorus* HD100		√	Nepenthes soil	39.5509	-101.5633	GCF_000196175.1	3782950	50.7	(87)
56	*Burkholderia contaminans* MS14		√	The lawn soil	39.5509	-101.5633	GCF_001029145.1	8509249	66	(88)
57	*Pseudomonas fluorescens* MS82		√	Soybean rhizophere	39.5509	-101.5633	GCF_003055645.1	6207556	60	(89)
58	*Azospirillum brasilense* Cd	√		Mangrove roots	39.5509	-101.5633	GCF_008274965.1	7441087	68.3	(90)
59	*Burkholderia ambifaria* BCC1224		√	Maize rhizophere	39.5509	-101.5633	GCF_902829395.1	8458324	66.9	(16)
60	*Burkholderia ambifaria* BCC0191		√	Maize	39.5509	-101.5633	GCF_902829465.1	7576661	66.9	(16)
61	*Burkholderia ambifaria*		√	Maize rhizophere	39.5509	-101.5633	GCF_902829475.1	7599477	66.9	(16)

	BCC1258									
62	*Burkholderia ambifaria* BCC0316		√	Maize	39.5509	-101.5633	GCF_902829685.1	7637178	66.9	(16)
63	*Burkholderia ambifaria* BCC0284		√	Corn roots	39.5509	-101.5633	GCF_902829725.1	7465452	66.9	(16)
64	*Burkholderia ambifaria* BCC0338		√	Corn roots	39.5509	-101.5633	GCF_902829825.1	7454022	66.9	(16)
65	*Burkholderia ambifaria* BCC1229		√	Maize rhizophere	39.5509	-101.5633	GCF_902830045.1	7512128	66.9	(16)
66	*Burkholderia ambifaria* BCC1237		√	Maize rhizophere	39.5509	-101.5633	GCF_902830055.1	7343332	66.9	(16)
67	*Burkholderia ambifaria* BCC1228		√	Maize rhizophere	39.5509	-101.5633	GCF_902830065.1	7379185	66.9	(16)
68	*Burkholderia ambifaria* BCC1236		√	Maize rhizophere	39.5509	-101.5633	GCF_902830095.1	7626299	66.9	(16)
69	*Burkholderia ambifaria* BCC1252		√	Maize rhizophere	39.5509	-101.5633	GCF_902830125.1	7418533	66.9	(16)
70	*Burkholderia ambifaria* BCC1270		√	Maize rhizophere	39.5509	-101.5633	GCF_902830155.1	7596866	66.9	(16)
71	*Burkholderia ambifaria* BCC1256		√	Maize rhizophere	39.5509	-101.5633	GCF_902830165.1	7539062	66.9	(16)
72	*Burkholderia ambifaria* BCC1241		√	Maize rhizophere	39.5509	-101.5633	GCF_902830175.1	7682361	66.9	(16)
73	*Burkholderia ambifaria* BCC1246		√	Maize rhizophere	39.5509	-101.5633	GCF_902830205.1	7482117	66.9	(16)
74	*Burkholderia ambifaria* BCC1259		√	Maize rhizophere	39.5509	-101.5633	GCF_902830225.1	7626413	66.9	(16)
75	*Rhizobium* sp. ACO-34A	√		Tequila rhizophere	25.1257	-104.5436	GCF_002600635.1	6284736	60.8873	(91)
76	*Bacillus halotolerans*	√		The maize of rhizophere	40.44	105.12	GCF_004006435.1	4154245	43.8	(92)

	ZB201702									
77	*Bacillus cereus* CF4-51		√	Sunflower of rhizophere	40.44	105.12	GCF_018884185.1	5993925	35.3	https://www.ncbi.nlm.nih.gov/genome/annotation_prok/
78	*Bacillus cereus* A22		√	Coffee root	20.6845	105.85199	GCF_006349715.2	5885310	35.3	(93)
79	*Bacillus cereus* A24		√	Black pepper root	20.6845	105.85199	GCF_006349735.2	5751928	35.3	(93)
80	*Bacillus cereus* HD2.4		√	Tomato root	20.6845	105.85199	GCF_013284455.2	5739795	46.4	(93)
81	*Bacillus cereus* HD1.4B		√	Tomato root	20.6845	105.85199	GCF_013284505.2	5740353	46.4	(93)
82	*Bacillus subtilis* GQJK2	√	√	Chinese wolfberry rhizosphere	38.4886	106.23111	GCF_002072735.1	4072961	43.5	https://www.ncbi.nlm.nih.gov/genome/annotation_prok/
83	*Paenibacillus polymyxa* ZF129		√	Potato rhizophere	36.0046	106.28524	GCF_006274405.1	5820553	45.4	(94)
84	*Burkholderia cenocepacia* CR318	√		Starch corn of root	55.6854	-106.457	GCF_002007585.1	7664894	67	(95)
85	*Bacillus velezensis* 9D-6	√	√	Potato rhizophere	55.6854	-106.457	GCF_002105595.1	3963726	46.4	(96)
86	*Bacillus velezensis* 1B-23		√	Potato rhizophere	55.6854	-106.457	GCF_003854915.1	4141063	46.4	(97)
87	*Pseudomonas fluorescens* LBUM677	√		Strawberry rhizophere	55.6854	-106.457	GCF_004683905.1	6140320	60	(98)
88	*Bacillus velezensis* LBUM1082		√	Strawberry rhizophere	55.6854	-106.457	GCF_016065635.1	3933785	46.4	(1)
89	*Brevibacillus brevis* DZQ7		√	Tobacco rhizophere	26.6453	106.62333	GCF_001039275.2	6443936	47.4	(99)
90	*Bacillus velezensis* GUMT319		√	Tobacco rhizophere	26.6453	106.62333	GCF_016766595.1	3940023	46.4	(100)
91	*Paenibacillus polymyxa* YC0136	√	√	Tobacco rhizophere	27.5363	106.82922	GCF_001874405.2	5621728	45.4	(101)
92	*Paenibacillus polymyxa*	√	√	Tobacco rhizophere	27.5363	106.82922	GCF_001874425.3	6126117	45.4	(102)

	YC0573									
93	*Bacillus amyloliquefaciens* Ba13	√	√	Tomato rhizophere	34.2722	108.08455	GCF_018223605.1	3861210	46.4	(103)
94	*Bacillus cereus* AR156	√		Arabidopsis thaliana rhizophere	36.1283	108.13325	GCF_001880305.1	5671798	35.3	(104)
95	*Arthrobacter* sp. PGP41	√		Solanum nigrum rhizosphere	36.1283	108.13325	GCF_002953935.1	4270237	65.6	(105)
96	*Bacillus velezensis* DSYZ	√	√	Garlic rhizosphere	36.1283	108.13325	GCF_003285085.1	4321463	46.4	(106)
97	*Serratia proteamaculans* 336X		√	Wheat rhizophere	36.1283	108.13325	GCF_009660185.1	5593263	55.1	(107)
98	*Pseudomonas chlororaphis* subsp. *aurantiaca* zm-1		√	Anemarrhena asphodeloides rhizophere	36.1283	108.13325	GCF_010092525.1	6861331	63.1	(108)
99	*Rhizobium oryzihabitans* M15	√		Rice root	36.1283	108.13325	GCF_010669145.1	5858741	59.4	(109)
100	*Achromobacter xylosoxidans* GD03	√		Paddy soil and rice rhizophere	36.1283	108.13325	GCF_014490035.1	6761376	67.5	(110)
101	*Bacillus velezensis* GS-1		√	Vegetable soil	36.1283	108.13325	GCF_019449395.1	4030799	46.4	(111)
102	*Cellulomonas iranensis* ZJW-6		√	Paddy	36.1283	108.13325	GCF_020412445.1	4046676	75.3	(112)
103	*Bacillus amyloliquefaciens* GKT04		√	Banana root	22.45	108.26	GCF_019396925.1	4149690	46.4	(113)
104	*Pantoea ananatis* NN08200	√		Sugarcane root	22.7812	108.27331	GCF_004028255.1	5176640	53.7	(114)
105	*Pseudomonas aeruginosa* B18	√	√	Sugarcane root	22.7812	108.27331	GCF_013395035.1	6490014	66.6	(115)
106	*Bacillus velezensis* LS69		√	Rice field	30.291	108.93655	GCF_001687745.1	3917761	46.4	(116)
107	*Klebsiella variicola* DX120E	√		Sugarcane endophyte	22.6364	110.165	GCF_000812205.2	5718434	57.5	(117)
108	*Bacillus subtilis* S-16		√	Sunflower of rhizophere	40.51	110.6	GCF_018884165.1	4209504	43.5	(118)
109	*Brevibacillus brevis* X23		√	Tobacco rhizophere	28.2559	112.98626	GCF_000296715.2	6643437	47..4	(119)

110	*Klebsiella pneumoniae* NFYY0065	√		Cashew endophyte	23.1278	113.26389	GCF_004137665.1	5235159	57.5	(120)
111	*Paenibacillus kribbensis* PS04		√	Insecticidal botanical garden soil	23.1579	113.27324	GCF_013394225.1	5737770	46.8	(121)
112	*Bacillus subtilis* TR21		√	Caulis dendrobii endophyte	23.1579	113.27324	GCF_015535655.1	4105857	43.5	(6)
113	*Bacillus velezensis* LDO2	√	√	Peanut root	32.62	114.38	GCF_003073455.1	3947271	46.4	(122)
114	*Bacillus velezensis* YB-130		√	Wheat field	33.79	114.5	GCF_014358035.1	3980767	46.4	(123)
115	*Bacillus subtilis* J-5		√	Tomato rhizophere	38.0365	114.53952	GCF_001889385.1	4117900	43.5	(124)
116	*Paenibacillus polymyxa* HY96-2		√	Tomato rhizophere	28.5454	115.94422	GCF_002893885.1	5745779	46.4	(125)
117	*Pantoea* sp. CCBC3-3-1		√	Cotinus coggygria endophyte	39.59	116.1	GCF_007981265.1	5159767	55	(126)
118	*Pantoea ananatis* YJ76	√		Rice endophyte	39.26	116.13	GCF_002224585.2	5146480	53.7	(127)
119	*Bacillus velezensis* 19573-3		√	Tomato rhizophere	39.26	116.13	GCF_019551675.1	3990203	46.4	(128)
120	*Bacillus velezensis* ZF2		√	Cucumber endophyte	39.57	116.19	GCF_003555525.1	3929773	46.4	(129)
121	*Rahnella aquatilis* HX2 HX2	√	√	Grapes	40.2208	116.23128	GCF_000255535.1	5656799	52.1	(130)
122	*Bacillus velezensis* Lzh-a42	√	√	Tomato rhizophere	37.4508	116.29941	GCF_002844125.1	4246605	46.4	(131)
123	*Klebsiella oxytoca* P620	√		Cucumber rhizophere	35.4082	116.59649	GCF_009707385.1	5394761	55.2	https://www.ncbi.nlm.nih.gov/genome/annotation_prok/
124	*Brevibacillus laterosporus* ZQ2		√	Apple rhizophere	36.2014	117.0875	GCF_002865525.1	5207640	40.7	https://www.ncbi.nlm.nih.gov/genome/annotation_prok/
125	*Bacillus velezensis* PEBA20		√	Poplar endophyte	36.2014	117.0875	GCF_016859395.1	4284837	46.4	(132)
126	*Pseudomonas*		√	Sweet potato	34.2882	117.18554	GCF_016413685.1	6757898	63.1	(133)

	chlororaphis subsp. *aureofaciens* SPS-41			rhizophere						
127	*Pseudomonas aeruginosa* L10	√		Reed root	37.4273	118.02279	GCF_002223805.1	6661962	66.6	(134)
128	*Serratia ureilytica* T6		√	Pepper endophyte	27.0227	118.30498	GCF_017309605.1	5102941	59.7	(135)
129	*Bacillus velezensis* S3-1		√	Cucumber rhizophere	39.2731	118.46023	GCF_001685645.1	3929772	46.4	(136)
130	*Bacillus subtilis* Bs-916		√	Rice paddy soil	31.3275	118.8921	GCF_000772205.1	3981674	43.5	(137)
131	*Bacillus cereus* TG1-6	√		Rice rhizophere	26.0747	119.29667	GCF_003013315.1	5431893	35.3	(138)
132	*Klebsiella variicola* GN02	√		The root of remembranous milk vetch	26.0747	119.29667	GCF_014217415.1	5599366	57.5	https://www.ncbi.nlm.nih.gov/genome/annotation_prok/
133	*Paenibacillus polymyxa* ZF197		√	Potato rhizophere	36.3826	119.75568	GCF_007858415.1	5539234	45.4	(94)
134	*Bacillus subtilis* MB9_B4		√	Mushroom	55.8439	12.42477	GCF_009662155.1	4105407	43.5	(139)
135	*Bacillus subtilis* MB8_B10		√	Mushroom	55.8439	12.42477	GCF_009662195.1	4225362	43.5	(139)
136	*Bacillus subtilis* MB8_B7		√	Mushroom	55.8439	12.42477	GCF_009662215.1	4275601	43.5	(139)
137	*Bacillus subtilis* MB8_B1		√	Mushroom	55.8439	12.42477	GCF_009662255.1	4221278	43.5	(139)
138	*Burkholderia ambifaria* BCC0423		√	Maize	42.4779	12.678879	GCF_902829805.1	7472042	66.9	(16)
139	*Burkholderia ambifaria* BCC1072		√	Maize	42.4779	12.678879	GCF_902830005.1	7442983	66.9	(16)
140	*Bacillus velezensis* CGMCC 11640		√	Bamboo forest soil	30.2853	120.15444	GCF_002968415.1	4385679	46.4	(140)
141	*Pseudomonas aeruginosa* F9676		√	Rice seed	30.2084	120.21201	GCF_001077475.1	6368008	66.6	(141)
142	*Burkholderia* sp. JP2-270		√	Rice rhizophere	30.2084	120.21201	GCF_003233675.1	8925310	66.6211	(142)
143	*Pseudomonas fluorescens* 2P24		√	Wheat	36.0667	120.38333	GCF_002865505.1	6610571	60	(143)
144	*Burkholderia*	√	√	Vetiver grass	24.1432	120.67988	GCF_000705535.2	8022336	67.1	(144)

	seminalis 869T2			root						
145	*Burkholderia gladioli* BBB-01		√	Rice shoots	24.1432	120.67988	GCF_016698705.1	8201484	68	(20)
146	*Pseudomonas chlororaphis* subsp. *Aurantiaca* JD37	√	√	Potato rhizophere	31.47	121.28	GCF_000761195.1	6702062	63.1	(145)
147	*Pseudomonas aeruginosa* YY322		√	Saffron rhizophere	31.47	121.28	GCF_019466145.1	6382345	66.6	(146)
148	*Klebsiella pneumoniae* ED2		√	Siphon	23.593	121.70033	GCF_001708245.1	5412960	57.5	(147)
149	*Bacillus cereus* C1L	√		Maize rhizophere	23.593	121.70033	GCF_002224345.1	6047547	46.4	(148)
150	*Rhizobium pusense* IRBG74	√		Arabidopsis thaliana root	12.5157	122.26834	GCF_000499645.1	5464982	59.3	(149)
151	*Klebsiella pneumoniae* 2N3	√	√	Corn	41.8052	123.46987	GCF_002850275.3	5319547	57.5	(150)
152	*Bacillus velezensis* A2	√	√	Farmland soil	41.8052	123.46987	GCF_013201135.1	3929218	46.4	(151)
153	*Flavobacterium anhuiense* GSE09		√	Cucumber root	38.8593	126.31115	GCF_001705175.1	5109718	34.3	(152)
154	*Pseudomonas putida* JBC17	√		Strawberry rhizophere	35.7175	127.153	GCF_003228315.1	6845198	62.3	(153)
155	*Bacillus velezensis* G341		√	Korean ginseng root	37.4795	127.19422	GCF_001023595.1	4009746	46.4	(154)
156	*Paenibacillus yonginensis* DCY84	√		Forest soil	37.4795	127.19422	GCF_001685395.1	4985901	51	(155)
157	*Bacillus velezensis* CBMB205	√		Rice rhizophere	37.4795	127.19422	GCF_002117165.1	3929792	46.4	(156)
158	*Bacillus velezensis* GH1-13	√	√	Rice paddy soil	40.1914	127.55757	GCF_002005345.1	4143608	46.4	(157)
159	*Burkholderia* sp. KJ006		√	Rice root	35.9203	128.0083	GCF_000262695.1	6629912	66.6211	(158)
160	*Burkholderia cepacia* JBK9		√	Plant rhizophere	35.9203	128.0083	GCF_000701165.1	8481212	67	(84)
161	*Serratia marcescens* RSC-14	√		Morel root	35.9203	128.0083	GCF_001280365.1	5127030	59.6	(159)
162	*Bacillus velezensis* DR-08		√	Pepper rhizophere	35.9203	128.0083	GCF_003045165.1	3929794	46.4	(160)

163	*Brevibacillus brevis* HK544		√	Forest soil	35.9203	128.0083	GCF_007725005.1	6486246	47.4	(161)
164	*Serratia rhizosphaerae* KUDC3025		√	Potato endophyte	35.9203	128.0083	GCF_009817885.1	5098052	58	(162)
165	*Pseudomonas fluorescens* DR397	√		Soybean rhizophere	35.9203	128.0083	GCF_010448615.1	6418441	60	(163)
166	*Bacillus velezensis* AK-0		√	Ginseng rhizophere	35.9203	128.0083	GCF_014706595.1	3969447	46.4	(164)
167	*Bacillus licheniformis* CP6		√	Cotton field	35.9203	128.0083	GCF_017338795.1	4316909	46.3	(165)
168	*Bacillus velezensis* T20E-257	√	√	Tomato root	36.7176	128.499	GCF_002205715.1	3900066	46.4	(166)
169	*Serratia plymuthica* 3Rp8		√	Rapeseed rhizosphere	52.3	13.25	GCF_001663115.1	5546041	56	(167)
170	*Pseudomonas chlororaphis* subsp. *Aurantiaca* CW2	√		Rapeseed rhizosphere	52.3	13.25	GCF_003851225.1	6925198	63.1	(168)
171	*Azoarcus* sp. KH32C	√		Paddy field	32.832	130.7173	GCF_000349945.1	5818755	65.9	(169)
172	*Pseudomonas chlororaphis* subsp. *aurantiaca* StFRB508		√	Potato rhizophere	31.21	130.78	GCF_002355875.1	6997933	63.1	(170)
173	*Rhizobium leguminosarum* bv. *trifolii* CC275e	√		White clover root	-26.121	134.53605	GCF_000769405.2	7118658	60.7	(171)
174	*Paraburkholderia* sp. SOS3	√		Tomato rhizophere	-26.121	134.53605	GCF_001922345.1	7502907	63.1	(172)
175	*Bacillus velezensis* KOF112		√	Grape endophyte	35.6803	138.56922	GCF_018406485.1	3929792	46.4	(173)
176	*Azospirillum* sp. TSH100	√		Paddy soil	38.5674	140.70243	GCF_004923295.1	7166382	63.6	(174)
177	*Azospirillum* sp. TSA2s	√		Paddy soil	38.5674	140.70243	GCF_004923315.1	8102478	63.6	(174)
178	*Arthrobacter* sp. UKPF54-2	√	√	Paddy soil	38.5674	140.70243	GCF_007858535.1	3517818	65.6	(175)
179	*Bradyrhizobium japonicum* J5	√		Soybean nodule	43.3345	143.31485	GCF_001887695.1	10138651	63.7	(176)

180	*Pseudomonas chlororaphis* subsp. *aurantiaca* M71	√		Tomato rhizophere	40.7872	15.052131	GCF_003851265.1	6807002	63.1	(168)
181	*Rhizobium leguminosarum* bv. *trifolii* WSM1325	√		Trifolium nodule	34.5112	17.682944	GCF_000023185.1	7418122	60.7	(177)
182	*Bacillus subtilis* SZMC 6179J		√	Tomato rhizophere	46.6828	19.964209	GCF_001604995.1	4195195	43.5	(178)
183	*Pseudomonas chlororaphis* subsp. *aureofaciens* P2	√		Potato rhizophere	27.4709	2.6859134	GCF_003851365.1	7203062	63.1	(168)
184	*Pseudomonas chlororaphis* subsp. *aurantiaca* M12	√		Maize rhizophere	43.6114	21.951117	GCF_003851165.1	6730980	63.1	(168)
185	*Pseudomonas chlororaphis* subsp. *aurantiaca* Q16	√		Alfalfa rhizophere	43.6114	21.951117	GCF_003851345.1	6928549	63.1	(168)
186	*Pseudomonas chlororaphis* subsp. *aureofaciens* C50	√		Maize rhizophere	43.6114	21.951117	GCF_003851385.1	6741275	63.1	(168)
187	*Bacillus amyloliquefaciens* S499	√		Tomato rhizophere	-0.8658	23.405154	GCF_001586105.1	3935930	46.4	(179)
188	*Pseudomonas chlororaphis* subsp. *piscium* ToZa7	√		Tomato rhizophere	38.9282	24.600979	GCF_003850585.1	7015602	63.1	(168)
189	*Pseudomonas chlororaphis* subsp. *piscium* PCL1391	√		Tomato rhizophere	39.1005	-3.160542	GCF_003850445.1	6870622	63.1	(168)
190	*Pseudomonas chlororaphis* subsp. *piscium* PCL1607	√		Avocado rhizosphere	39.1005	-3.160542	GCF_003850465.1	6913645	63.1	(168)
191	*Azoarcus* sp. CIB	√		Rice rhizophere	40.2097	-3.270926	GCF_001190925.1	5257030	65.5	(180)
192	*Burkholderia ambifaria*		√	Maize	39.5509	-101.563	GCF_902829565.1	7627423	66.9	

	BCC0200									(16)
193	*Bacillus velezensis* B4-7		√	Citrus rhizophere	109.488	30.27217	GCF_019603335.1	3926832	46.4	(181)
194	*Pseudomonas chlororaphis* subsp. *aurantiaca* 449	√		Maize rhizophere	50.2578	30.616895	GCF_003851205.1	6962068	63.1	(168)
195	*Pseudomonas chlororaphis* subsp. *aurantiaca* 464	√		Beet rhizophere	50.2578	30.616895	GCF_003851805.1	6964452	63.1	(168)
196	*Klebsiella* sp. MPUS7	√		Eggplant rhizophere	-7.3652	34.296354	GCF_009905335.1	5823634	57.1	(182)
197	*Cellulomonas* sp. JZ18	√		The cuckoo grass	16.8665	42.644842	GCF_009720485.1	4043325	73.5	(182)
198	*Paenibacillus* sp. JZ16	√		Zygophyllum simplex	16.8665	42.644842	GCF_015326965.1	7421843	47.2	(182)
199	*Paraburkholderia graminis* PHS1		√	Hoeven soil	51.6935	5.9822633	GCF_003330785.1	7508450	62.5	(78)
200	*Paraburkholderia terricola* mHS1		√	Broccoli	51.6935	5.9822633	GCF_003330825.1	7118039	63.8	(78)
201	*Pseudomonas chlororaphis* subsp. *piscium* SLPH10	√		Arabidopsis	51.6935	5.9822633	GCF_003850405.1	7227643	63.1	(168)
202	*Paenibacillus riograndensis* SBR5	√	√	Wheat rhizophere	-9.8275	-52.88404	GCF_000981585.1	7919576	51	(183)
203	*Pantoea agglomerans* 33.1	√		The eucalyptus	-9.8275	-52.88404	GCF_020149765.1	4844324	55.4	(184)
204	*Pseudomonas chlororaphis* subsp. *piscium* ChPhzS135	√		Solanum rhizophere	44.0376	6.120243	GCF_003850485.1	6940016	63.1	(168)
205	*Pseudomonas chlororaphis* subsp. *piscium* ChPhzS140	√		Solanum rhizophere	44.0376	6.120243	GCF_003850505.1	7074317	63.1	(168)
206	*Pseudomonas chlororaphis* subsp. *piscium* ChPhzTR44	√		Tomato rhizophere	44.0376	6.120243	GCF_003850525.1	6878216	63.1	(168)
207	*Pseudomonas chlororaphis*	√		Solanum rhizophere	44.0376	6.120243	GCF_003851445.1	6889553	63.1	(168)

	subsp. *aureofaciens* ChPhzS24									
208	*Pseudomonas chlororaphis* subsp. *aureofaciens* ChPhzTR36	√	√	Tomato rhizophere	44.0376	6.120243	GCF_003851865.1	6934077	63.1	(185)
209	*Pseudomonas chlororaphis* subsp. *aureofaciens* ChPhzTR39	√		Tomato rhizophere	44.0376	6.120243	GCF_003851925.1	7046099	63.1	(168)
210	*Pseudomonas chlororaphis* subsp. *aureofaciens* ChPhzTR18	√		Tomato rhizophere	44.0376	6.120243	GCF_003851955.1	6873200	63.1	(168)
211	*Pseudomonas chlororaphis* subsp. *aureofaciens* ChPhzTR38	√		Tomato rhizophere	44.0376	6.120243	GCF_003852005.1	6947716	63.1	(168)
212	*Pseudomonas fluorescens* L321	√		Miscanthus giganteus	52.51	6.54	GCF_001708445.1	6641144	60	(186)
213	*Pseudomonas fluorescens* L111	√		Miscanthus giganteus	52.51	6.54	GCF_001708465.1	6606606	60	(186)
214	*Azospirillum brasilense* Az39	√		Wheat root	-40.378	-67.30752	GCF_000632475.1	7391279	68.4	(187)
215	*Bradyrhizobium japonicum* E109	√		Soybean root	-40.378	-67.30752	GCF_000807315.1	9224208	63.7	(188)
216	*Burkholderia ambifaria* T16		√	Barley rhizophere	-40.378	-67.30752	GCF_011392275.1	7358950	66.9	(189)
217	*Pseudomonas chlororaphis* subsp. *aureofaciens* 66	√		Alfalfa rhizophere	41.2037	69.269616	GCF_003851405.1	6797278	63.1	(168)
218	*Azoarcus olearius* BH72	√		Grass	28.7757	70.240787	GCF_000061505.1	4376040	67.8	(190)
219	*Pseudomonas chlororaphis* subsp. *aurantiaca* ARS 38	√	√	Cotton rhizophere	28.7757	70.240787	GCF_009362955.1	6615046	63.1	(191)
220	*Azospirillum*	√		Grasses	20.1295	73.969474	GCF_005222145.1	7134170	68.3	(192)

	brasilense MTCC4038			rhizophere						
221	*Azospirillum brasilense* MTCC4039	√		Grasses rhizophere	20.1295	73.969474	GCF_005222205.1	7196248	68.3	(192)
222	*Serratia marcescens* N4-5		√	Plant rhizophere	40.0051	-74.74002	GCF_003355135.1	5085562	59.6	(193)
223	*Paraburkholderia xenovorans* LB400		√	Ginseng	42.8432	-75.38393	GCF_000013645.1	9731138	62.8	(194)
224	*Klebsiella pneumoniae* CAV1042	√		Grasses rhizophere	37.2639	-78.71384	GCF_001908515.1	5752260	57.5	(195)
225	*Azospirillum brasilense* MTCC4035	√		Grasses rhizophere	19.8512	79.764622	GCF_005222165.1	7928656	68.4	(192)
226	*Pseudomonas veronii* R02		√	Picrorhiza kurroa plantlets	47.4817	8.2175472	GCF_002028325.1	6852809	61.1	(196)
227	*Bacillus subtilis* SP1		√	Apple rhizophere	46.5222	8.5178565	GCF_013388335.1	4215613	46.4	(197)
228	*Bradyrhizobium diazoefficiens* USDA 110	√		Soybean root	27.6648	-81.51576	GCF_000011365.1	9105828	64.1	(198)
229	*Klebsiella* sp. LTGPAF-6F	√		Alfalfa rhizophere	43.8258	87.61694	GCF_001753185.1	6790399	57.1	(199)
230	*Bacillus velezensis* AL7		√	Cotton	43.8258	87.61694	GCF_009663035.1	3894709	46.4	(200)
231	*Bacillus velezensis* HAB-2		√	Cotton	43.8258	87.61694	GCF_014211995.1	3894648	46.4	(201)
232	*Burkholderia* sp. MS389		√	Soybean	32.7553	-89.82581	GCF_016899425.1	7746250	66.6211	(202)
233	*Burkholderia* sp. MS455		√	Soybean	32.7553	-89.82581	GCF_016899445.1	8055789	66.6211	(203)
234	*Rhizobium bangladeshense* BLR175	√		Lentil root nodules	24.0056	90.293841	GCF_017357245.1	6348611	61.1	(204)
235	*Bacillus velezensis* FZB42	√	√	Beet rhizophere	52.53	13.03	GCF_000015785.2	3918596	46.4	(205)
236	*Paraburkholderia phytofirmans* PsJN	√		Onion root	30	80	GCF_000020125.1	8214658	62.6	(206)
237	*Bradyrhizobium* sp. ORS278	√	√	Paddy	52.02	0.001	GCF_000026145.1	7456587	63.5	(207)
238	*Paenibacillus*	√	√	Barley	miss	miss	GCF_000146875.3	5394883	45.5	(208)

	polymyxa E681			rhizophere						
239	*Pantoea vagans* C9-1		√	Apple rhizophere	miss	miss	GCF_000148935.1	4888338	55.6	(209)
240	*Paenibacillus polymyxa* SC2	√	√	Capsicum rhizophere	miss	miss	GCF_000164985.3	6238510	45.4	(210)
241	*Serratia plymuthica* 4Rx13		√	Brassica napus rhizosphere	miss	miss	GCF_000176835.2	5403731	56	(211)
242	*Pseudomonas putida* BIRD-1	√		Plant rhizophere	miss	miss	GCF_000183645.1	5731541	62.3	(212)
243	*Serratia* sp. AS12		√	Rapeseed rhizosphere	miss	miss	GCF_000214195.1	5443009	59.3	(213)
244	*Serratia* sp. AS13	√	√	Rapeseed rhizosphere	miss	miss	GCF_000214805.1	5442549	59.3	(214)
245	*Pseudomonas aeruginosa* M18		√	Sweet melon rhizophere	miss	miss	GCF_000226155.1	6327754	66.6	(215)
246	*Paenibacillus polymyxa* M1		√	Plant rhizophere	miss	miss	GCF_000237325.1	6231122	45.4	(216)
247	*Azospirillum lipoferum* 4B	√		Rice root	miss	miss	GCF_000283655.1	6846400	67.3	(217)
248	*Bradyrhizobium oligotrophicum* S58	√		Paddy soil	miss	miss	GCF_000344805.1	8264165	65.1	(218)
249	*Paenibacillus polymyxa* CR1	√	√	Corn roots	miss	miss	GCF_000507205.3	6019882	45.4	(219)
250	*Paenibacillus polymyxa* SQR-21	√	√	Watermelon rhizophere	miss	miss	GCF_000597985.1	5828436	45.4	(220)
251	*Bacillus velezensis* SQR9	√	√	Cucumber rhizophere	miss	miss	GCF_000685725.1	4117023	46.4	(221)
252	*Serratia* sp. FS14		√	Large-headed atractylodes	miss	miss	GCF_000695995.1	5249875	59.3	(222)
253	*Bacillus subtilis* BS49		√	The tea rhizophere	miss	miss	GCF_000953615.1	4251652	43.5	(223)
254	*Bacillus subtilis* NCIB 3610		√	The mango tree soil	miss	miss	GCF_002055965.1	4299822	43.5	(224)
255	*Paraburkholderia unamae* SCZa-39	√		Zea rhizophere	miss	miss	GCF_003096875.1	10196615	64.9	(225)
256	*Paraburkholderia silvatlantica* SRCL-318	√		Zea rhizophere	miss	miss	GCF_003217075.1	8087491	64.5	(226)
257	*Serratia quinivorans* PKL:12	√		Picrorhiza kurroa plantlets	miss	miss	GCF_004684265.1	5292949	55.1	(227)
258	*Azospirillum brasilense* Sp7	√		Wheat rhizophere	miss	miss	GCF_007827425.1	6587527	68.3	(228)

259	*Bradyrhizobium elkanii* USDA 61	√		Soybean root	miss	miss	GCF_012871055.1	9649995	63.7	(229)
260	*Rhizobium ruizarguesonis* NZLR24	√		Lentils	miss	miss	GCF_017357285.1	7811507	61.1	(230)
261	*Burkholderia cenocepacia* TAtl-371		√	Tomato rhizophere	miss	miss	GCF_900100915.1	7496106	67	(231)
262	*Acinetobacter calcoaceticus* str. 2117	√		Alfalfa and Chickpea plants	miss	miss	GCF_900520355.1	3934114	38.7	(232)
263	*Rahnella aquatilis* ZF7	√	√	Cherry Rhizophere	39.59	116.1	GCF_003573465.1	5536721	52.1	(233)
264	*Methylobacterium* sp. 2A	√		Solanum tuberosum rhizophere	-34.558	-58.459	GCF_009806555.1	6395352	69.5	(234)
265	*Burkholderia ambifaria* BCC0207		√	Pea rhizophere	42.4779	12.678879	GCF_902829835.1	7305861	66.9	(16)
266	*Bacillus subtilis* At3		√	Vegetable fields	46.5222	8.5178565	GCF_012647565.1	4197841	43.5	(235)
267	*Paenibacillus* sp. S02	√		Perennial ryegrass seed	-26.121	134.53605	GCF_019454065.1	6060529	47.2	(236)
268	*Paenibacillus* sp. S25	√		Perennial ryegrass seed	-26.121	134.53605	GCF_019454085.1	5958851	47.2	(236)
269	*Pantoea alhagi* LTYR-11Z	√		Wheat	43.8258	87.61694	GCF_002101395.1	4316301	53.6	(237)
270	*Azospirillum* sp. B510	√	√	Rice	38.5674	140.70243	GCF_000010725.1	7599738	63.6	(238)
271	*Methylobacterium aquaticum* MA-22A	√		Rice	miss	miss	GCF_001548015.1	7557960	51	(234)
272	*Bacillus toyonensis* BAC3151	√		Rice	38.5674	140.70243	GCF_001548015.1	7557960	69.9	(239)
273	*Leifsonia* sp. ku-ls	√		Rice	miss	miss	GCF_003367665.1	4103528	60	(240)
274	*Burkholderia ambifaria* BCC0192		√	Soybean	-9.8275	-52.88404	GCF_001275045.2	5740808	35.4	(16)
275	*Bdellovibrio bacteriovorus* SSB218315	√	√	Rice	19.8512	79.764622	GCF_003367665.1	4103528	69.65	(241)
276	*Arthrobacter* sp. ZXY2		√	Corn rhizophere	39.5509	-101.5633	GCF_902829785.1	7400790	66.9	(242)

277	*Burkholderia ambifaria* BCC0410		√	Maize	42.4779	12.67888	GCF_902829705.1	7382768	66.9	(16)
278	*Burkholderia ambifaria* BCC1041		√	Maize	42.4779	12.67888	GCF_902829815.1	7513631	66.9	(16)
279	*Burkholderia ambifaria* BCC1105		√	Maize	42.4779	12.67888	GCF_902829845.1	6300062	66.9	(16)
280	*Burkholderia ambifaria* BCC1107		√	Maize	42.4779	12.67888	GCF_902829855.1	7400371	66.9	(16)
281	*Burkholderia ambifaria* BCC1088		√	Maize	42.4779	12.67888	GCF_902829875.1	7484277	66.9	(16)
282	*Burkholderia ambifaria* BCC1095		√	Maize	42.4779	12.67888	GCF_902829885.1	7399154	66.9	(16)
283	*Burkholderia ambifaria* BCC1098		√	Maize	42.4779	12.67888	GCF_902829895.1	7382606	66.9	(16)

Table. S2

Adonis test between habitats for phylogenetically informed principal component analysis (phylo-PCA) of carbohydrate enzymes. There were significant differences in the K1 and K4 taxa between LA and SA habitats, and indicated significant differences in the separation of the three habitats($p \leq 0.05$). There were significant differences between all three habitats of the K2 taxon, and indicated significant differences in the separation of the three habitats; $p=0.001$. Neither of the three habitats was no significant differences in the K3 taxon, and also no significant relationship between the separation of the three habitats($p>0.05$).

Group	Pairs	R2	P.value
K1	LA vs OA	0.01030094	0.120
	LA vs SA	0.07393866	0.001
	OA vs SA	0.02287676	0.071
K2	LA vs OA	0.40492604	0.002
	LA vs SA	0.41157104	0.001
	OA vs SA	0.04091875	0.018
K3	LA vs OA	0.027792393	0.284
	LA vs SA	0.007707592	0.474
	OA vs SA	0.002981870	0.633
K4	LA vs OA	0.24790955	0.072
	LA vs SA	0.03052536	0.050
	OA vs SA	0.01841219	0.194

Table. S3

Adonis test between habitats for phylogenetically informed principal component analysis (phylo-PCA) of secondary metabolic clusters. There were significant differences between all three habitats of K1 taxon and K2 taxon; and the separation between the three habitats with significant differences(p<0.05). There is no significant relationship between the three habitats of taxon K3(p>0.05); and indicated the separation between the three habitats was also with no significant relationship, (p>0.05). There was a significant difference between the LA and SA habitats of taxon K4, (p<0.05), and a significant relationship between the separation of the three habitats, (p<0.05).

Group	Pairs	R2	P.value
K1	LA vs OA	0.05846311	0.001
	LA vs SA	0.09533931	0.001
	OA vs SA	0.02190289	0.021
K2	LA vs OA	0.29435190	0.001
	LA vs SA	0.23860352	0.001
	OA vs SA	0.5070757	0.003
K3	LA vs OA	0.030823065	0.271
	LA vs SA	0.002610629	0.977
	OA vs SA	0.016684150	0.155
K4	LA vs OA	0.04646692	0.579
	LA vs SA	0.04283389	0.007
	OA vs SA	0.01668685	0.150

Supplementary References

1. Balthazar C, Novinscak A, Cantin G, Joly DL, Filion M. 2022. Biocontrol Activity of *Bacillus* spp. and *Pseudomonas* spp. Against Botrytis cinerea and Other Cannabis Fungal Pathogens. *Phytopathology* 112:549-560.
2. Xu WF, Ren HS, Ou T, Lei T, Wei JH, Huang CS, Li T, Strobel G, Zhou ZY, Xie J. 2019. Genomic and Functional Characterization of the Endophytic *Bacillus subtilis* 7PJ-16 Strain, a Potential Biocontrol Agent of Mulberry Fruit Sclerotiniose. *Microb Ecol* 77:651-663.
3. Chen Y, Wang J, Yang N, Wen Z, Sun X, Chai Y, Ma Z. 2018. Wheat microbiome bacteria can reduce virulence of a plant pathogenic fungus by altering histone acetylation. *Nat Commun* 9:3429.
4. Li F-Z, Zeng Y-J, Zong M-H, Yang J-G, Lou W-Y. 2020. Bioprospecting of a novel endophytic *Bacillus velezensis* FZ06 from leaves of Camellia assamica: Production of three groups of lipopeptides and the inhibition against food spoilage microorganisms. *J Biotechnol* 323:42-53.
5. Cui G, Yin K, Lin N, Liang M, Huang C, Chang C, Xi P, Deng YZ. 2020. *Burkholderia gladioli* CGB10: A Novel Strain Biocontrolling the Sugarcane Smut Disease. *Microorganisms* 8:1943.
6. Li C, Cheng P, Zheng L, Li Y, Chen Y, Wen S, Yu G. 2021. Comparative genomics analysis of two banana Fusarium wilt biocontrol endophytes *Bacillus subtilis* R31 and TR21 provides insights into their differences on phytobeneficial trait. *Genomics* 113:900-909.
7. Wilson M, Lindow SE. 1993. INTERACTIONS BETWEEN THE BIOLOGICAL-CONTROL AGENT *PSEUDOMONAS-FLUORESCENS* A506 AND ERWINIA-AMYLOVORA IN PEAR BLOSSOMS. *Phytopathology* 83:117-123.
8. Xie S, Vallet M, Sun C, Kunert M, David A, Zhang XC, Chen BH, Lu XM, Boland W, Shao YQ. 2020. Biocontrol Potential of a Novel Endophytic *Bacterium* From Mulberry (Morus) Tree. *Front Bioeng Biotech* 7:488.
9. Zong Y, Zhao Y, Liu Y, Yang Q. 2018. Study on the Inhibitory Effect of *Bacillus velezensis* on Fusarium graminearum. *J Nucl Agri Sci* 32:310-317.
10. Kim JD, Jeon BJ, Han JW, Park MY, Kang SA, Kim BS. 2016. Evaluation of the endophytic nature of *Bacillus amyloliquefaciens* strain GYL4 and its efficacy in the control of anthracnose. *Pest Manag Sci* 72:1529-36.
11. Indiragandhi P, Anandham R, Kim K, Yim WJ, Madhaiyan M, Sa TM. 2008. Induction of defense responses in tomato against *Pseudomonas syringae* pv. tomato by regulating the stress ethylene level with *Methylobacterium oryzae* CBMB20 containing 1-aminocyclopropane-1-carboxylate deaminase. *World J Microb Biot* 24:1037-1045.
12. Zhang J, Xia M, Liu H, Zhang M, Mi G, Yang L. 2019. Efficacy of dazomet fumigation and bioorganic fertilizer in integrated control of cucumber root-knot nematode. *Acta Phytophylacica Sinica* 46:824-831.
13. Ali M, Walait S, Ul Haque MF, Mukhtar S. 2021. Antimicrobial activity of *bacteria* associated with the *rhizosphere* and *phyllosphere* of Avena fatua and Brachiaria reptans. *Environ Sci Pollut R* 28:68846-68861.
14. Helfrich EJN, Vogel CM, Ueoka R, Schaefer M, Ryffel F, Mueller DB, Probst S, Kreuzer M, Piel J, Vorholt JA. 2018. Bipartite interactions, antibiotic production and biosynthetic potential of the Arabidopsis leaf microbiome. *Nat Microb* 3:909-919.
15. Singh AK, Sharma RK, Sharma V, Singh T, Kumar R, Kumari D. 2017. Isolation, morphological identification and in vitro antibacterial activity of endophytic bacteria isolated from Azadirachta indica (neem) leaves. *Vet world* 10:510-516.
16. Mullins AJ, Murray JAH, Bull MJ, Jenner M, Jones C, Webster G, Green AE, Neill DR, Connor TR, Parkhill J, Challis GL, Mahenthiralingam E. 2019. Genome mining identifies cepacin as a plant-protective metabolite of the biopesticidal *bacterium Burkholderia ambifaria*. *Nat Microb* 4:996-1005.
17. Leveau JH, Lindow SE. 2005. Utilization of the plant hormone indole-3-acetic acid for growth by *Pseudomonas putida* strain 1290. *Appl Environ Microb* 71:2365-71.
18. Zhao Y, Selvaraj JN, Xing F, Zhou L, Wang Y, Song H, Tan X, Sun L, Sangare L, Folly YM, Liu Y. 2014. Antagonistic action of *Bacillus subtilis* strain SG6 on Fusarium graminearum. *PLoS One* 9:e92486.

19. Zeng Q, Xie J, Zhang X, Li Y, Wang Q. 2020. Complete genome sequence data of *Bacillus pumilus* GLB197, an effective antagonist of grape downy mildew. *Data Brief* 30:105423.

20. Lin YT, Lee CC, Leu WM, Wu JJ, Huang YC, Meng M. 2021. Fungicidal Activity of Volatile Organic Compounds Emitted by *Burkholderia gladioli* Strain BBB-01. *Molecules* 26:745.

21. Yim W, Seshadri S, Kim K, Lee G, Sa T. 2013. Ethylene emission and PR protein synthesis in ACC deaminase producing *Methylobacterium* spp. inoculated tomato plants (Lycopersicon esculentum Mill.) challenged with Ralstonia solanacearum under greenhouse conditions. *Plant Physiol Biochem* 67:95-104.

22. Caradonia F, Francia E, Morcia C, Ghizzoni R, Moulin L, Terzi V, Ronga D. 2019. Arbuscular Mycorrhizal Fungi and Plant Growth Promoting Rhizobacteria Avoid Processing Tomato Leaf Damage during Chilling Stress. *Agronomy-Basel* 9:299.

23. Deraz SF, Karlsson EN, Hedstrom M, Andersson MM, Mattiasson B. 2005. Purification and characterisation of acidocin D20079, a bacteriocin produced by *Lactobacillus acidophilus* DSM 20079. *J Biotech* 117:343-354.

24. Pandin C, Darsonval M, Mayeur C, Le Coq D, Aymerich S, Briandet R. 2019. Biofilm Formation and Synthesis of Antimicrobial Compounds by the Biocontrol Agent *Bacillus velezensis* QST713 in an Agaricus bisporus Compost Micromodel. *Appl Environ Microb* 85:e00327-19.

25. Ahire JJ, Dicks LMT. 2016. Antimicrobial Hyaluronic Acid-Cefoxitin Sodium Thin Films Produced by Electrospraying. *Curr Microbiol* 73:236-241.

26. Li X-Y, Wang Y-H, Yang J, Cui W-Y, He P-J, Munir S, He P-F, Wu Y-X, He Y-Q. 2018. Acaricidal Activity of Cyclodipeptides from *Bacillus amyloliquefaciens* W1 against Tetranychus urticae. *J Agric Food Chem* 66:10163-10168.

27. Han Y, Zhang B, Shen Q, You C, Yu Y, Li P, Shang Q. 2015. Purification and Identification of Two Antifungal Cyclic Peptides Produced by *Bacillus amyloliquefaciens* L-H15. *Appl Biochem Biotech* 176:2202-2212.

28. Li X, Zhang Y, Wei Z, Guan Z, Cai Y, Liao X. 2016. Antifungal Activity of Isolated *Bacillus amyloliquefaciens* SYBC H47 for the Biocontrol of Peach Gummosis. *Plos One* 11:e0162125.

29. Hsieh FC, Li MC, Lin TC, Kao SS. 2004. Rapid detection and characterization of surfactin-producing *Bacillus subtilis* and closely related species based on PCR. *Curr Microbiol* 49:186-191.

30. Zhang DF, Xiong XL, Wang YJ, Gao YX, Ren Y, Wang Q, Shi CB. 2021. *Bacillus velezensis* WLYS23 strain possesses antagonistic activity against hybrid snakehead bacterial pathogens. *J Appl Microbiol* 131:3056-3068.

31. Zhao M, Liu D, Liang Z, Huang K, Wu X. 2022. Antagonistic activity of *Bacillus subtilis* CW14 and its beta-glucanase against Aspergillus ochraceus. *Food Control* 131:108475.

32. He S, Feng K, Ding T, Huang K, Yan H, Liu X, Zhang Z. 2018. Complete genome sequence *Bacillus licheniformis* BL-010. *Microb Pathogenesis* 118:199-201.

33. Sharma D, Singh SS, Baindara P, Sharma S, Khatri N, Grover V, Patil PB, Korpole S. 2020. Surfactin Like Broad Spectrum Antimicrobial Lipopeptide Co-produced With Sublancin From *Bacillus subtilis* Strain A52: Dual Reservoir of Bioactives. *Front Microbiol* 11:1167.

34. Guo M, Liu D, Chai T. 2021. Complete Genome Sequence of *Bacillus subtilis* BYS2, a Strain with a Broad Inhibitory Spectrum against Pathogenic Bacteria. *Microbiol Resour Ann* 10:e0080321.

35. Tran TD, Del Cid C, Hnasko R, Gorski L, McGarvey JA. 2020. *Bacillus amyloliquefaciens* ALB65 Inhibits the Growth of Listeria monocytogenes on Cantaloupe Melons. *Appl Environ Microb* 87:e01926-20.

36. Wang DD, Li JH, Zhu GL, Zhao K, Jiang WW, Li HD, Wang WN, Kumar V, Dong SL, Zhu WM, Tian XL. 2020. Mechanism of the Potential Therapeutic Candidate *Bacillus subtilis* BSXE-1601 Against Shrimp Pathogenic Vibrios and Multifunctional Metabolites Biosynthetic Capability of the Strain as Predicted by Genome Analysis. *Front Microbiol* 11:581802.

37. Veshareh MJ, Azad EG, Deihimi T, Niazi A, Ayatollahi S. 2019. Isolation and screening of *Bacillus subtilis* MJ01 for MEOR application: biosurfactant characterization, production optimization and wetting effect on carbonate surfaces. *J Pet Explor Prod Te* 9:233-245.

38. Pan H-Q, Li Q-L, Hu J-C. 2017. The complete genome sequence of *Bacillus velezensis* 9912D reveals its biocontrol mechanism as a

novel commercial biological fungicide agent. *J Biotech* 247:25-28.

39. Shafique M, Jawaid A, Rehman Y. 2017. Redox biotransformation of arsenic along with plant growth promotion by multi-metal resistance *Pseudomonas* sp MX6. *CR Biol* 340:330-338.

40. Xiong Y, Yang R, Sun X, Yang H, Chen H. 2018. Effect of the epiphytic bacterium &ITBacillus&IT sp WPySW2 on the metabolism of &ITPyropia haitanensis&IT. *J Appl Phycology* 30:1225-1237.

41. Jung BK, Hong SJ, Jo HW, Jung Y, Park YJ, Park CE, Kim MC, Ibal JC, Roh DH, Shin JH. 2018. Genome sequencing to develop *Paenibacillus donghaensis* strain JH8(T) (KCTC 13049(T)=LMG 23780(T)) as a microbial fertilizer and correlation to its plant growth-promoting phenotype. *Mar Genomics* 37:39-42.

42. Rajkumari J, Chakraborty S, Pandey P. 2020. Distinctive features gleaned from the comparative genomes analysis of clinical and non-clinical isolates of *Klebsiella pneumoniae*. *Bioinformation* 16:256-268.

43. Harwood CR, Mouillon JM, Pohl S, Arnau J. 2018. Secondary metabolite production and the safety of industrially important members of the *Bacillus subtilis* group. *FEMS Microbiol Rev* 42:721-738.

44. Boong UT, Kim Yong-sang, Jung Jin-oh, Cho Sung-ho, Jung Do-yeon. 2012. Antimicrobial and Biogenic Amine-Degrading Activity of *Bacillus licheniformis* SCK B11 Isolated from Traditionally Fermented Red Pepper Paste. *Korean J Microb* 48:163-170.

45. Chung W-H, Kang J, Lim MY, Lim T-J, Lim S, Roh SW, Nam Y-D. 2018. Complete Genome Sequence and Genomic Characterization of *Lactobacillus acidophilus* LA1 (11869BP). *Front Pharmacol* 9:83.

46. Raittz RT, Reginatto De Pierri C, Maluk M, Bueno Batista M, Carmona M, Junghare M, Faoro H, Cruz LM, Battistoni F, Souza Ed, Pedrosa FdO, Chen W-M, Poole PS, Dixon RA, James EK. 2021. Comparative Genomics Provides Insights into the Taxonomy of Azoarcus and Reveals Separate Origins of Nif Genes in the Proposed Azoarcus and Aromatoleum Genera. *Genes* 12:71.

47. Park E-H, Sim H, Kim M-D. 2020. Complete genome sequence data of a broad- spectrum antipathogen, *Bacillus amyloliquefaciens* KCTC 18343P, isolated from Makgeolli, Korean traditional rice wine. *Data Brief* 29:105316.

48. Jeon S, Boong UT, Heo Jun. 2017. Complete genome sequence of *Bacillus subtilis* BS16045 isolated from Gochujang. *Korean Journal of Microbiology* 53:55-57.

49. Kim SY, Lee SY, Weon H-Y, Sang MK, Song J. 2017. Complete genome sequence of *Bacillus velezensis* M75, a biocontrol agent against fungal plant pathogens, isolated from cotton waste. *J Biotechnol* 241:112-115.

50. Kim J-e, Woo O-G, Bae Y, Keum HL, Chung S, Sul WJ, Lee J-N. 2020. Enhanced Drought and Salt Stress Tolerance in Arabidopsis by *Flavobacterium crocinum* HYN0056(T). *J Plant Biol* 63:63-71.

51. Choi HJ, Shin D, Shin M, Yun B, Kang M, Yang H-J, Jeong D-Y, Kim Y, Oh S. 2020. Comparative Genomic and Functional Evaluations of *Bacillus subtilis* Newly Isolated from Korean Traditional Fermented Foods. *Foods* 9:1805.

52. Li C, Zhang H, Xu Y, Liu W, Zhan L, Han X. 2010. Effect of Biocontrol Granules Preparations on Soybean Root Rot Disease in Field. *System Sciences and Comprehensive Studies in Agriculture* 26:330-334.

53. Vandamme P, Opelt K, Knöchel N, Berg C, Schönmann S, De Brandt E, Eberl L, Falsen E, Berg G. 2007. *Burkholderia bryophila* sp. nov. and *Burkholderia megapolitana* sp. nov., moss-associated species with antifungal and plant-growth-promoting properties. *Int J Syst Evol Microbiol* 57:2228-2235.

54. Mpofu E, Vejarano F, Suzuki-Minakuchi C, Ohtsubo Y, Tsuda M, Chakraborty J, Nakajima M, Okada K, Tada N, Kimura T, Nojiri H. 2019. Complete Genome Sequence of *Bacillus licheniformis* TAB7, a Compost-Deodorizing Strain with Potential for Plant Growth Promotion. *Microbiol Resour Ann* 8:e01659-18.

55. Hamdy AA, Elattal NA, Amin MA, Ali AE, Mansour NM, Awad GEA, Farrag ARH, Esawy MA. 2018. In vivo assessment of possible probiotic properties of *Bacillus subtilis* and prebiotic properties of levan. *Biocatalysis and Agricultural Biotechnology* 13:190-197.

56. Han S-R, Kim B, Jang JH, Park H, Oh T-J. 2021. Complete genome sequence of *Arthrobacter* sp. PAMC25564 and its comparative genome analysis for elucidating the role of CAZymes in cold adaptation. *Bmc Genomics* 22:403.

57. Zboralski A, Biessy A, Savoie M-C, Novinscak A, Filion M. 2020. Metabolic and Genomic Traits of Phytobeneficial Phenazine-Producing *Pseudomonas* spp. Are Linked to Rhizosphere Colonization in Arabidopsis thaliana and Solanum tuberosum. *Appl Environ Microb* 86:e02443-19.

58. Devi S, Kiesewalter HT, Kovacs R, Frisvad JC, Weber T, Larsen TO, Kovacs AT, Ding L. 2019. Depiction of secondary metabolites and antifungal activity of *Bacillus velezensis* DTU001. *Syn Syst Biotechno* 4:142-149.
59. Dischinger J, Josten M, Szekat C, Sahl HG, Bierbaum G. 2009. Production of the novel two-peptide lantibiotic lichenicidin by *Bacillus licheniformis* DSM 13. *PLoS One* 4:e6788.
60. Kang Y-H, Park C-S, Han M-S. 2012. *Pseudomonas aeruginosa* UCBPP-PA14 a useful bacterium capable of lysing Microcystis aeruginosa cells and degrading microcystins. *J Appl Phycology* 24:1517-1525.
61. Khatri I, Sharma S, Ramya TNC, Subramanian S. 2016. Complete Genomes of *Bacillus* coagulans S-lac and *Bacillus subtilis* TO-A JPC, Two Phylogenetically Distinct Probiotics. *Plos One* 11:e0156745.
62. Yabuuchi E, Kosako Y, Oyaizu H, Yano I, Hotta H, Hashimoto Y, Ezaki T, Arakawa M. 1992. PROPOSAL OF *BURKHOLDERIA* GEN-NOV AND TRANSFER OF 7 SPECIES OF THE GENUS *PSEUDOMONAS* HOMOLOGY GROUP-II TO THE NEW GENUS, WITH THE TYPE SPECIES *BURKHOLDERIA*-CEPACIA (PALLERONI AND HOLMES 1981) COMB-NOV. *Microbiol and Immunol* 36:1251-1275.
63. Douriet-Gamez NR, Maldonado-Mendoza IE, Ibarra-Laclette E, Blom J, Calderon-Vazquez CL. 2018. Genomic Analysis of *Bacillus* sp Strain B25, a Biocontrol Agent of Maize Pathogen Fusarium verticillioides. *Curr Microbiol* 75:247-255.
64. Qureshi KA, Seroor M, Al-Masabi A, Saykhan MA, Mutairi YA, Elhassan GO, Khan RA. 2020. Bio-characterizations of some *marine bacterial* strains isolated from mangrove sediment samples of four major cities of Saudi Arabia. *J Environ Biology* 41:1003-1012.
65. Samaras A, Nikolaidis M, Antequera-Gomez ML, Camara-Almiron J, Romero D, Moschakis T, Amoutzias GD, Karaoglanidis GS. 2021. Whole Genome Sequencing and Root Colonization Studies Reveal Novel Insights in the Biocontrol Potential and Growth Promotion by *Bacillus subtilis* MBI 600 on Cucumber. *Front Microbiol* 11:600393.
66. Romanenko LA, Uchino M, Tanaka N, Frolova GM, Slinkina NN, Mikhailov VV. 2008. Occurrence and antagonistic potential of *Stenotrophomonas* strains isolated from deep-sea invertebrates. *Arch Microbiol* 189:337-344.
67. Ma J, Jiang H, Li P, Li C, Liu R, Li J, Xiao Z, Pi B, Zhao M, Hu W, Liu G-Q. 2020. Production of free amino acid fertilizer from tung meal by the newly isolated *Pseudomonas aeruginosa* LYT-4 strain with simultaneous potential biocontrol capacity. *Renew Energ* 166:245-252.
68. You L-X, Zhang R-R, Dai J-X, Lin Z-T, Li Y-P, Herzberg M, Zhang J-L, Al-Wathnani H, Zhang C-K, Feng R-W, Liu H, Rensing C. 2021. Potential of cadmium resistant *Burkholderia contaminans* strain ZCC in promoting growth of soy beans in the presence of cadmium. *Ecotox Environ Safe* 211:111914.
69. Chen M, Wang J, Liu B, Zhu Y, Xiao R, Yang W, Ge C, Chen Z. 2020. Biocontrol of tomato bacterial wilt by the new strain *Bacillus velezensis* FJAT-46737 and its lipopeptides. *Bmc Microbiol* 20:160.
70. Anonymous. 2017. Culture Optimization for Solid-fermentation of *Bacillus licheniformis* FJAT-4 with Pig-raising Litters from Microbial Fermentation Bed. *Chinese Journal of Biological Control* 33:128-133.
71. Zhao Y, Zhang D, Yang Z, Zhu J, Zhao D, Xue X. 2020. Determination of the stability of fermentation broth and analysis of active components of *Bacillus velezensis* HN-Q-8. *Microbiology China* 47:490-499.
72. Wang XH, Onchari MM, Yang XT, Xu L, Yin XL, Wan FX, Chen YW, Guan M, Li B, Luo CP. 2022. Genome analysis of *Bacillus subtilis* JCL16 and the synergistic relationship among its metabolites reveal its potential for biocontrol of Nocardia seriolae. *Biol Control* 167:104855.
73. Faoro H, Menegazzo RR, Battistoni F, Gyaneshwar P, do Amaral FP, Taule C, Rausch S, Galvao PG, de los Santos C, Mitra S, Heijo G, Sheu S-Y, Chen W-M, Mareque C, Tadra-Sfeir MZ, Ivo Baldani J, Maluk M, Guimaraes AP, Stacey G, de Souza EM, Pedrosa FO, Cruz LM, James EK. 2017. The oil-contaminated soil diazotroph *Azoarcus olearius* DQS-4(T) is genetically and phenotypically similar to the model grass endophyte *Azoarcus* sp BH72. *Env Microbiol Rep* 9:223-238.
74. Woo O-G, Kim H, Kim J-S, Keum HL, Lee K-C, Sul WJ, Lee J-H. 2020. *Bacillus subtilis* strain GOT9 confers enhanced tolerance to drought and salt stresses in Arabidopsis thaliana and Brassica campestris. *Plant Physiol Bioch* 148:359-367.
75. Dutta S, Yu S-M, Nagendran R, Jeong S, Lee YH. 2019. Complete genome sequencing of *Pseudomonas fluorescens* NBC275, a biocontrol agent against fungal pathogens of plants and insects. *Korean Journal of Microbiology* 55:157-159.

76. Ge BB, Liu BH, Nwet TT, Zhao WJ, Shi LM, Zhang KC. 2016. *Bacillus methylotrophicus* Strain NKG-1, Isolated from Changbai Mountain, China, Has Potential Applications as a Biofertilizer or Biocontrol Agent. *Plos One* 11: e0166079.
77. Chauhan AK, Maheshwari DK, Bajpai VK. 2017. ISOLATION AND PRELIMINARY CHARACTERIZATION OF A BACTERIOCIN- PRODUCER *BACILLUS* STRAIN INHIBITING METHICILLIN RESISTANT STAPHYLOCOCCUS AUREUS. *Acta Biol Hung* 68:208-219.
78. Pacheco-Moreno A, Stefanato FL, Ford JJ, Trippel C, Uszkoreit S, Ferrafiat L, Grenga L, Dickens R, Kelly N, Kingdon ADH, Ambrosetti L, Nepogodiev SA, Findlay KC, Cheema J, Trick M, Chandra G, Tomalin G, Malone JG, Truman AW. 2021. Pan-genome analysis identifies intersecting roles for *Pseudomonas* specialized metabolites in potato pathogen inhibition. *Elife* 10:e71900.
79. Saber WIA, Ghoneem KM, Al-Askar AA, Rashad YM, Ali AA, Rashad EM. 2015. CHITINASE PRODUCTION BY *BACILLUS SUBTILIS* ATCC 11774 AND ITS EFFECT ON BIOCONTROL OF RHIZOCTONIA DISEASES OF POTATO. *Acta Biol Hung* 66:436-448.
80. Oyedara OO, Segura-Cabrera A, Guo X, Elufisan TO, Cantu Gonzalez RA, Perez MAR. 2018. Whole-Genome Sequencing and Comparative Genome Analysis Provided Insight into the Predatory Features and Genetic Diversity of Two *Bdellovibrio Species* Isolated from Soil. *Int J Genomics* 2018: 9402073.
81. Lu SE, Novak J, Austin FW, Gu GY, Ellis D, Kirk M, Wilson-Stanford S, Tonelli M, Smith L. 2009. Occidiofungin, a Unique Antifungal Glycopeptide Produced by a Strain of *Burkholderia* contaminans. *Biochemistry* 48:8312-8321.
82. Ma L, Qu S, Lin J, Jia J, Baird SM, Jiang N, Li H, Hou L, Lu S-E. 2019. The complete genome of the antifungal *bacterium Pseudomonas* sp. strain MS82. *J Plant Dis Protect* 126:153-160.
83. Holguin G, Bashan Y. 1996. Nitrogen-fixation by *Azospirillum brasilense* Cd is promoted when co-cultured with a mangrove *rhizosphere bacterium (Staphylococcus* sp.). *Soil Biol Biochem* 28:1651-1660.
84. Manuel Ruiz-Valdiviezo V, Antonio Rogel-Hernandez M, Guerrero G, Ivette Rincon-Molina C, Galdino Garcia-Perez L, Antonio Gutierrez-Miceli F, Jose Villalobos-Maldonado J, Lopez-Lopez A, Martinez-Romero E, Rincon-Rosales R. 2017. Complete Genome Sequence of a Novel Nonnodulating *Rhizobium* Species Isolated from Agave americana L. Rhizosphere. *Genome Announc* 5:e01280-17.
85. Zhang Z, Yin L, Li X, Zhang C, Liu C, Wu Z. 2018. The complete genome sequence of *Bacillus halotolerans* ZB201702 isolated from a drought- and salt-stressed rhizosphere soil. *Microb Pathogenesis* 123:246-249.
86. Le Thi Thanh T, Jaehne J, Pham Thi L, Le Thi Phuong T, Le Thi Kim C, Schneider A, Blumenscheit C, Lasch P, Schweder T, Borriss R. 2020. Draft Genome Sequences of 59 Endospore-Forming Gram-Positive *Bacteria* Associated with Crop Plants Grown in Vietnam. *Microbiol Resour Ann* 9: e01154-20.
87. Li JY, Gao TT, Wang Q. 2020. Comparative and Functional Analyses of Two Sequenced *Paenibacillus polymyxa* Genomes Provides Insights Into Their Potential Genes Related to Plant Growth-Promoting Features and Biocontrol Mechanisms. *Front Genet* 11:564939.
88. You M, Fang S, MacDonald J, Xu J, Yuan ZC. 2020. Isolation and characterization of *Burkholderia cenocepacia* CR318, a phosphate solubilizing bacterium promoting corn growth. *Microbiol Res* 233:126395.
89. Grady EN, MacDonald J, Ho MT, Weselowski B, McDowell T, Solomon O, Renaud J, Yuan ZC. 2019. Characterization and complete genome analysis of the surfactin-producing, plant-protecting bacterium *Bacillus velezensis* 9D-6. *BMC Microbiol* 19:5.
90. Laird M, Piccoli D, Weselowski B, McDowell T, Renaud J, MacDonald J, Yuan Z-C. 2020. Surfactin-producing *Bacillus velezensis* 1B-23 and *Bacillus* sp. 1D-12 protect tomato against bacterial canker caused by Clavibacter michiganensis subsp. *michiganensis. J Plant Pathol* 102:451-458.
91. Jimenez JA, Novinscak A, Filion M. 2020. *Pseudomonas fluorescens* LBUM677 differentially increases plant biomass, total oil content and lipid composition in three oilseed crops. *J Appl Microbiol* 128:1119-1127.
92. Hou Q, Wang C, Hou X, Xia Z, Ye J, Liu K, Liu H, Wang J, Guo H, Yu X, Yang Y, Du B, Ding Y. 2015. Draft Genome Sequence of *Brevibacillus brevis* DZQ7, a Plant Growth-Promoting Rhizobacterium with Broad-Spectrum Antimicrobial Activity. *Genome Announc* 3:e00831-15.
93. Ding HX, Mo WD, Yu S, Cheng HH, Peng LJ, Liu ZY. 2021. Whole Genome Sequence of *Bacillus velezensis* Strain GUMT319: A

Potential Biocontrol Agent Against Tobacco Black Shank Disease. *Front Microbiol* 12: 658113.

94. Liu H, Wang J, Sun HM, Han XB, Peng YL, Liu J, Liu K, Ding YQ, Wang CQ, Du BH. 2020. Transcriptome Profiles Reveal the Growth-Promoting Mechanisms of *Paenibacillus polymyxa* YC0136 on Tobacco (Nicotiana tabacumL.). *Front Microbiol* 11: 584174.

95. Liu H, Wang C, Li Y, Liu K, Hou Q, Xu W, Fan L, Zhao J, Gou J, Du B, Ding Y. 2017. Complete Genome Sequence of *Paenibacillus polymyxa* YC0573, a Plant Growth-Promoting Rhizobacterium with Antimicrobial Activity. *Genome Announc* 5:e01636-16.

96. Guo Q, Li Y, Lou Y, Shi M, Jiang Y, Zhou J, Sun Y, Xue Q, Lai H. 2019. *Bacillus amyloliquefaciens* Ba13 induces plant systemic resistance and improves rhizosphere microecology against tomato yellow leaf curl virus disease. *Appl Soil Ecol* 137:154-166.

97. Niu D-D, Liu H-X, Jiang C-H, Wang Y-P, Wang Q-Y, Jin H-L, Guo J-H. 2011. The Plant Growth-Promoting Rhizobacterium *Bacillus cereus* AR156 Induces Systemic Resistance in Arabidopsis thaliana by Simultaneously Activating Salicylate- and Jasmonate/Ethylene-Dependent Signaling Pathways. *Mol Plant Microbe In* 24:533-542.

98. Xu X, Xu M, Zhao Q, Xia Y, Chen C, Shen Z. 2018. Complete Genome Sequence of Cd(II)-Resistant *Arthrobacter* sp PGP41, a Plant Growth-Promoting Bacterium with Potential in Microbe-Assisted Phytoremediation. *Curr Microbiol* 75:1231-1239.

99. Zhao J, Liu H, Liu K, Li H, Peng Y, Liu J, Han X, Liu X, Yao L, Hou Q, Wang C, Ding Y, Du B. 2019. Complete Genome Sequence of *Bacillus velezensis* DSYZ, a Plant Growth-Promoting Rhizobacterium with Antifungal Properties. *Microbiol Resour Ann* 8 :e01217.

100. Wang M, Xing Y, Wang J, Xu Y, Wang G. 2014. The role of the chi1 gene from the endophytic bacteria *Serratia proteamaculans* 336x in the biological control of wheat take-all. *Canadian Journal of Microbiology* 60:533-540.

101. Liu F, Yang S, Xu F, Zhang Z, Lu Y, Zhang J, Wang G. 2022. Characteristics of biological control and mechanisms of *Pseudomonas chlororaphis* zm-1 against peanut stem rot. *Bmc Microbiol* 22 :9.

102. Zhao J, Zhao X, Wang J, Gong Q, Zhang X, Zhang G. 2020. Isolation, Identification and Characterization of Endophytic *Bacterium Rhizobium oryzihabitans* sp. nov., from Rice Root with Biotechnological Potential in Agriculture. *Microorganisms* 8:608.

103. Wang K, Li Y, Wu Y, Qiu Z, Ding Z, Wang X, Chen W, Wang R, Fu F, Rensing C, Yang G. 2020. Improved grain yield and lowered arsenic accumulation in rice plants by inoculation with arsenite-oxidizing *Achromobacter xylosoxidans* GD03. *Ecotox and Environ Safe* 206: 111229.

104. Chen L, Guo S, Tian X, Wang G, Shi H, Qu L. 2016. Identification of aroma-producing *fungus* GS-1 strain and its biocontrol efficacy against tomato gray mold. *Acta Phytophylacica Sinica* 43:608-613.

105. Guan Y, Zhu H, Zhu Y, Zhao H, Shu L, Song J, Yang X, Wu Z, Wu L, Yang M. 2022. Microbial consortium composed of *Cellulomonas* ZJW-6 and *Acinetobacter* DA-25 improves straw lignocellulose degradation. *Arch Microbiol* 204:139.

106. Tian D, Song X, Li C, Zhou W, Qin L, Wei L, Di W, Huang S, Li B, Huang Q, Long S, He Z, Wei S. 2021. Antifungal mechanism of *Bacillus amyloliquefaciens* strain GKT04 against Fusarium wilt revealed using genomic and transcriptomic analyses. *Microbiologyopen* 10:e1192.

107. Zeng Q, Shi GY, Nong ZM, Ye XL, Hu CJ. 2020. Complete Genome Sequence of *Pantoea ananatis* Strain NN08200, an Endophytic Bacterium Isolated from Sugarcane. *Curr Microbiol* 77:1864-1870.

108. Singh P, Singh RK, Guo D-J, Sharma A, Singh RN, Li D-P, Malviya MK, Song X-P, Lakshmanan P, Yang L-T, Li Y-R. 2021. Whole Genome Analysis of Sugarcane Root-Associated Endophyte *Pseudomonas aeruginosa* B18-A Plant Growth-Promoting Bacterium With Antagonistic Potential Against Sporisorium scitamineum. *Front Microbiol* 12 :628376.

109. Liu GQ, Kong YY, Fan YJ, Geng C, Peng DH, Sun M. 2017. Whole-genome sequencing of *Bacillus velezensis* LS69, a strain with a broad inhibitory spectrum against pathogenic bacteria. *J Biotechnol* 249:20-24.

110. Gu C, Wang Z, Wang L, Mao L, Song Q, Yang L, Xing Y, Li Y. 2018. gfp maker of nitrogen-fixing *bacteria* DX120E and inoculation effects on different sugarcane varieties. *Journal of Southern Argiculture* 49:1075-1081.

111. Wang Q, Zhang Y, Zhao J, Jing L, Zhou H. 2015. Research on Antibacterial Properties and Culture Conditions of *Bacillus subtillis* S-16. *Chinese Journal of Biological Control* 31:439-444.

112. Chen W, Wang Y, Li D, Li L, Xiao Q, Zhou Q. 2012. Draft Genome Sequence of *Brevibacillus brevis* Strain X23, a Biocontrol Agent against Bacterial Wilt. *J Bacteriol* 194:6634-6635.

113. Alberto Faria PS, Marques VdO, Romano Goncalves Selari PJ, Martins PF, Silva FG, Sales JdF. 2021. Multifunctional potential of

endophytic bacteria from Anacardium othonianum Rizzini in promoting in vitro and ex vitro plant growth. *Microbiol Res* 242: 126600.

114. Guo T, Liao M. 2014. Suppression of Rhizoctonia solani and induction of host plant resistance by *Paenibacillus kribbensis* PS04 towards controlling of rice sheath blight. *Biocontrol Sci Techn* 24:116-121.

115. Chen L, Shi H, Heng J, Wang D, Bian K. 2019. Antimicrobial, plant growth-promoting and genomic properties of the peanut endophyte *Bacillus velezensis* LDO2. *Microbiol Res* 218:41-48.

116. Xu W, Zhang LY, Goodwin PH, Xia MC, Zhang J, Wang Q, Liang J, Sun RH, Wu C, Yang LR. 2020. Isolation, Identification, and Complete Genome Assembly of an Endophytic *Bacillus velezensis* YB-130, Potential Biocontrol Agent Against Fusarium graminearum. *Front Microbiol* 11: 598285.

117. Jia Z, Jin W, Huang Y, Song S. 2017. Complete Genome Sequence of *Bacillus subtilis* J-5, a Potential Biocontrol Agent. *Genome Announ* 5:e00275-17.

118. Yi J, Zhang D, Cheng Y, Tan J, Luo Y. 2019. The impact of *Paenibacillus polymyxa* HY96-2 luxS on biofilm formation and control of tomato bacterial wilt. *Appl Microbiol Biotechnol* 103:9643-9657.

119. Zhou J, Xia F, Che S, Zhong L, Zhang G. 2019. Complete Genome Sequence of *Pantoea* sp. Strain CCBC3-3-1, an Antagonistic Endophytic Bacterium Isolated from a Cotinus coggygria Branch. *Microbiol Res Ann* 8 :e01004-19.

120. Zheng J, Yu J, Jia M, Zheng L, Feng Y. 2017. Indole enhances the survival of *Pantoea ananatis* YJ76 in face of starvation conditions. *J Basic Microb* 57:633-639.

121. Gao X, Ma M, Cao F, Zhu Z, Li J, Jiang X. 2021. Biocontrol potential of *Bacillus velezensis* 19573-3 identified using biochemistry and genomics methods. *Journal of Plant Nutrition and Fertitizer* 27:1068-1079.

122. Xu S, Xie X, Zhao Y, Shi Y, Chai A, Li L, Li B. 2020. Whole-genome analysis of *bacillus velezensis* ZF2, a biocontrol agent that protects cucumis sativus against corynespora leaf spot diseases. *3 Biotech* 10:186.

123. Peng J, Wu D, Liang Y, Li L, Guo Y. 2019. Disruption of acdS gene reduces plant growth promotion activity and maize saline stress resistance by *Rahnella aquatilis* HX2. *J Basic Microbiol* 59:402-411.

124. Li Z, Chen M, Ran K, Wang J, Zeng Q, Song F. 2018. Draft Genome Sequence of *Bacillus velezensis* Lzh-a42, a Plant Growth-Promoting Rhizobacterium Isolated from Tomato Rhizosphere. *Genome Announc* 6 :e00161-18.

125. Kong WJ, Yan YC, Li XY, Liu ZY. 2018. Draft Genome Sequence of *Bacillus velezensis* PEBA20, a Strain with a Plant Growth-Promoting Effect and Biocontrol Potential. *Genome Announc* 6: e00286-18.

126. Zhang Y, Li T, Liu Y, Li X, Zhang C, Feng Z, Peng X, Li Z, Qin S, Xing K. 2019. Volatile Organic Compounds Produced by *Pseudomonas chlororaphis* subsp. *aureofaciens* SPS-41 as Biological Fumigants To Control Ceratocystis fimbriata in Postharvest Sweet Potatoes. *J Agric Food Chem* 67:3702-3710.

127. Wu T, Xu J, Xie W, Yao Z, Yang H, Sun C, Li X. 2018. *Pseudomonas aeruginosa* L10: A Hydrocarbon-Degrading, Biosurfactant-Producing, and Plant-Growth-Promoting Endophytic Bacterium Isolated From a Reed (Phragmites australis). *Front Microbiol* 9:1087.

128. Wong-Villarreal A, Méndez-Santiago EW, Gómez-Rodríguez O, Aguilar-Marcelino L, García DC, García-Maldonado JQ, Hernández-Velázquez VM, Yañez-Ocampo G, Espinosa-Zaragoza S, S IR-G, Sanzón-Gómez D. 2021. Nematicidal Activity of the Endophyte *Serratia ureilytica* against Nacobbus aberrans in Chili Plants (Capsicum annuum L.) and Identification of Genes Related to Biological Control. *Plants (Basel)* 10 :2655.

129. Jin Y, Zhu H, Luo S, Yang W, Zhang L, Li S, Jin Q, Cao Q, Sun S, Xiao M. 2019. Role of Maize Root Exudates in Promotion of Colonization of *Bacillus velezensis* Strain S3-1 in Rhizosphere Soil and Root Tissue. *Curr Microbiol* 76:855-862.

130. Li D, Nie F, Wei L, Wei B, Chen Z. 2007. Screening of high-yielding biocontrol *bacterium* Bs-916 mutant by ion implantation. *Appl Microbiol Biotechnol* 75:1401-1408.

131. Vílchez JI, Tang Q, Kaushal R, Chen S, Liu R, Zhang H. 2018. Genome Sequence of *Bacillus cereus* Strain TG1-6, a Plant-Beneficial Rhizobacterium That Is Highly Salt Tolerant. *Genome Announc* 6: e00351-18.

132. Kiesewalter HT, Lozano-Andrade CN, Wibowo M, Strube ML, Maróti G, Snyder D, Jørgensen TS, Larsen TO, Cooper VS, Weber T, Kovács Á T. 2021. Genomic and Chemical Diversity of *Bacillus subtilis* Secondary Metabolites against Plant Pathogenic Fungi. *mSystems* 6: e00770-20.

133. Cheng M, Xu Q. 2017. Inhibitory mechanism of *Bacillus amyloliq uefaciens* subsp. *plantarum* CGMCC 11640 against Botryosphaeria dothidea, the pathogen of canker disease of Carya cathayensis. *Journal of Zhejiang a&f University* 34:326-331.
134. Shi Z, Ren D, Hu S, Hu X, Wu L, Lin H, Hu J, Zhang G, Guo L. 2015. Whole genome sequence of *Pseudomonas aeruginosa* F9676, an antagonistic bacterium isolated from rice seed. *J Biotechnol* 211:77-78.
135. Song D, Chen G, Liu S, Khaskheli MA, Wu L. 2019. Complete genome sequence of *Burkholderia* sp. JP2-270, a rhizosphere isolate of rice with antifungal activity against Rhizoctonia solani. *Microb Pathog* 127:1-6.
136. Gao G, Yin D, Chen S, Xia F, Yang J, Li Q, Wang W. 2012. Effect of biocontrol agent *Pseudomonas fluorescens* 2P24 on soil fungal community in cucumber rhizosphere using T-RFLP and DGGE. *PLoS One* 7:e31806.
137. Ho YN, Huang CC. 2015. Draft Genome Sequence of *Burkholderia cenocepacia* Strain 869T2, a Plant-Beneficial Endophytic Bacterium. *Genome Announc* 3: e01327-15.
138. Zhang L, Chen W, Jiang Q, Fei Z, Xiao M. 2020. Genome analysis of plant growth-promoting rhizobacterium *Pseudomonas chlororaphis* subsp. aurantiaca JD37 and insights from comparasion of genomics with three *Pseudomonas* strains. *Microbiol Res* 237:126483.
139. Hu S, Wang X, Sun W, Wang L, Li W. 2021. In Vitro Study of Biocontrol Potential of Rhizospheric *Pseudomonas aeruginosa* against Pathogenic Fungi of Saffron (Crocus sativus L.). *Pathogens* 10 :1423.
140. Yenn TW, Lee CC, Ibrahim D, Zakaria L. 2012. Enhancement of anti-candidal activity of endophytic fungus *Phomopsis* sp. ED2, isolated from Orthosiphon stamineus Benth, by incorporation of host plant extract in culture medium. *J Microbiol* 50:581-585.
141. Huang CJ, Tsay JF, Chang SY, Yang HP, Wu WS, Chen CY. 2012. Dimethyl disulfide is an induced systemic resistance elicitor produced by *Bacillus cereus* C1L. *Pest Manag Sci* 68:1306-1310.
142. Zhao CZ, Huang J, Gyaneshwar P, Zhao D. 2017. *Rhizobium* sp. IRBG74 Alters Arabidopsis Root Development by Affecting Auxin Signaling. *Front Microbiol* 8:2556.
143. Zhang C, Hao Q, Zhang S, Zhang Z, Zhang X, Sun P, Pan H, Zhang H, Sun F. 2019. Transcriptomic analysis of Chlorimuron-ethyl degrading *bacterial* strain *Klebsiella jilinsis* 2N3. *Ecotoxicol Environ Saf* 183:109581.
144. Nifakos K, Tsalgatidou PC, Thomloudi EE, Skagia A, Kotopoulis D, Baira E, Delis C, Papadimitriou K, Markellou E, Venieraki A, Katinakis P. 2021. Genomic Analysis and Secondary Metabolites Production of the Endophytic *Bacillus velezensis* Bvel1: A Biocontrol Agent against Botrytis cinerea Causing Bunch Rot in Post-Harvest Table Grapes. *Plants (Basel)* 10:1716.
145. Jeong JJ, Sajidah S, Oh JY, Sang MK, Kim KS, Kim KD. 2019. Complete genome sequence data of *Flavobacterium anhuiense* strain GSE09, a volatile-producing biocontrol bacterium isolated from cucumber (Cucumis sativus) root. *Data Brief* 25:104270.
146. Yu SM, Lee YH. 2015. Genes involved in nutrient competition by *Pseudomonas putida* JBC17 to suppress green mold in postharvest satsuma mandarin. *J Basic Microbiol* 55:898-906.
147. Lim SM, Yoon MY, Choi GJ, Choi YH, Jang KS, Shin TS, Park HW, Yu NH, Kim YH, Kim JC. 2017. Diffusible and Volatile Antifungal Compounds Produced by an Antagonistic *Bacillus velezensis* G341 against Various Phytopathogenic Fungi. *Plant Pathol* J 33:488-498.
148. Sukweenadhi J, Kim YJ, Choi ES, Koh SC, Lee SW, Kim YJ, Yang DC. 2015. *Paenibacillus yonginensis* DCY84(T) induces changes in Arabidopsis thaliana gene expression against aluminum, drought, and salt stress. *Microbiol Res* 172:7-15.
149. Hwangbo K, Um Y, Kim KY, Madhaiyan M, Sa TM, Lee Y. 2016. Complete Genome Sequence of *Bacillus velezensis* CBMB205, a Phosphate-Solubilizing Bacterium Isolated from the Rhizoplane of Rice in the Republic of Korea. *Genome Announc* 4 :e00654-16.
150. Kyung SM, Jae-Kyeong S, Kim Sang-yoon, Won Hang-yeon, Jeon Young-a, Ryu Jae-hwan. 2016. Characterization of *Multifunctional Bacillus* sp. GH1-13. *The Korean Journal of Pesticide Science* 20:189-196.
151. Cho HS, Park SY, Ryu CM, Kim JF, Kim JG, Park SH. 2007. Interference of quorum sensing and virulence of the rice pathogen *Burkholderia glumae* by an engineered endophytic bacterium. *FEMS Microbiol Ecol* 60:14-23.
152. Jung BK, Hong S-J, Park G-S, Kim M-C, Shin J-H. 2018. Isolation of *Burkholderia cepacia* JBK9 with plant growth-promoting activity while producing pyrrolnitrin antagonistic to plant fungal diseases. *Appl Biol Chem* 61:173-180.
153. Khan AR, Park GS, Asaf S, Hong SJ, Jung BK, Shin JH. 2017. Complete genome analysis of *Serratia marcescens* RSC-14: A plant

growth-promoting bacterium that alleviates cadmium stress in host plants. *PLoS One* 12:e0171534.
154. Im SM, Yu NH, Joen HW, Kim SO, Park HW, Park AR, Kim JC. 2020. Biological control of tomato bacterial wilt by oxydifficidin and difficidin-producing *Bacillus methylotrophicus* DR-08. *Pestic Biochem Physiol* 163:130-137.
155. Kim B, Kim YS, Han JW, Choi GJ, Kim H. 2021. Genome Sequence of *Brevibacillus brevis* HK544, an Antimicrobial Bacterium Isolated from Soil in Daejeon, South Korea. *Microbiol Resour Announc* 10:e0041721.
156. Son JS, Hwang YJ, Lee SY, Ghim SY. 2021. *Serratia rhizosphaerae* sp. nov., a novel plant resistance inducer against soft rot disease in tobacco. *Int J Syst Evol Microbiol* 71: 004788.
157. Nishu SD, No JH, Lee TK. 2022. Transcriptional Response and Plant Growth Promoting Activity of *Pseudomonas fluorescens* DR397 under Drought Stress Conditions. *Microbiol Spectr* 10:e0097922.
158. Kim YS, Lee Y, Cheon W, Park J, Kwon HT, Balaraju K, Kim J, Yoon YJ, Jeon Y. 2021. Characterization of *Bacillus velezensis* AK-0 as a biocontrol agent against apple bitter rot caused by Colletotrichum gloeosporioides. *Sci Rep* 11:626.
159. Yadav R, Ror P, Rathore P, Ramakrishna W. 2020. *Bacteria* from native soil in combination with arbuscular mycorrhizal fungi augment wheat yield and biofortification. *Plant Physiol Biochem* 150:222-233.
160. Yoo S-J, Weon H-Y, Song J, Sang MK. 2020. Effects of *Chryseobacterium soldanellicola* T16E-39 and *Bacillus siamensis* T20E-257 on Biocontrol against Phytophthora Blight and Bacterial Wilt and Growth Promotion in Tomato Plants. *Int J Agric Biol* 23:534-540.
161. Adam E, Müller H, Erlacher A, Berg G. 2016. Complete genome sequences of the *Serratia plymuthica* strains 3Rp8 and 3Re4-18, two rhizosphere bacteria with antagonistic activity towards fungal phytopathogens and plant growth promoting abilities. *Stand Genomic Sci* 11:61.
162. Zboralski A, Biessy A, Savoie MC, Novinscak A, Filion M. 2020. Metabolic and Genomic Traits of Phytobeneficial Phenazine-Producing *Pseudomonas* spp. Are Linked to Rhizosphere Colonization in Arabidopsis thaliana and Solanum tuberosum. *Appl Environ Microbiol* 86: e02443-19.
163. Nishizawa T, Tago K, Oshima K, Hattori M, Ishii S, Otsuka S, Senoo K. 2012. Complete genome sequence of the denitrifying and N2O-reducing *bacterium Azoarcus* sp. strain KH32C. *J Bacteriol* 194:1255.
164. Morohoshi T, Wang WZ, Suto T, Saito Y, Ito S, Someya N, Ikeda T. 2013. Phenazine antibiotic production and antifungal activity are regulated by multiple quorum-sensing systems in *Pseudomonas chlororaphis* subsp. *aurantiaca* StFRB508. *J Biosci Bioeng* 116:580-584.
165. Delestre C, Laugraud A, Ridgway H, Ronson C, O'Callaghan M, Barrett B, Ballard R, Griffiths A, Young S, Blond C, Gerard E, Wakelin S. 2015. Genome sequence of the clover symbiont *Rhizobium leguminosarum bv. trifolii* strain CC275e. *Stand Genomic Sci* 10:121.
166. Petersen I, Paungfoo-Lonhienne C, Marcellin E, Nielsen LK, Gonzalez A. 2021. Towards Sustainable Bioinoculants: A Fermentation Strategy for High Cell Density Cultivation of *Paraburkholderia* sp. SOS3, a Plant Growth-Promoting Bacterium Isolated in Queensland, Australia. *Fermentation-Basel* 7:58.
167. Hamaoka K, Aoki Y, Suzuki S. 2021. Isolation and Characterization of Endophyte *Bacillus velezensis* KOF112 from Grapevine Shoot Xylem as Biological Control Agent for Fungal Diseases. *Plants (Basel)* 10:1815.
168. Gao N, Shen W, Nishizawa T, Isobe K, Guo Y, Ying H, Senoo K. 2019. Genome Sequences of Two *Azospirillum* sp. Strains, TSA2S and TSH100, Plant Growth-Promoting Rhizobacteria with N(2)O Mitigation Abilities. *Microbiol Resour Announc* 8: e00459-19.
169. Shen W, Yu X, Gao N, Ota S, Shiratori Y, Nishizawa T, Isobe K, He X, Senoo K. 2019. Genome Sequence of *Arthrobacter* sp. UKPF54-2, a Plant Growth-Promoting *Rhizobacterial* Strain Isolated from Paddy Soil. *Microbiol Resour Announc* 8: e01005-19.
170. Kanehara K, Minamisawa K. 2017. Complete Genome Sequence of *Bradyrhizobium japonicum* J5, Isolated from a Soybean Nodule in Hokkaido, Japan. *Genome Announc* 5: e01619-16.
171. Reeve W, O'Hara G, Chain P, Ardley J, Bräu L, Nandesena K, Tiwari R, Copeland A, Nolan M, Han C, Brettin T, Land M, Ovchinikova G, Ivanova N, Mavromatis K, Markowitz V, Kyrpides N, Melino V, Denton M, Yates R, Howieson J. 2010. Complete genome sequence of *Rhizobium leguminosarum* bv. *trifolii* strain WSM1325, an effective microsymbiont of annual Mediterranean clovers. *Stand Genomic Sci* 2:347-356.

172. Bóka B, Manczinger L, Kocsubé S, Shine K, Alharbi NS, Khaled JM, Münsterkötter M, Vágvölgyi C, Kredics L. 2019. Genome analysis of a *Bacillus subtilis* strain reveals genetic mutations determining biocontrol properties. *World J Microbiol Biotechnol* 35:52.
173. Nihorimbere V, Ongena M, Cawoy H, Henry G, Brostaux Y, Kakana P, Thonart P. 2009. *Bacillus*-based biocontrol of fusarium disease on tomato cultures in Burundi. *Commun Agric Appl Biol Sci* 74:645-649.
174. Fernández H, Prandoni N, Fernández-Pascual M, Fajardo S, Morcillo C, Díaz E, Carmona M. 2014. *Azoarcus* sp. CIB, an anaerobic biodegrader of aromatic compounds shows an endophytic lifestyle. *PLoS One* 9:e110771.
175. Chen Y-Y, Chen P-C, Tsay T-T. 2016. The biocontrol efficacy and antibiotic activity of Streptomyces plicatus on the oomycete Phytophthora capsici. *Biological Control* 98:34-42.
176. Eida AA, Bougouffa S, Alam I, Hirt H, Saad MM. 2021. Complete Genome Sequence of *Cellulomonas* sp. JZ18, a Root Endophytic Bacterium Isolated from the Perennial Desert Tussock-Grass Panicum turgidum. *Curr Microbiol* 78:1135-1141.
177. Carrión VJ, Cordovez V, Tyc O, Etalo DW, de Bruijn I, de Jager VCL, Medema MH, Eberl L, Raaijmakers JM. 2018. Involvement of *Burkholderiaceae* and sulfurous volatiles in disease-suppressive soils. *Isme j* 12:2307-2321.
178. Beneduzi A, Costa PB, Parma M, Melo IS, Bodanese-Zanettini MH, Passaglia LMP. 2010. *Paenibacillus riograndensis* sp. nov., a nitrogen-fixing species isolated from the *rhizosphere* of Triticum aestivum. *Int J Syst Evol Microbiol* 60:128-133.
179. Quecine MC, Araújo WL, Rossetto PB, Ferreira A, Tsui S, Lacava PT, Mondin M, Azevedo JL, Pizzirani-Kleiner AA. 2012. Sugarcane growth promotion by the endophytic *bacterium Pantoea agglomerans* 33.1. *Appl Environ Microbiol* 78:7511-7518.
180. Biessy A, Novinscak A, St-Onge R, Léger G, Zboralski A, Filion M. 2021. Inhibition of Three Potato Pathogens by Phenazine-Producing *Pseudomonas* spp. Is Associated with Multiple Biocontrol-Related Traits. *mSphere* 6:e0042721.
181. Lally RD, Galbally P, Moreira AS, Spink J, Ryan D, Germaine KJ, Dowling DN. 2017. Application of Endophytic *Pseudomonas fluorescens* and a Bacterial Consortium to Brassica napus Can Increase Plant Height and Biomass under Greenhouse and Field Conditions. *Front Plant Sci* 8:2193.
182. Rivera D, Revale S, Molina R, Gualpa J, Puente M, Maroniche G, Paris G, Baker D, Clavijo B, McLay K, Spaepen S, Perticari A, Vazquez M, Wisniewski-Dyé F, Watkins C, Martínez-Abarca F, Vanderleyden J, Cassán F. 2014. Complete Genome Sequence of the Model *Rhizosphere* Strain *Azospirillum brasilense* Az39, Successfully Applied in Agriculture. *Genome Announc* 2: e00683-14.
183. Cassan F, Perrig D, Sgroy V, Masciarelli O, Penna C, Luna V. 2009. *Azospirillum brasilense* Az39 and *Bradyrhizobium japonicum* E109, inoculated singly or in combination, promote seed germination and early seedling growth in corn (Zea mays L.) and soybean (Glycine max L.). *European Journal of Soil Biology* 45:28-35.
184. Alvarez F, Simonetti E, Draghi WO, Vinacour M, Palumbo MC, Do Porto DF, Montecchia MS, Roberts IN, Ruiz JA. 2022. Genome mining of *Burkholderia ambifaria* strain T16, a rhizobacterium able to produce antimicrobial compounds and degrade the mycotoxin fusaric acid. *World J Microb Biot* 38 :114.
185. Faoro H, Rene Menegazzo R, Battistoni F, Gyaneshwar P, do Amaral FP, Taulé C, Rausch S, Gonçalves Galvão P, de Los Santos C, Mitra S, Heijo G, Sheu SY, Chen WM, Mareque C, Zibetti Tadra-Sfeir M, Ivo Baldani J, Maluk M, Paula Guimarães A, Stacey G, de Souza EM, Pedrosa FO, Magalhães Cruz L, James EK. 2017. The oil-contaminated soil diazotroph *Azoarcus olearius* DQS-4(T) is genetically and phenotypically similar to the model grass endophyte *Azoarcus* sp. BH72. *Environ Microbiol Rep* 9:223-238.
186. Mehnaz S, Bechthold A, Gross H. 2020. Draft Genome Sequence of *Pseudomonas chlororaphis* subsp. *aurantiaca* ARS-38, a Bacterial Strain with Plant Growth Promotion Potential, Isolated from the Rhizosphere of Cotton in Pakistan. *Microbiol Resour Announc* 9 :e01398-19.
187. Singh C, Pandey P, Singh DN, Pandey R, Shasany AK, Tripathi AK. 2019. Whole-Genome Sequences of Four Indian Isolates of *Azospirillum brasilense*. *Microbiol Resour Announc* 8 :e00633-19.
188. Ferreira LC, Maul JE, Viana MVC, de Sousa TJ, de Carvalho Azevedo VA, Roberts DP, de Souza JT. 2021. Complete genome sequence of the biocontrol agent *Serratia marcescens* strain N4-5 uncovers an assembly artefact. *Braz J Microbiol* 52:245-250.
189. Huo Y, Kang JP, Kim YJ, Yang DC. 2018. *Paraburkholderia panacihumi* sp. nov., an isolate from ginseng-cultivated soil, is antagonistic against root rot fungal pathogen. *Arch Microbiol* 200:1151-1158.
190. Huang L, Li F, Yang Z, Bai L. 2015. Biocontrol efficacy of *Phomopsis* sp strain S4 against Sclerotinia sclerotiorum. *Chinese Journal*

of Applied and Environmental Biology 21:1090-1094.

191. Rajkumari J, Paikhomba Singha L, Pandey P. 2018. Genomic insights of aromatic hydrocarbon degrading *Klebsiella pneumoniae* AWD5 with plant growth promoting attributes: a paradigm of soil isolate with elements of biodegradation. *3 Biotech* 8:118.
192. Hunziker L, Bönisch D, Groenhagen U, Bailly A, Schulz S, Weisskopf L. 2015. *Pseudomonas* strains naturally associated with potato plants produce volatiles with high potential for inhibition of Phytophthora infestans. *Appl Environ Microbiol* 81:821-830.
193. Guleria S, Walia A, Chauhan A, Shirkot CK. 2016. Molecular characterization of alkaline protease of *Bacillus amyloliquefaciens* SP1 involved in biocontrol of Fusarium oxysporum. *Int J Food Microbiol* 232:134-143.
194. Sood U, Singh DN, Hira P, Lee JK, Kalia VC, Lal R, Shakarad M. 2020. Rapid and solitary production of mono-rhamnolipid biosurfactant and biofilm inhibiting pyocyanin by a taxonomic outlier *Pseudomonas aeruginosa* strain CR1. *J Biotechnol* 307:98-106.
195. Sibponkrung S, Kondo T, Tanaka K, Tittabutr P, Boonkerd N, Yoshida KI, Teaumroong N. 2020. Co-Inoculation of *Bacillus velezensis* Strain S141 and *Bradyrhizobium* Strains Promotes Nodule Growth and Nitrogen Fixation. *Microorganisms* 8:678.
196. Zhang L, Zhong J, Liu H, Xin K, Chen C, Li Q, Wei Y, Wang Y, Chen F, Shen X. 2017. Complete genome sequence of the drought resistance-promoting endophyte *Klebsiella* sp. LTGPAF-6F. *J Biotechnol* 246:36-39.
197. Liu H, Zeng Q, Yalimaimaiti N, Wang W, Zhang R, Yao J. 2021. Comprehensive genomic analysis of *Bacillus velezensis* AL7 reveals its biocontrol potential against Verticillium wilt of cotton. *Mol Genet Genomics* 296:1287-1298.
198. Xu P, Xie S, Liu W, Jin P, Wei D, Yaseen DG, Wang Y, Miao W. 2020. Comparative Genomics Analysis Provides New Strategies for Bacteriostatic Ability of *Bacillus velezensis* HAB-2. *Front Microbiol* 11:594079.
199. Jia J, Ford E, Baird SM, Lu SE. 2021. Complete Genome Sequence Resource for the Endophytic *Burkholderia* sp. Strain MS389 Isolated from a Healthy Soybean Growing Adjacent to Charcoal Rot Disease Patch. *Plant Dis* 105:2704-2707.
200. Jia J, Ford E, Hobbs SM, Baird SM, Lu SE. 2022. Occidiofungin Is the Key Metabolite for Antifungal Activity of the Endophytic *Bacterium Burkholderia* sp. MS455 Against Aspergillus flavus. *Phytopathology* 112:481-491.
201. Dueholm MS, Søndergaard MT, Nilsson M, Christiansen G, Stensballe A, Overgaard MT, Givskov M, Tolker-Nielsen T, Otzen DE, Nielsen PH. 2013. Expression of Fap amyloids in *Pseudomonas aeruginosa*, *P. fluorescens*, and *P. putida* results in aggregation and increased biofilm formation. *Microbiologyopen* 2:365-382.
202. Rashid MHO, Young JPW, Everall I, Clercx P, Willems A, Braun MS, Wink M. 2015. Average nucleotide identity of genome sequences supports the description of *Rhizobium* lentis sp nov., *Rhizobium bangladeshense* sp nov and *Rhizobium binae* sp nov from lentil (Lens culinaris) nodules. *Int J Syst Evol Micr* 65:3037-3045.
203. Garbeva P, Silby MW, Raaijmakers JM, Levy SB, Boer W. 2011. Transcriptional and antagonistic responses of *Pseudomonas fluorescens* Pf0-1 to phylogenetically different bacterial competitors. *Isme j* 5:973-985.
204. Fan B, Wang C, Song X, Ding X, Wu L, Wu H, Gao X, Borriss R. 2018. *Bacillus velezensis* FZB42 in 2018: The Gram-Positive Model Strain for Plant Growth Promotion and Biocontrol. *Front Microbiol* 9:2491.
205. Esmaeel Q, Miotto L, Rondeau M, Leclère V, Clément C, Jacquard C, Sanchez L, Barka EA. 2018. *Paraburkholderia phytofirmans* PsJN-Plants Interaction: From Perception to the Induced Mechanisms. *Front Microbiol* 9:2093.
206. Cartieaux F, Contesto C, Gallou A, Desbrosses G, Kopka J, Taconnat L, Renou JP, Touraine B. 2008. Simultaneous interaction of Arabidopsis thaliana with *Bradyrhizobium* Sp. strain ORS278 and *Pseudomonas syringae* pv. *tomato* DC3000 leads to complex transcriptome changes. *Mol Plant Microbe Interact* 21:244-259.
207. Jeong H, Choi SK, Ryu CM, Park SH. 2019. Chronicle of a Soil Bacterium: *Paenibacillus polymyxa* E681 as a Tiny Guardian of Plant and Human Health. *Front Microbiol* 10:467.
208. Smits TH, Rezzonico F, Kamber T, Goesmann A, Ishimaru CA, Stockwell VO, Frey JE, Duffy B. 2010. Genome sequence of the biocontrol agent *Pantoea vagans* strain C9-1. *J Bacteriol* 192:6486-6487.
209. Liu H, Li Y, Ge K, Du B, Liu K, Wang C, Ding Y. 2021. Interactional mechanisms of *Paenibacillus polymyxa* SC2 and pepper (Capsicum annuum L.) suggested by transcriptomics. *BMC Microbiol* 21:70.
210. Strnad H, Ridl J, Paces J, Kolar M, Vlcek C, Paces V. 2011. Complete genome sequence of the haloaromatic acid-degrading bacterium

Achromobacter xylosoxidans A8. *J Bacteriol* 193:791-792.
211. Kai M, Effmert U, Berg G, Piechulla B. 2007. Volatiles of bacterial antagonists inhibit mycelial growth of the plant pathogen Rhizoctonia solani. *Arch Microbiol* 187:351-360.
212. Matilla MA, Pizarro-Tobias P, Roca A, Fernández M, Duque E, Molina L, Wu X, van der Lelie D, Gómez MJ, Segura A, Ramos JL. 2011. Complete genome of the plant growth-promoting *rhizobacterium Pseudomonas putida* BIRD-1. *J Bacteriol* 193:1290.
213. Abebe-Akele F, Tisa LS, Cooper VS, Hatcher PJ, Abebe E, Thomas WK. 2015. Genome sequence and comparative analysis of a putative entomopathogenic *Serratia* isolated from Caenorhabditis briggsae. *BMC Genomics* 16:531.
214. Neupane S, Finlay RD, Alström S, Elfstrand M, Högberg N. 2015. Transcriptional responses of the bacterial antagonist *Serratia plymuthica* to the fungal phytopathogen Rhizoctonia solani. *Environ Microbiol Rep* 7:123-127.
215. Wu DQ, Ye J, Ou HY, Wei X, Huang X, He YW, Xu Y. 2011. Genomic analysis and temperature-dependent transcriptome profiles of the rhizosphere originating strain *Pseudomonas aeruginosa* M18. *BMC Genomics* 12:438.
216. Mülner P, Schwarz E, Dietel K, Herfort S, Jähne J, Lasch P, Cernava T, Berg G, Vater J. 2021. Fusaricidins, Polymyxins and Volatiles Produced by *Paenibacillus polymyxa* Strains DSM 32871 and M1. *Pathogens* 10:1485.
217. Drogue B, Sanguin H, Borland S, Prigent-Combaret C, Wisniewski-Dyé F. 2014. Genome wide profiling of *Azospirillum lipoferum* 4B gene expression during interaction with rice roots. *FEMS Microbiol Ecol* 87:543-55.
218. Okubo T, Fukushima S, Itakura M, Oshima K, Longtonglang A, Teaumroong N, Mitsui H, Hattori M, Hattori R, Hattori T, Minamisawa K. 2013. Genome analysis suggests that the soil oligotrophic *bacterium Agromonas oligotrophica* (*Bradyrhizobium oligotrophicum*) is a nitrogen-fixing symbiont of Aeschynomene indica. *Appl Environ Microbiol* 79:2542-2551.
219. Weselowski B, Nathoo N, Eastman AW, MacDonald J, Yuan ZC. 2016. Isolation, identification and characterization of *Paenibacillus polymyxa* CR1 with potentials for biopesticide, biofertilization, biomass degradation and biofuel production. *BMC Microbiol* 16:244.
220. Raza W, Yang X, Wu H, Wang Y, Xu Y, Shen Q. 2009. Isolation and characterisation of fusaricidin-type compound-producing strain of *Paenibacillus polymyxa* SQR-21 active against Fusarium oxysporum f. sp nevium. *European Journal of Plant Pathology* 125:471-483.
221. Huang R, Feng H, Xu Z, Zhang N, Liu Y, Shao J, Shen Q, Zhang R. 2022. Identification of Adhesins in Plant Beneficial *Rhizobacteria Bacillus velezensis* SQR9 and Their Effect on Root Colonization. *Mol Plant Microbe Interact* 35:64-72.
222. Li P, Kwok AH, Jiang J, Ran T, Xu D, Wang W, Leung FC. 2015. Comparative genome analyses of *Serratia marcescens* FS14 reveals its high antagonistic potential. *PLoS One* 10:e0123061.
223. Ramarathnam R, Bo S, Chen Y, Fernando WG, Xuewen G, de Kievit T. 2007. Molecular and biochemical detection of fengycin- and bacillomycin D-producing *Bacillus* spp., antagonistic to fungal pathogens of canola and wheat. *Can J Microbiol* 53:901-911.
224. Okigbo RN, Osuinde MI. 2003. Fungal leaf spot diseases of mango (Mangifera indica L.) in Southeastern Nigeria and biological control with *Bacillus subtilis*. *Plant Protect Sci* 39:70-77.
225. Caballero-Mellado J, Martínez-Aguilar L, Paredes-Valdez G, Santos PEL. 2004. *Burkholderia unamae* sp. nov., an N2-fixing rhizospheric and endophytic species. *Int J Syst Evol Microbiol* 54:1165-1172.
226. Perin L, Martínez-Aguilar L, Paredes-Valdez G, Baldani JI, Estrada-de Los Santos P, Reis VM, Caballero-Mellado J. 2006. *Burkholderia silvatlantica* sp. nov., a diazotrophic bacterium associated with sugar cane and maize. *Int J Syst Evol Microbiol* 56:1931-1937.
227. Kumar R, Borker SS, Thakur A, Thapa P, Kumar S, Mukhia S, Anu K, Bhattacharya A, Kumar S. 2021. Physiological and genomic evidence supports the role of *Serratia quinivorans* PKL:12 as a biopriming agent for the biohardening of micropropagated Picrorhiza kurroa plantlets in cold regions. *Genomics* 113:1448-1457.
228. Michiels KW, Croes CL, Vanderleyden J. 1991. 2 DIFFERENT MODES OF ATTACHMENT OF *AZOSPIRILLUM-BRASILENSE*-SP7 TO WHEAT ROOTS. *J Gener Microbiol* 137:2241-2246.
229. Govindasamy V, George P, Aher L, Ramesh SV, Thangasamy A, Anandan S, Raina SK, Kumar M, Rane J, Annapurna K, Minhas PS. 2017. Comparative conventional and phenomics approaches to assess symbiotic effectiveness of *Bradyrhizobia* strains in soybean (Glycine max L. Merrill) to drought. *Sci Rep* 7:6958.

230. Ilahi H, Hsouna J, Ellouze W, Gritli T, Chihaoui SA, Barhoumi F, Najib Elfeddy M, Bachkouel S, Ouahmane L, Tambong JT, Mnasri B. 2021. Phylogenetic study of rhizobia nodulating pea (Pisum sativum) isolated from different geographic locations in Tunisia. *Syst Appl Microbiol* 44:126221.

231. Rojas-Rojas FU, Salazar-Gómez A, Vargas-Díaz ME, Vásquez-Murrieta MS, Hirsch AM, De Mot R, Ghequire MGK, Ibarra JA, Estrada-de Los Santos P. 2018. Broad-spectrum antimicrobial activity by *Burkholderia cenocepacia* TAtl-371, a strain isolated from the tomato rhizosphere. *Microbiology* 164:1072-1086.

232. Tashan H, Harighi B, Rostamzadeh J, Azizi A. 2021. Characterization of Arsenic-Resistant Endophytic Bacteria From Alfalfa and Chickpea Plants. *Front Plant Sci* 12:696750.

233. Yuan L, Li L, Zheng F, Shi Y, Xie X, Chai A, Li B. 2020. The complete genome sequence of *Rahnella aquatilis* ZF7 reveals potential beneficial properties and stress tolerance capabilities. *Arch Microbiol* 202:483-499.

234. Tani A, Sahin N, Fujitani Y, Kato A, Sato K, Kimbara K. 2015. *Methylobacterium* Species Promoting Rice and Barley Growth and Interaction Specificity Revealed with Whole-Cell Matrix-Assisted Laser Desorption/Ionization-Time-of-Flight Mass Spectrometry (MALDI-TOF/MS) Analysis. *PLoS One* 10:e0129509.

235. Brandsch R, Mihasan M. 2020. A soil bacterial catabolic pathway on the move: Transfer of nicotine catabolic genes between Arthrobacter genus megaplasmids and invasion by mobile elements. *J Biosciences* 45:58.

236. Li T, Mann R, Kaur J, Spangenberg G, Sawbridge T. 2021. Transcriptomics differentiate two novel bioactive strains of *Paenibacillus* sp. isolated from the perennial ryegrass seed microbiome. *Sci Rep* 11:15545.

237. Chen C, Xin K, Liu H, Cheng J, Shen X, Wang Y, Zhang L. 2017. Pantoea alhagi, a novel endophytic bacterium with ability to improve growth and drought tolerance in wheat. *Sci Rep* 7:41564.

238. Kaneko T, Minamisawa K, Isawa T, Nakatsukasa H, Mitsui H, Kawaharada Y, Nakamura Y, Watanabe A, Kawashima K, Ono A, Shimizu Y, Takahashi C, Minami C, Fujishiro T, Kohara M, Katoh M, Nakazaki N, Nakayama S, Yamada M, Tabata S, Sato S. 2010. Complete genomic structure of the cultivated rice endophyte *Azospirillum* sp. B510. *DNA Res* 17:37-50.

239. Lopes R, Cerdeira L, Tavares GS, Ruiz JC, Blom J, Horácio ECA, Mantovani HC, Queiroz MV. 2017. Genome analysis reveals insights of the endophytic *Bacillus toyonensis* BAC3151 as a potentially novel agent for biocontrol of plant pathogens. *World J Microbiol Biotechnol* 33:185.

240. Battu L, Ulaganathan K. 2020. Whole genome sequencing and identification of host-interactive genes in the rice endophytic *Leifsonia* sp. ku-ls. *Funct Integr Genomics* 20:237-243.

241. Oyedara OO, De Luna-Santillana EJ, Olguin-Rodriguez O, Guo X, Mendoza-Villa MA, Menchaca-Arredondo JL, Elufisan TO, Garza-Hernandez JA, Garcia Leon I, Rodriguez-Perez MA. 2016. Isolation of *Bdellovibrio* sp. from soil samples in Mexico and their potential applications in control of pathogens. *Microbiologyopen* 5:992-1002.

242. Al-Daghari DSS, Al-Sadi AM, Janke R, Al-Mahmooli IH, Velazhahan R. 2020. Potential of indigenous antagonistic rhizobacteria in the biological control of Monosporascus root rot and vine decline disease of muskmelon. *Acta Agr Scand B-S P* 70:371-380.
